# Long noncoding RNA *ZFP36L2-AS* functions as a metabolic modulator to regulate muscle development

**DOI:** 10.1038/s41419-022-04772-2

**Published:** 2022-04-21

**Authors:** Bolin Cai, Manting Ma, Jing Zhang, Shaofen Kong, Zhen Zhou, Zhenhui Li, Bahareldin Ali Abdalla, Haiping Xu, Xiquan Zhang, Raman Akinyanju Lawal, Qinghua Nie

**Affiliations:** 1 Lingnan Guangdong Laboratory of Modern Agriculture & State Key Laboratory for Conservation and Utilization of Subtropical Agro-bioresources, College of Animal Science, South China Agricultural University, Guangzhou, 510642 Guangdong China; 2 Guangdong Provincial Key Lab of Agro-Animal Genomics and Molecular Breeding, and Key Laboratory of Chicken Genetics, Breeding and Reproduction, Ministry of Agriculture, Guangzhou, 510642 Guangdong China; 3 The Jackson Laboratory, 600 Main Street, Bar Harbor, ME US

**Keywords:** Cell proliferation, Differentiation

## Abstract

Skeletal muscle is the largest metabolic organ in the body, and its metabolic flexibility is essential for maintaining systemic energy homeostasis. Metabolic inflexibility in muscles is a dominant cause of various metabolic disorders, impeding muscle development. In our previous study, we found lncRNA *ZFP36L2-AS* (for “*ZFP36L2*-antisense transcript”) is specifically enriched in skeletal muscle. Here, we report that *ZFP36L2-AS* is upregulated during myogenic differentiation, and highly expressed in breast and leg muscle. In vitro, *ZFP36L2-AS* inhibits myoblast proliferation but promotes myoblast differentiation. In vivo, *ZFP36L2-AS* facilitates intramuscular fat deposition, as well as activates fast-twitch muscle phenotype and induces muscle atrophy. Mechanistically, *ZFP36L2-AS* interacts with acetyl-CoA carboxylase alpha (ACACA) and pyruvate carboxylase (PC) to induce ACACA dephosphorylation and damaged PC protein stability, thus modulating muscle metabolism. Meanwhile, *ZFP36L2-AS* can activate ACACA to reduce acetyl-CoA content, which enhances the inhibition of PC activity. Our findings present a novel model about the regulation of lncRNA on muscle metabolism.

## Introduction

As the largest tissue that comprises about 40% of the total body mass, skeletal muscle is a major player in regulating energy homeostasis and obesity progression [1–3]. A key metabolic feature of skeletal muscle is its plasticity, which is able to adjust fuel oxidation to fuel availability, called ‘metabolic flexibility’ [4, 5]. It’s well known that the metabolic regulation of skeletal muscle is pivotal for health and development, and loss of this flexibility is tightly associated with metabolic disorders such as obesity and muscle wasting [4, 6–8].

The maintenance of skeletal muscle mass is finely regulated by protein synthesis and catabolism [9]. Muscle atrophy refers to a decrease in muscle mass and fiber size and is characterized by enhanced protein degradation [10]. Muscle atrophy leading to muscle wasting seriously restricts animal development. Recently, muscle wasting has attracted many researchers’ attention, however, the molecular mechanisms that govern muscle atrophy remain largely unknown.

Protein-encoding genes only account for a small portion (2%) of the genome, and yet 70–90% of the genome is transcribed into long noncoding RNAs (lncRNAs) at some point during development [11]. LncRNAs are a new class of regulatory RNAs, commonly defined as transcribed RNAs of more than 200 nucleotides with low coding potential, are widely involved in gene expression regulation at the transcription, translation and epigenetic levels [12–16]. Although only a small number of functional lncRNAs have been well characterized to date, they seem to control major biological processes impacting skeletal muscle development and muscle disorders [17–20].

White recessive rock (WRR) is a hypertrophic broiler chicken with a fast growth rate, which exhibits a different growth performance from Xinghua (XH) chicken (a lean Chinese native breed with a slow growth rate) [21, 22]. In our previous RNA-seq study (accession number GSE58755), we found lncRNA TCONS_00067025 (named ZFP36 ring finger protein like 2 [*ZFP36L2*]-antisense transcript [*ZFP36L2-AS*]) differentially expressed between WRR (a fast growth rate broiler chicken) and XH chicken (a slow growth rate Chinese native breed) [22]. In the current study, functional studies demonstrated that *ZFP36L2-AS* inhibits myoblast proliferation but promotes myogenic differentiation in vitro. In vivo, *ZFP36L2-AS* represses fatty acid oxidation to facilitate intramuscular fat deposition, as well as activates fast-twitch muscle phenotype and induces muscle atrophy. Further mechanistic investigation revealed that *ZFP36L2-AS* interacts with acetyl-CoA carboxylase alpha (ACACA) and pyruvate carboxylase (PC) to induce ACACA activation and inhibit PC activity. Altogether, our studies uncover a functional lncRNA that modulates skeletal muscle development.

## Results

### *ZFP36L2-AS* is a novel lncRNA associated with skeletal muscle development

**Fig. 1:**
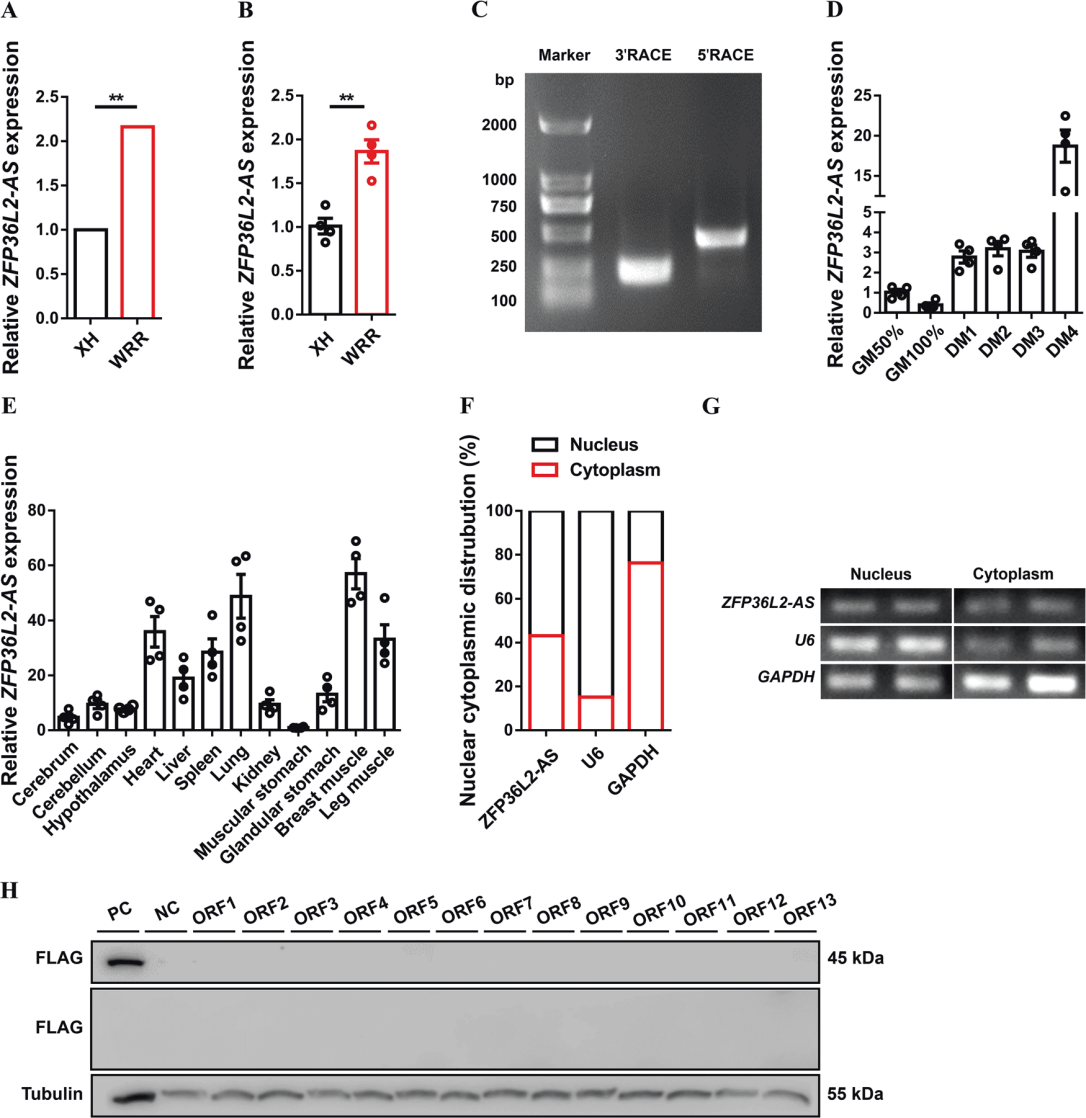
Identification of lncRNA *ZFP36L2-AS*. **A**, **B** Relative *ZFP36L2-AS* expression in breast muscles of 7-week-old Xinghua (XH) chicken and White recessive rock (WRR) detected by RNA-seq (**A**) and qPCR (**B**). (**C**) Results of *ZFP36L2-AS* 3’ RACE and 5’ RACE. **D** Relative *ZFP36L2-AS* expression during CPM proliferation and differentiation. **E** Tissue expression profiles of *ZFP36L2-AS*. The horizontal axis and vertical axis indicate different tissues and their relative expression values, respectively. **F**, **G** The distribution of *ZFP36L2-AS* in the cytoplasm and nuclei of chicken primary myoblast (CPM) was determined by qPCR (**F**) and semi-qPCR (**G**). *GAPDH* and *U6* serve as cytoplasmic and nuclear localization controls, respectively. **H** Western blot analysis of the coding ability of *ZFP36L2-AS*. The potential ORFs of *ZFP36L2-AS* were cloned into the pcDNA3.1-3xFlag-C vector. CPMs transfected with β-actin were used as a positive control (PC) and untransfected CPMs were used as a negative control (NC). Results are presented as mean ± SEM. In panels (**A**, **B**), statistical significance of differences between means was assessed using independent sample *t*-test. (*P < 0.05; **P < 0.01).

Our previous RNA-seq study found a novel lncRNA (*ZFP36L2-AS*) was highly expressed in fast growth rate broilers (Fig. 1A, B) [22], implied that *ZFP36L2-AS* is probably associated with skeletal muscle development. To obtain the full-length of *ZFP36L2-AS*, 5’ and 3’ ends of this lncRNA were determined by RACE system (Fig. 1C). The Basic Local Alignment Search Tool (BLAST) of the National Center for Biotechnology Information (NCBI) showed that *ZFP36L2-AS* was an antisense transcript of *ZFP36L2* with 3,465 nt long, located at chromosome 3 from 25,089,365 to 25,092,829, and mainly conserved in Aves (Supplementary Fig. 1A and Table 1). *ZFP36L2-AS* was upregulated during myogenic differentiation, and highly expressed in breast and leg muscle (Fig. 1D, E). Compared with other muscle-resident cells, *ZFP36L2-AS* was highly expressed in myoblasts (Supplementary Fig. 1B). Furthermore, cell-fractionation assays demonstrated that *ZFP36L2-AS* was present both in the cytoplasm and nucleus of chicken primary myoblast (CPM) (Fig. 1F, G). In order to verify the coding potential of *ZFP36L2-AS*, we further cloned 3xFLAG epitope tag in-frame with the C terminus of thirteen potential ORFs of *ZFP36L2-AS*. Crucially, western blot analysis indicated that *ZFP36L2-AS* was an lncRNA without protein-encoding potential (Fig. 1H).

### *ZFP36L2-AS* inhibits myoblast proliferation but promotes myogenic differentiation

**Fig. 2:**
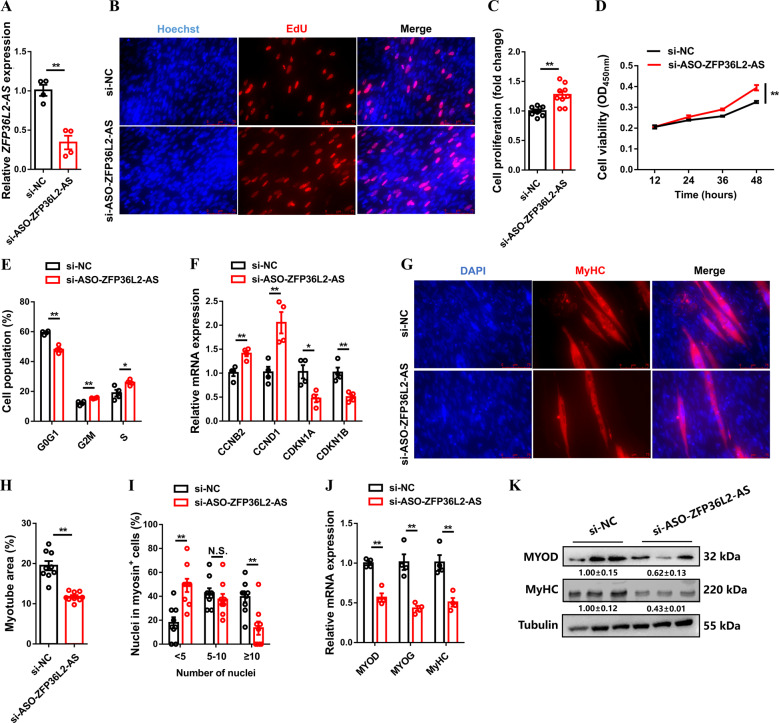
lncRNA *ZFP36L2-AS* inhibits myoblast proliferation but promotes myoblast differentiation. **A** Relative *ZFP36L2-AS* expression with *ZFP36L2-AS* interference in vitro. **B** Proliferation of transfected CPMs was assessed by 5-ethynyl-2’-deoxyuridine (EdU) incorporation. **C** Proliferation rate of myoblasts after interference of *ZFP36L2-AS*. **D** CCK-8 assays were performed in CPMs with *ZFP36L2-AS* interference. **E** Cell cycle analysis of myoblasts after interference of *ZFP36L2-AS*. **F** Relative mRNA levels of several cell cycle genes with *ZFP36L2-AS* interference. **G**–**I** MyHC immunostaining (**G**), myotube area (%) (**H**), and myoblast fusion index (**I**) of CPMs transduced with *ZFP36L2-AS* interference. Cells were differentiated for 72 h after transfection. The nuclei were visualized with 4′,6-diamidino-2phenylindole (DAPI). **J**, **K** Relative mRNA (**J**) and protein (**K**) expression levels of myoblast differentiation marker genes from si-ASO-ZFP36L2-AS transfected CPMs. The numbers shown below the bands were folds of band intensities relative to control. Band intensities were quantified by ImageJ and normalized to β-Tubulin. Data are expressed as a fold-change relative to the control. Results are shown as mean ± SEM. In panels (**A**, **C**–**F**, and **H**–**J**), statistical significance of differences between means was assessed using independent sample *t*-test. (*P < 0.05; **P < 0.01; N.S. no significant difference).

Given that *ZFP36L2-AS* was decreased in myoblast proliferation and upregulated during myogenic differentiation (Fig. 1D), we performed overexpression and inhibition experiments to assess its effect in proliferation and differentiation of myoblast (Fig. 2A and Supplementary Fig. 2A). The 5-ethynyl-2′-deoxyuridine (EdU) staining and cell counting kit-8 (CCK-8) assay demonstrated that *ZFP36L2-AS* interference significantly increased EdU incorporation and promoted myoblast proliferation, whereas *ZFP36L2-AS* overexpression significantly inhibited the proliferation of myoblast (Fig. 2B–D and Supplementary Fig. 2B–D). Flow cytometric analysis showed that *ZFP36L2-AS* inhibition significantly reduced the number of cells that progressed to G0/G1 and increased the number of S phase cells (Fig. 2E). Conversely, *ZFP36L2-AS* overexpression resulted in a larger number of G0/G1 and fewer S phase cells (Supplementary Fig. 2E). Furthermore, inhibition of *ZFP36L2-AS* increased the expression of cell cycle-promoting genes such as *CCNB2* and *CCND1*, while reduced cell cycle-inhibiting genes like *CDKN1A* and *CDKN1B* (Fig. 2F). And the opposite result was observed with *ZFP36L2-AS* overexpression (Supplementary Fig. 2F).

To further test whether *ZFP36L2-AS* functions in myogenic differentiation, immunofluorescence staining was performed after overexpression and inhibition of *ZFP36L2-AS*. *ZFP36L2-AS* inhibition significantly repressed myoblast differentiation and decreased the total areas of myotubes, while myotube formation was facilitated with *ZFP36L2-AS* overexpression (Fig. 2G–I and Supplementary Fig. 2G–I). In addition, the expressions level of myoblast differentiation marker genes, including *MYOD*, *MYOG*, and *MyHC* were significantly downregulated with *ZFP36L2-AS* interference (Fig. 2J, K). Conversely, overexpression of *ZFP36L2-AS* promoted their expression (Supplementary Fig. 2J, K).

### *ZFP36L2-AS* decreases cellular respiration, fatty acid oxidation, and TCA cycle metabolites in skeletal muscle

**Fig. 3:**
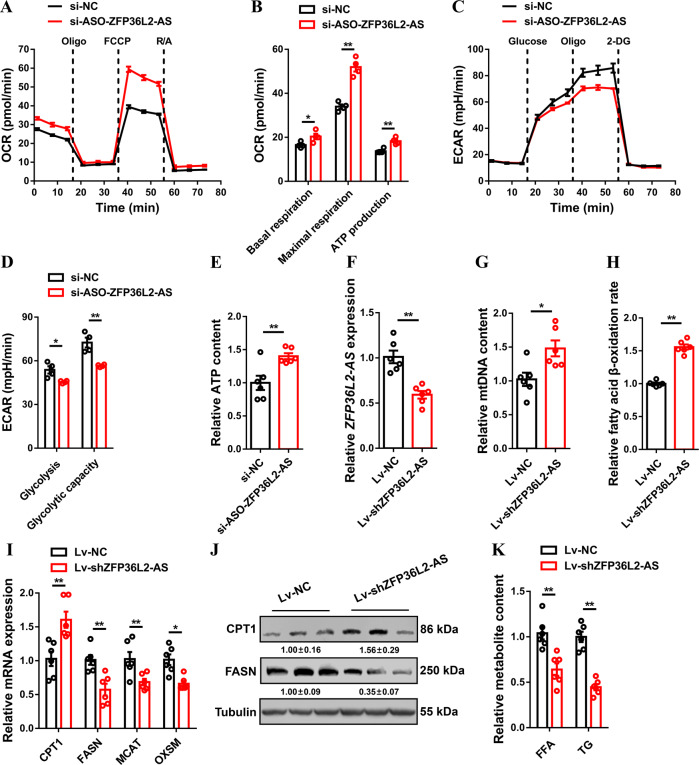
lncRNA *ZFP36L2-AS* represses cellular respiration and fatty acid oxidation in skeletal muscle. **A**, **B** Oxygen consumption rate (OCR) (**A**), and basal respiration, maximal respiration, and ATP production (**B**) of myoblasts after interference of *ZFP36L2-AS*. (**C**, **D**) Extracellular acidification rate (ECAR) (**C**), and glycolysis and glycolytic capacity (**D**) of myoblasts with *ZFP36L2-AS* interference. **E** Relative cellular adenosine triphosphate (ATP) content with *ZFP36L2-AS* interference in CPMs. **F** Relative *ZFP36L2-AS* expression in gastrocnemius after infected with lentivirus-mediated *ZFP36L2-AS* knockdown (Lv-shZFP36L2-AS) or negative control (Lv-NC). **G** Relative mitochondrial DNA (mtDNA) content in *ZFP36L2-AS* knockdown gastrocnemius. (**H**) Relative fatty acid β-oxidation rate in gastrocnemius with *ZFP36L2-AS* knockdown. **I**, **J** Relative mRNA (**I**) and protein (**J**) expression levels of fatty acid oxidation or synthesis related-genes after infected with the indicated lentivirus. The numbers shown below the bands were folds of band intensities relative to control. Band intensities were quantified by ImageJ and normalized to β-Tubulin. Data are expressed as a fold-change relative to the control. **K** Relative free fatty acid (FFA) and triglyceride (TG) content in gastrocnemius with *ZFP36L2-AS* knockdown. Results are presented as mean ± SEM. In panels (**B**, **D**–**I**, and **K**), statistical significance of differences between means was assessed using independent sample *t*-test. (*P < 0.05; **P < 0.01).

Cellular mitochondrial activities including oxygen consumption rate (OCR), basal and maximal mitochondrial respiration, and adenosine triphosphate (ATP) production were elevated with *ZFP36L2-AS* interference, whereas *ZFP36L2-AS* overexpression facilitated glycolysis (Fig. 3A–D and Supplementary Fig. 3A–D), indicating that *ZFP36L2-AS* may be involved in cellular energy metabolism. Meanwhile, inhibition of *ZFP36L2-AS* increased ATP content in myoblast, while ATP content was decreased with *ZFP36L2-AS* overexpression in myoblasts (Fig. 3E and Supplementary Fig. 3E). Given that *ZFP36L2-AS* regulates cellular metabolism, we further detected the cellular ATP content in satellite cells after inhibition and overexpression of *ZFP36L2-AS* to explore whether *ZFP36L2-AS* plays a metabolic regulatory role in satellite cells similar to that in myoblasts. The results shown that *ZFP36L2-AS* did not regulate cellular ATP content in satellite cells (Supplementary Fig. 4A, B), suggesting the function of *ZFP36L2-AS* in muscle metabolism mainly depends on its expression in myoblasts. To investigate the potential roles of *ZFP36L2-AS* in vivo, the gastrocnemius of 1day-old chick was injected with adenovirus-mediated *ZFP36L2-AS* overexpression (Adv-ZFP36L2-AS) or lentivirus-mediated *ZFP36L2-AS* knockdown (Lv-shZFP36L2-AS) (Fig. 3F and Supplementary Fig. 3F). *ZFP36L2-AS* knockdown increased mitochondrial DNA content, which was potentially contribute to the acceleration of fatty acid oxidation (FAO) (Fig. 3G, H). In contrast, mitochondrial DNA content and fatty acid β-oxidation were reduced after overexpression of *ZFP36L2-AS* (Supplementary Fig. 3G, H). Besides, knockdown of *ZFP36L2-AS* upregulated the expression of FAO-related gene like *CPT1*, but downregulated key genes involved in fatty acid synthesis (such as *FASN*, *MCAT* and *OXSM*) and reduced intramuscular free fatty acid (FFA) and triglyceride (TG) content (Fig. 3I–K). Meanwhile, opposite results were showed with *ZFP36L2-AS* overexpression (Supplementary Fig. 3I–K).

**Fig. 4:**
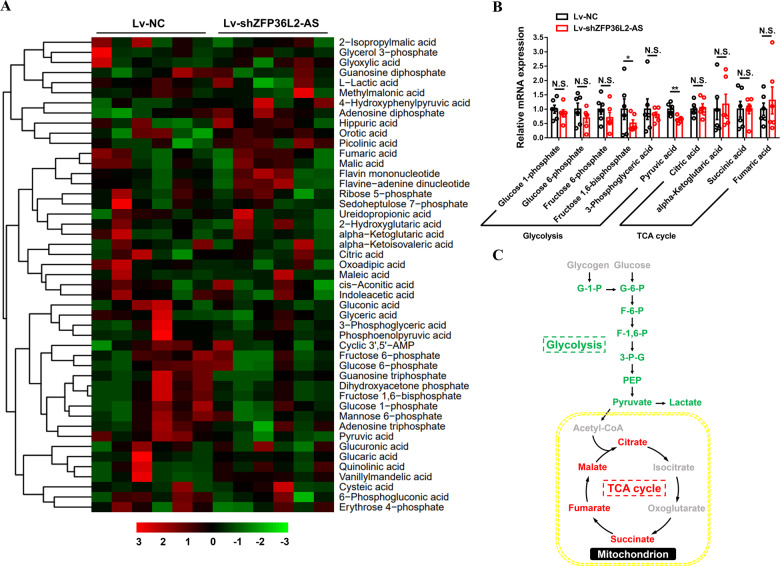
Knockdown of lncRNA *ZFP36L2-AS* downregulates the TCA cycle. **A** Hierarchical clustering analysis (HCA) of metabolites in gastrocnemius after infected with Lv-shZFP36L2-AS or Lv-NC. The colors indicate the relative levels in lncRNA *ZFP36L2-AS* knockdown or control group. **B** Relative metabolite content of glycolysis and tricarboxylic acid (TCA) cycle in gastrocnemius with *ZFP36L2-AS* knockdown. **C** Schematic diagram of metabolic pathways of glycolysis and TCA cycle affected by *ZFP36L2-AS* knockdown in the gastrocnemius. Upregulated metabolites are shown in red, and the downregulated metabolites are shown in green. In panel (**C**), results are shown as mean ± SEM, statistical significance of differences between means was assessed using independent sample *t*-test. (*P < 0.05; **P < 0.01).

Excessive lipid storage is often accompanied by changes in muscle metabolism [23–26]. To further study the regulation of *ZFP36L2-AS* on muscle metabolism, a comparative metabolome analysis was performed with *ZFP36L2-AS* knockdown in gastrocnemius. Hierarchical clustering analysis (HCA) based on metabolite levels showed that several tricarboxylic acid cycle (TCA cycle) metabolites actually accumulate in *ZFP36L2-AS* knockdown gastrocnemius (Fig. 4A, B and Supplementary Table 2). On the contrary, *ZFP36L2-AS* knockdown significantly reduced glycolytic metabolites such as Fructose 1,6-bisphosphate and Dihydroxyacetone phosphate (Fig. 4A, B and Supplementary Table 2). Altogether, our results indicated that *ZFP36L2-AS* impairs mitochondrial respiration and FAO, leading to the accumulation of lipid metabolites, which elevates glycolysis as compensatory responses (Fig. 4C).

### *ZFP36L2-AS* activates a fast-twitch gene expression profile concurrent with muscle atrophy

**Fig. 5:**
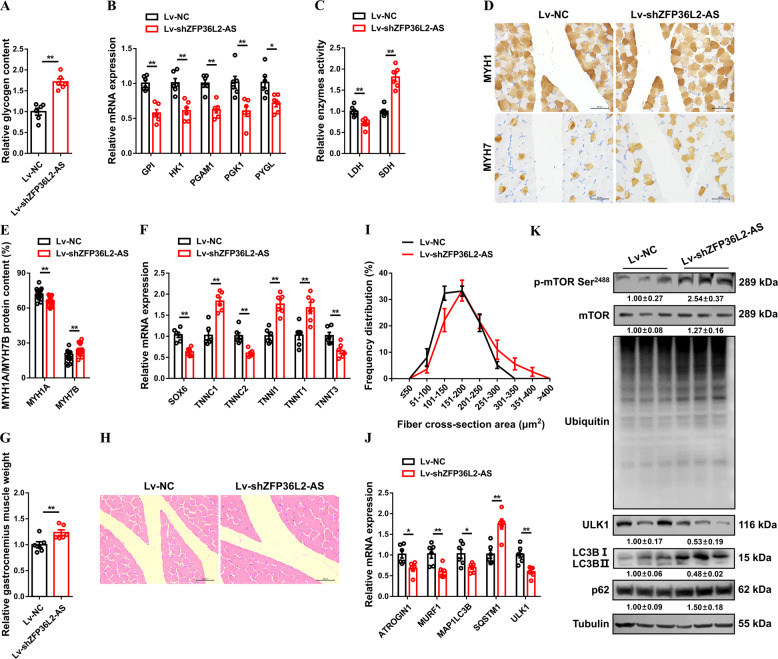
lncRNA *ZFP36L2-AS* activates a fast-twitch gene expression profile concurrent with muscle atrophy. **A** Relative glycogen content in gastrocnemius with *ZFP36L2-AS* knockdown. **B** Relative mRNA expression levels of glycogenolytic and glycolytic genes in gastrocnemius after infected with the indicated lentivirus. (**C**) Relative enzymes activity of lactic dehydrogenase (LDH) and succinate dehydrogenase (SDH) in gastrocnemius infected with *ZFP36L2-AS* knockdown. **D**, **E** Immunohistochemistry analysis of MYH1/MYH7 (**D**) and MYH1/MYH7 protein content (**E**) of gastrocnemius after *ZFP36L2-AS* knockdown. (**F**) Relative mRNA expression levels of several fast-/slow-twitch myofiber genes with *ZFP36L2-AS* knockdown. **G** Relative gastrocnemius muscle weight after infected with the indicated lentivirus. **H**, **I** H&E staining (**H**) and frequency distribution of fiber cross-section area (CSA) (**I**) of transverse sections of gastrocnemius with *ZFP36L2-AS* knockdown. **J** Relative mRNA expression of the atrophy and autophagy-related genes in gastrocnemius after infected with the indicated lentivirus. **K** The protein expression levels of mTOR signaling after *ZFP36L2-AS* knockdown. The numbers shown below the bands were folds of band intensities relative to control. Band intensities were quantified by ImageJ and normalized to β-Tubulin. Data are expressed as a fold-change relative to the control. Results are presented as mean ± SEM. In panels (**A**–**C**, **E**–**G**, and **J**), statistical significance of differences between means was assessed using independent sample *t*-test. (*P < 0.05; **P < 0.01).

Skeletal muscle is comprised of heterogeneous myofibers that differ in their physiological and metabolic parameters [27]. Compared with fast-twitch (type II; glycolytic) myofibers, slow-twitch (type I; oxidative) myofibers have more myoglobin, more mitochondria, and higher activity of oxidative metabolic enzymes [28, 29]. In response to environmental demands, skeletal muscle can remodel by activating signaling pathways to reprogram gene expression to sustain muscle performance [27]. Given that knockdown of *ZFP36L2-AS* reduced the accumulation of glycolytic metabolites and upregulated oxidative metabolism in gastrocnemius (Fig. 4B, C), *ZFP36L2-AS* may function in the transformation of myofiber type by modulating muscle metabolism. As expected, glycogen content was increased and expression of glycogenolytic and glycolytic genes was downregulated with *ZFP36L2-AS* knockdown (Fig. 5A, B). Inversely, overexpression of *ZFP36L2-AS* reduced the accumulation of glycogen, as well as promoted expression of glycogenolytic and glycolytic genes (Supplementary Fig. 5A, B). *ZFP36L2-AS* knockdown suppressed the activity of lactic dehydrogenase (LDH) and enhanced the activity of succinate dehydrogenase (SDH), whereas *ZFP36L2-AS* overexpression elevated glycolytic capacity and decrease oxidative capacity of skeletal muscle (Fig. 5C and Supplementary Fig. 5C). More importantly, immunohistochemical results showed that *ZFP36L2-AS* knockdown suppressed MYH1A/fast-twitch protein level and promoted the expression level of MYH7B/slow-twitch protein (Fig. 5D, E). The expressions of multiple fast-twitch myofiber genes such as *SOX6*, *TNNC2* and *TNNT3* were significantly promoted, while slow-twitch myofiber genes like *TNNC1*, *TNNI1* and *TNNT1* were inhibited with *ZFP36L2-AS* knockdown (Fig. 5F). On the contrary, *ZFP36L2-AS* overexpression upregulated fast-twitch protein level and expression of fast-twitch myofiber genes, drove the transformation of slow-twitch to fast-twitch myofibers (Supplementary Fig. 5D–F).

Muscle remodeling can also affect muscle mass; this is regulated by anabolic and catabolic signaling pathways, which induce muscle hypertrophy and muscle atrophy, respectively [30]. Here, *ZFP36L2-AS* knockdown increased muscle mass and elevated the proportion of large myofiber (> 200 μm^2^) (Fig. 5G–I). Conversely, gastrocnemius mass was reduced and proportion of small myofiber (< 200 μm^2^) was increased with overexpression of *ZFP36L2-AS* (Supplementary Fig. 5G–I), suggesting *ZFP36L2-AS* is involved in muscle atrophy. Mammalian target of rapamycin (mTOR) is a master growth regulator that senses and integrates diverse nutritional and environmental cues, can resist muscle atrophy by inhibiting proteasomal degradation and autophagy [30–32]. To further explore the regulatory mechanism of *ZFP36L2-AS* on muscle atrophy, we assessed the mTOR signaling after *ZFP36L2-AS* overexpression and knockdown. *ZFP36L2-AS* knockdown facilitated Ser^2488^ phosphorylation of mTOR, thus inactivating ubiquitin-proteasome system (UPS) and autophagy-lysosomal system, while the mTOR signaling was inhibited with *ZFP36L2-AS* overexpression (Fig. 5J, K and Supplementary Fig. 5J, K), indicating that *ZFP36L2-AS* induces muscle atrophy by inhibiting the mTOR signaling.

### *ZFP36L2-AS* interacts with ACACA and PC

**Fig. 6:**
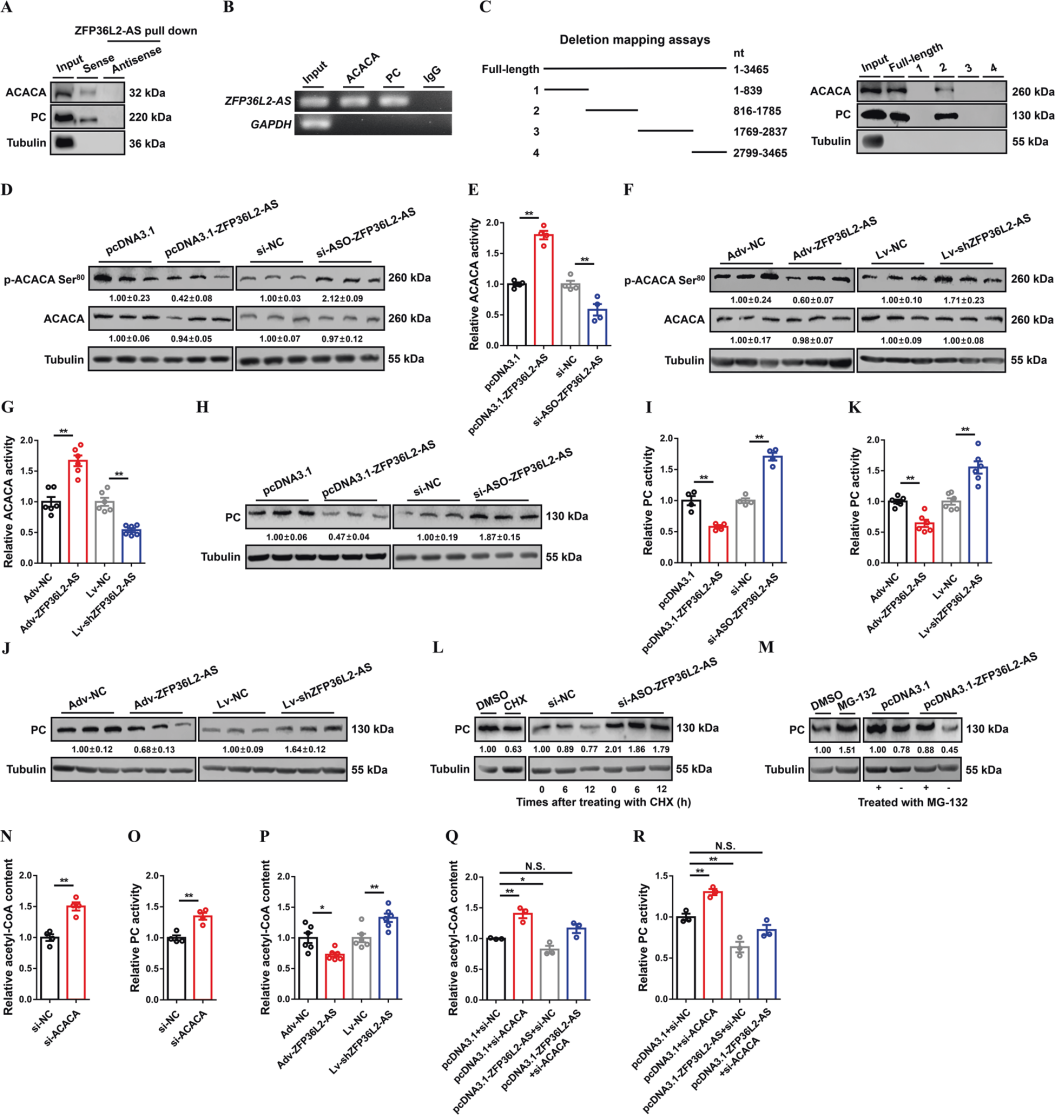
lncRNA *ZFP36L2-AS* interacts with ACACA and PC to induce ACACA dephosphorylation and inhibit PC protein stabilization. **A**, **B** lncRNA *ZFP36L2-AS* interacts with ACACA and PC protein were determined by biotin-labeled RNA pulldown (**A**) and RIP (**B**). **C** The interaction of full-length and truncated *ZFP36L2-AS* with ACACA and PC protein was determined by RNA pulldown. **D–G** The protein expression levels of ACACA and phosphorylated ACACA (**D**, **F**), and relative ACACA activity (**E** and **G**) after *ZFP36L2-AS* overexpression or knockdown in vitro and in vivo. **H**–**K** The protein expression level of PC (**H**, **J**), and relative PC activity (**I**, **K**) after *ZFP36L2-AS* overexpression or knockdown in vitro and in vivo. (**L**) The protein expression level of PC in *ZFP36L2-AS* overexpressed myoblast was analyzed after incubated with the protein synthesis inhibitor cycloheximide (CHX; 25 µg/ml). (**M**) The protein expression level of PC in *ZFP36L2-AS* knockdowned myoblast was analyzed after incubated with the proteasome inhibitor (MG-132; 5 µmol/L) for 12 h. **N**, **O** Relative acetyl-CoA content (**N**), and relative PC activity (**O**) with *ACACA* interference in vitro. **P** Relative acetyl-CoA content with *ZFP36L2-AS* overexpression or knockdown in vivo. **Q**, **R** Relative acetyl-CoA content (**Q**), and relative PC activity (**R**) induced by the listed nucleic acids. In panels (**D**, **F**, **H**, **J**, **L**, and **M**), the numbers shown below the bands were folds of band intensities relative to control. Band intensities were quantified by ImageJ and normalized to β-Tubulin. Data are expressed as a fold-change relative to the control. Results are presented as mean ± SEM. In panels (**E**, **G**, **I**, **K** and **N**–**P**), statistical significance of differences between means was assessed using independent sample *t*-test. In panels (**Q**, **R**), ANOVA followed by Dunnett’s test was used. (*P < 0.05; **P < 0.01; N.S., no significant difference).

Molecular decoy is one of the main molecular mechanisms for lncRNA to function. It refers to that lncRNA directly binds to RNA or protein molecules, thereby activating or blocking the role and signal pathway of these molecule [33]. To elucidate the mechanism by which *ZFP36L2-AS* regulates skeletal muscle development, we attempted to identify its endogenous binding proteins by performing RNA pull-down coupled to mass spectrometry. Compared with the *ZFP36L2-AS* antisense group, a total of 141 proteins (protein score ≥ 19) were identified specifically bind to *ZFP36L2-AS* sense transcript (Supplementary Table 3). Gene ontology (GO) and Kyoto Encyclopedia of Genes and Genomes (KEGG) enrichment analysis found that these RNA binding proteins (RBPs) were mainly enriched in biological processes such as cellular process, metabolic process, cellular component organization or biogenesis, and biological regulation, as well as participated in biological processes including metabolic pathways, carbon metabolism, TCA cycle, pyruvate metabolism, glycolysis/gluconeogenesis and so on (Supplementary Fig. 6A, B). Acetyl-CoA carboxylase alpha (ACACA) and pyruvate carboxylase (PC) are members of biotin-dependent carboxylase, which are known to be widely involved in metabolic regulation [34–37], were found specifically bind to *ZFP36L2-AS* (Supplementary Table 3). To corroborate this result, we performed western blot analysis of RNA pull-down samples, which validated the interaction of *ZFP36L2-AS* with ACACA and PC protein (Fig. 6A). Moreover, the specificity of these interactions was also verified with RNA immunoprecipitation (RIP) (Fig. 6B).

To further map the *ZFP36L2-AS* functional motifs corresponding to ACACA and PC binding, we conducted an in vitro RNA pull-down assay using a series of truncated *ZFP36L2-AS* fragments (Fig. 6C). This analysis revealed that nucleotides 816-1785 of *ZFP36L2-AS* are sufficient to interact with both ACACA and PC protein, while other *ZFP36L2-AS* truncated fragments could not (Fig. 6C). As the *ZFP36L2-AS* 816-1785 region is necessary for *ZFP36L2-AS*’s binding to ACACA and PC protein, we overexpressed the truncated fragment (816-1785 nt) and analyzed its impact on skeletal muscle development. Similar results to *ZFP36L2-AS* full-length overexpression were found (Supplementary Fig. 7A–F), implying that the interaction of *ZFP36L2-AS* with ACACA and PC protein may be a requisite for *ZFP36L2-AS* to function.

### The function of *ZFP36L2-AS* partially depends on its regulation of ACACA and PC activity

With the observation that *ZFP36L2-AS* directly interacts with ACACA and PC, we further analyzed the effect of *ZFP36L2-AS* on ACACA and PC. Both in vitro and in vivo, the mRNA level of *ACACA* and *PC* were not changed with *ZFP36L2-AS* overexpression and knockdown (Supplementary Fig. 8A–D). Overexpression of *ZFP36L2-AS* significantly inhibited the phosphorylation level of ACACA protein and increased ACACA activity, whereas *ZFP36L2-AS* knockdown promoted ACACA phosphorylation and inactivated ACACA (Fig. 6D–G and Supplementary Fig. 7G, H), indicating that *ZFP36L2-AS* modulates the activity of ACACA by regulating ACACA phosphorylation. Next, we investigated the regulation of *ZFP36L2-AS* on the protein level and activity of PC. *ZFP36L2-AS* overexpression downregulated PC protein level and activity, while the protein level and activity of PC was increased with *ZFP36L2-AS* knockdown (Fig. 6H–K and Supplementary Fig. 7I, J). Cycloheximide (CHX) is a bacterial toxin that can inhibit protein biosynthesis. Treated with CHX decreased the expression of PC protein (Fig. 6L). However, *ZFP36L2-AS* knockdown relieved the decline of PC protein expression induced by CHX (Fig. 6L), suggesting that *ZFP36L2-AS* might promote the PC protein degradation. To further clarify the possible mechanism, the proteasome inhibitor MG-132 was used. MG-132 upregulated the protein levels of PC (Fig. 6M), suggesting that the inhibition of ubiquitin-proteasome pathway might ameliorate the degradation of PC. More importantly, MG-132 rescued the reduction of PC protein levels in *ZFP36L2-AS*-overexpressing myoblast (Fig. 6M). Overall, given that *ZFP36L2-AS* could modulate the ubiquitination level of total protein (Fig. 5K and Supplementary Fig. 5K), we infer *ZFP36L2-AS* induces the ubiquitination of PC to facilitate PC degradation, thereby inhibiting PC activity.

Acetyl-CoA is an allosteric activator of PC [38], has been found that can be catalyzed by ACACA to produce malonyl-CoA for fatty acid synthesis [39, 40], hinting that ACACA may affect the activity of PC by regulating acetyl-CoA. To verify this conjecture, *ACACA* was knockdown by specific siRNA (Supplementary Fig. 9A, B). As expected, *ACACA* knockdown increased acetyl-CoA content and enhanced the activity of PC (Fig. 6N, O). Similarly, *ZFP36L2-AS* knockdown augmented acetyl-CoA content, whereas the content of acetyl-CoA was reduced with overexpression of *ZFP36L2-AS* (Fig. 6P and Supplementary Fig. 7K). To further explore whether *ZFP36L2-AS* can reinforce the regulation of PC activity *via ACACA*, *ZFP36L2-AS* overexpression construct was co-transfected with *ACACA* specific siRNA. Partly, knockdown of *ACACA* abolished the downregulation of acetyl-CoA content and PC activity by *ZFP36L2-AS* overexpression (Fig. 6Q, R), demonstrated that *ZFP36L2-AS* can further enhance the inhibition of PC activity by activating ACACA.

Tissue expression profiles showed *ACACA* and *PC* highly expressed in breast and leg muscle (Supplementary Fig. 10A, D), implying that they may play an important role in skeletal muscle development. We further analyzed the expression of *ACACA* and *PC* during myoblast proliferation and differentiation. *ACACA* was slightly upregulated during myogenic differentiation, whereas the expression of *PC* was visibly decreased (Supplementary Fig. 10B, E). Moreover, subcellular location annotation showed that ACACA protein exists in nucleus and cytosol, while PC is localized in mitochondria (Supplementary Fig. 10C, F). To explore the potential biological functions of *ACACA* and *PC* in myogenesis, we performed a series of myoblast proliferation and differentiation assays. *ACACA* knockdown had similar results with *PC* overexpression, which promoted myoblast proliferation and inhibited myoblast differentiation (Supplementary Figs. 9C–L and 11A–L). In contrast, *PC* interference suppressed myoblast proliferation but facilitated myogenic differentiation (Supplementary Fig. 11M-X). Meanwhile, overexpression of *PC* promoted cellular mitochondrial respiration, whereas glycolytic capacity was increased with *PC* knockdown (Supplementary Fig. 12A-H).

**Fig. 7:**
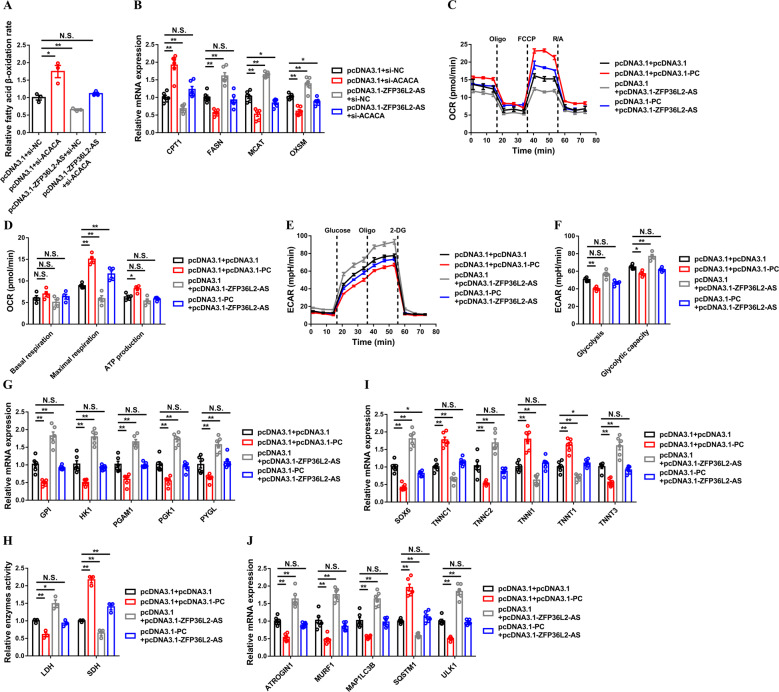
ACACA and PC are required for the function of lncRNA *ZFP36L2-AS*. **A**, **B**) Relative fatty acid β-oxidation rate (**A**), and relative mRNA levels of fatty acid oxidation or synthesis related-genes (**B**) after co-transfection with the listed nucleic acids in CPMs. **C**–**J** OCR (**C**), basal respiration, maximal respiration and ATP production (**D**), ECAR (**E**), glycolysis and glycolytic capacity (**F**), relative mRNA expression levels of glycogenolytic and glycolytic genes (**G**), relative enzymes activity of LDH and SDH (**H**), relative mRNA expression levels of several fast-/slow-twitch myofiber genes (**I**), and relative mRNA expression of the atrophy and autophagy-related genes (**J**) induced by the listed nucleic acids in CPMs. Results are shown as mean ± SEM. In all panels, statistical significance of differences between means was assessed using ANOVA followed by Dunnett’s test. (*P < 0.05; **P < 0.01; N.S. no significant difference).

Considering the expression and functional relationship between *ZFP36L2-AS* and *ACACA* and *PC*, we further determined the role of *ACACA* and *PC* on *ZFP36L2-AS*-mediated skeletal muscle development. Knockdown of *ACACA* improved fatty acid β-oxidation rate and upregulated the expression of *CPT1*, but downregulated key genes involved in fatty acid synthesis, which counteracts the inhibitory effect of *ZFP36L2-AS* on FAO (Fig. 7A, B). In addition, overexpression of *PC* rescued the suppression of cellular mitochondrial respiration induced by *ZFP36L2-AS* overexpression (Fig. 7C–F), as well as attenuates the activation of fast-twitch phenotype and muscle atrophy (Fig. 7G–J). Altogether, these results indicated that ACACA and PC are indispensable to the function of *ZFP36L2-AS*.

## Discussion

Myogenesis is a highly ordered process including myoblast proliferation and differentiation, myotube formation and maturity, and is controlled by a series of myogenic regulatory factors [30, 41–43]. After birth, the number of myofibers in animals is basically fixed, and their skeletal muscle development is mainly regulated by the composition and size of myofibers. Recently, it is becoming increasingly clear that a complex network of epigenetic regulators and lncRNAs plays an essential role in skeletal muscle development [17–20, 44]. In our previous RNA-seq data, we identified lncRNA *ZFP36L2-AS* was highly expressed in fast growth rate broilers. Here, we found *ZFP36L2-AS* was upregulated during myogenic differentiation and highly expressed in breast and leg muscle, indicating its potential role in muscle development. Gain- and loss-of-function analysis revealed that *ZFP36L2-AS* inhibited myoblast proliferation but promoted myoblast differentiation in vitro. In vivo, *ZFP36L2-AS* activated fast-twitch muscle phenotype and induced muscle atrophy.

Skeletal muscle is a structurally and metabolically plastic tissue that maintains systemic energy homeostasis in response to various metabolic stresses [26]. Metabolic inflexibility in muscles is a dominant cause of various metabolic disorders [5]. Notably, recent evidences have revealed that the maintenance of skeletal muscle mass is closely related to muscle metabolism [8]. In this study, we found that *ZFP36L2-AS* repressed cellular mitochondrial respiration and fatty acid oxidation in skeletal muscle, resulting in excessive deposition of intramuscular fat. In the meantime, *ZFP36L2-AS* elevated glycolytic capacity and decrease oxidative capacity of skeletal muscle, which inactivated mTOR signaling, leading to the activation of UPS and autophagy-lysosomal system and induced muscle atrophy. Given that *ZFP36L2-AS* facilitated intramuscular fat deposition and induced muscle atrophy, *ZFP36L2-AS* could be a novel therapeutic target for obesity and sarcopenia.

Post-transcriptional regulation is an important form for lncRNA to regulate gene expression and function. Notably, increasing studies revealed that lncRNAs can be widely involved in a variety of biological processes through interacting with RBPs [44–47]. ACACA is a key enzyme in the process of fatty acid biosynthesis and oxidation, whose Ser79 site phosphorylation would inhibit the enzymatic activity of ACACA to promote FAO [48, 49]. In this study, we found *ZFP36L2-AS* can interact with ACACA through its 816-1785 region, thus inducing ACACA dephosphorylation and facilitating intramuscular fat deposition. In addition, PC (an enzyme that converts pyruvate to oxaloacetate), who has been reported to function as a RBP [45], was also discovered to bind with *ZFP36L2-AS*. *ZFP36L2-AS* damaged PC protein stability and inhibited PC activity, which may be attributed to the induction of ubiquitination by *ZFP36L2-AS*. Interestingly, we found ACACA can reduce the activity of PC by consuming acetyl-CoA, demonstrating that the inhibitory effect of *ZFP36L2* on PC activity is partially ACACA-dependent.

Skeletal muscle is mainly composed of myofibers, which develop from myoblasts through a highly ordered biological process. In addition to myoblasts, skeletal muscle includes many muscle-resident cells such as blood cells, fibroblasts, preadipocytes, and satellite cells. Compared to other muscle-resident cells, we found that *ZFP36L2-AS* expression is more pronounced in myoblasts, blood cells and satellite cells. Since blood cells are mainly involved in oxygen transport and immune function of the body and *ZFP36L2-AS* regulates skeletal muscle development by mediating muscle metabolism, the function of *ZFP36L2-AS* in blood cells was not investigated in depth. On the other hand, inhibition and overexpression of *ZFP36L2-AS* did not change cellular ATP content in satellite cells, suggesting the expression of *ZFP36L2-AS* in satellite cells is not related to its role in muscle metabolism. Overall, the function of *ZFP36L2-AS* in muscle metabolism mainly depends on its expression in myoblasts, which contributes to its regulation of myogenesis and skeletal muscle development.

In summary, we identified lncRNA *ZFP36L2-AS* can interact with ACACA and PC to facilitate intramuscular fat deposition, as well as activate fast-twitch muscle phenotype and induce muscle atrophy (Supplementary Fig. 13). Our findings present a novel model about the regulation of lncRNA on muscle metabolism, and will contribute to the development of further research.

## Materials and methods

### Cell culture and transfection

Chicken primary myoblasts (CPMs) were isolated from the leg muscle of 11-day old chicken embryos and cultured as previously described [50]. To induce myogenic differentiation, growth medium was removed and replaced with differentiation medium (RPMI-1640 medium [Gibco, MD, USA] containing 2% horse serum after myoblasts achieving 90% cell confluence.

Fresh blood was collected from 14-day old chicken. After centrifugation at 1,500 × g, plasma was removed and blood cells were collected.

Chicken primary fibroblasts were isolated from the leg muscle of 10-day old chicken embryos. Fibroblasts were trypsinized and then collected by centrifugation as previously described [51]. Fibroblasts were cultured in Dulbecco’s modified Eagle’s medium (DMEM, Gibco, USA) supplemented with 10% (v/v) fetal bovine serum (FBS, Hyclone, USA) and 0.2% penicillin/streptomycin (Invitrogen, USA).

Chicken preadipocytes were isolated from 14-day old chicken as previously described [52], and cultured with DMEM/Ham’s nutrient mixture F-12 (DMEM/F12) basic medium with 10% (v/v) FBS (Hyclone, USA) and 0.2% penicillin/streptomycin (Invitrogen, USA).

Chicken satellite cells were isolated from leg muscle of 15-day old chicken embryos and cultured as previously described [53].

All cells were cultured at 37 °C in a 5% CO_2_ humidified atmosphere. And all transient transfections were performed using Lipofectamine 3000 Reagent (Invitrogen, USA) according to the manufacturer’s instructions.

### RNA extraction, cDNA synthesis and quantitative real-time PCR (qRT-PCR)

Total RNA was extracted using Trizol reagent (TaKaRa, Otsu, Japan) following the manufacturer’s protocol. Nuclear and cytoplasmic RNA fractionation was performed by using the PARIS Kit (Ambion, Life Technologies, USA) as recommended by the supplier. cDNA synthesis for mRNA was carried out using the PrimeScript RT Reagent Kit with gDNA Eraser (Perfect Real Time) (TaKaRa, Otsu, Japan). Real-time qPCR assay was performed as described before [54]. And primers used for RT-PCR and qRT-PCR are listed in Supplementary Table 4.

### 5ʹ and 3ʹ rapid-amplification of cDNA ends (RACE)

5′ and 3′ RACE of *ZFP36L2-AS* was performed using SMARTer RACE cDNA Amplification Kit (Clontech, Osaka, Japan) according to the manufacturer’s instructions. The gene-special primers used for RACE were presented in Supplementary Table 4.

### Plasmids construction and RNA oligonucleotides

For Flag fusion protein construction, thirteen ORFs of *ZFP36L2-AS* were amplified and subcloned into *HindIII* and *XhoI* restriction sites in the pcDNA3.1-3xFlag-C vector.

For overexpression vectors construction, the full-length sequence and 816-1785 nt of *ZFP36L2-AS* and *PC* coding sequence (NCBI Reference Sequence: NM_204346.1) were amplified and cloned into the pcDNA-3.1 vector (Promega, Madison, WI, USA) by using the *NheI* and *HindIII* restriction sites.

For viral vectors constructed, the full-length sequence of *ZFP36L2-AS* was amplified, and then cloned into the adenoviral vector (pDC316-mCMV-ZsGreen; Addgene, Cambridge, MA, USA) between *NheI* and *HindIII* sites. Short hairpin RNA (shRNA) against *ZFP36L2-AS* was designed by Shanghai Hanbio Biotechnology Co., Ltd, and then subcloned into the pLVX-shRNA2-Puro vector (Addgene, Cambridge, MA, USA) by using the *BamHI* and *EcoRI* restriction sites.

The small interfering RNAs (siRNA) and antisense oligonucleotide (ASO) that were used for the specific knockdown of *ZFP36L2-AS* were designed and synthesized by Guangzhou RiboBio (Guangzhou, China). The siRNA against *ACACA* (NCBI Reference Sequence: NM_205505.1) and *PC* were also designed and synthesized.

The primers and oligonucleotide sequences used in this study are shown in Supplementary Tables 4 and 5.

### Flow cytometry, 5-Ethynyl-2’-deoxyuridine (EdU) and cell counting kit-8 (CCK-8) assays

The experiments were performed as previously described [54]. In brief, the Cell Cycle Analysis Kit (Thermo Fisher Scientific, USA), C10310 EdU Apollo In Vitro Imaging Kit (RiboBio, China) and TransDetect Cell Counting Kit (TransGen, Beijing, China) were used for flow cytometry, EdU, and CCK-8 assay, as the manufacturer’s protocol.

### Immunoblotting and immunofluorescence (IF)

Western blots were performed as previously described [50]. The primary antibodies used were anti-FLAG (AF519, 1:1,000, Beyotime), anti-ACACA (PA5-17564, 1:1000, Thermo Fisher Scientific), anti-p-ACACA Ser^80^ (orb315750, 1:500, Biorbyt), anti-PC (GTX132002, 1:500, GeneTex), anti-MYOD (ABP53067, 1:500, Abbkine), anti-MyHC (B103, 0.5 μg/ml, DHSB), anti-CPT1 (bs-23779R, 1:500, Bioss), anti-FASN (10624-2-AP, 1:200, Proteintech), anti-p-mTOR Ser^2488^ (#5536, 1:1000, CST), anti-mTOR (bs-1992R, 1:500; Bioss), Ubiquitin (#39361:1000, CST) anti-ULK1 (bs-3602R, 1:500; Bioss), anti-LC3B (NB100-2220, 2.0 ug/ml, Novus), anti-P62 (18420-1-AP, 1:1000, Proteintech) and anti-β-Tubulin (A01030, 1:10000, Abbkine). ProteinFind Goat Anti-Mouse IgG(H + L), HRP Conjugate (HS201-01, 1:1,000, TransGen) and ProteinFind Goat Anti-Rabbit IgG(H + L), HRP Conjugate (HS101-01, 1:500, TransGen) were used as a secondary antibody.

Immunofluorescence was performed using anti-MyHC (B103, 2.5 μg/ml, DHSB), as previously described [50]. A fluorescence microscope ((DMi8; Leica, German) was used to capture three randomly selected fields to visualize the area labeled with anti-MyHC.

### Mitochondrial respiration assay

The oxygen consumption rate (OCR) and extracellular acidification rate (ECAR) of transfected myoblasts were measured using Seahorse XF Cell Mito Stress Test Kit and Seahorse XF Glycolysis Stress Test Kit (Agilent technologies, CA, USA) by a Seahorse XF96 Extracellular Flux Analyzer (Agilent technologies, CA, USA) following the manufacturer’s protocol, respectively.

### Adenovirus/Lentivirus production and transduction

To generate adenovirus, the recombinant adentiviral expression plasmid was co-transfected with pHBAd-BHGlox ΔE1,3Cre plasmid using Lipofectamine 3000 reagent. After amplification, acquired adenovirus were purified with a ViraBind™ Adenovirus Purification Kit (Cell Biolabs, USA). Lentivirus production was performed as previously described [54]. Viral titers were evaluated by a gradient dilution.

1-day-old female chicks were randomly divided into two groups (Adv-ZFP36L2-AS and Adv-NC, or Lv-shZFP36L2-AS and Lv-shNC; *n* = 30), respectively. Chicks received two intramuscular doses of adenovirus (10^8^ titers)/lentivirus (10^6^ titers) in two different sites of the gastrocnemius. Thirteen days after the initial injection, chick gastrocnemius samples were collected from the above two groups.

### Mitochondrial DNA (mtDNA) content and fatty acid oxidation (FAO) rate assay

Total DNA was extracted using the Tissue DNA Kit (D3396, Omega, GA, USA) according to the manufacturer’s instructions. The amount of mitochondrial DNA was determined by quantification of cytochrome c oxidase subunit II (*COX2*). The nuclear-encoded *β-globin* gene was used as internal controls. Primers used in this study can be found in the Supplementary Table 4.

The mitochondria of myoblast and gastrocnemius was isolated using the Cell/Tissue Mitochondria Isolation Kit (C3601/C3606, Beyotime, China). After measuring the mitochondrial protein concentration, freshly isolated mitochondria were subjected to FAO rate assay with the Colorimetric Fatty Acid Oxidation Rate Assay Kit (HL50679, Haling, Shanghai, China), according to the manufacturer’s protocol.

### Central carbon metabolic profiling

*ZFP36L2-AS* knockdown gastrocnemius samples (*n* = 6) were used for metabolites extraction, and then performed on HPIC-MS/MS analysis. The high-performance ion exchange liquid chromatography (HPIC) separation was carried out using an Thermo Scientific Dionex ICS-6000 HPIC System (Thermo Fisher Scientific, IL, USA). An AB SCIEX 6500 QTRAP + triple quadrupole mass spectrometer (AB Sciex, USA), equipped with an electrospray ionization (ESI) interface, was applied for assay development.

Metabolic hierarchical clustering analysis (HCA) was performed using Cluster3.0 software as previously described [24].

### Hematoxylin and eosin (H&E) staining and immunohistochemistry (IHC)

H&E staining was performed using the Hematoxylin and Eosin Staining Kit (Beyotime, Shanghai, China) following the manufacturer’s protocol. Immunohistochemistry was carried out using SP-POD Kit (SP0041, Solarbio, China) with primary antibodies included anti-MYH1(F59, 1:100, DHSB) and anti-MYH7 (S58, 1:300, DHSB).

### Metabolite and enzyme activities assays

Content of adenosine triphosphate (ATP), triglyceride (TG), free fatty acid (FFA), glycogen, and acetyl-CoA as well as enzyme activity of lactic dehydrogenase (LDH), succinate dehydrogenase (SDH), acetyl-CoA carboxylase (ACC) and pyruvate carboxylase (PC) in skeletal muscle were measured using commercially available kits (BC0305, BC0625, BC0595, BC0345, BC0980, BC0685, BC0955, BC0410 and BC0730, Solarbio, China) according to the manufacturer’s instructions.

### RNA pull-down assay

Ribo™ RNAmax-T7 biotin-labeled transcription kit (RiboBio, Guangzhou, China) was used to harvest biotinylated RNAs. RNA pull-down assays were performed with Pierce Magnetic RNA-Protein Pull-Down Kit (Thermo Fisher Scientific, IL, USA), according to the manufacturer’s instructions. The eluted products were identified by mass spectrometry with a Q Exactive mass spectrometer (Thermo fisher) or western blot. Differentially expressed genes (DEGs) were subjected to enrichment analysis of Gene Ontology (GO) functions and Kyoto Encyclopedia of Genes and Genomes (KEGG) pathways.

### RNA immunoprecipitation (RIP) assay

RIP assays were performed using the Magna RIP™ RNA-Binding Protein Immunoprecipitation Kit (Millipore, CA, USA) following the manufacturer’s protocol. The antibodies used for RIP assays were anti-ACACA (PA5-17564, 1:50, Thermo Fisher Scientific) and anti-PC (GTX132002, 1:100, GeneTex).

### Statistical analysis

In this study, all experiments were repeated at least three times, and results were represented as mean ± SEM. Where applicable, the statistical significance of the data was tested using independent sample *t*-test or ANOVA followed by Dunnett’s test. The types of tests and the *P* values, when applicable, are indicated in the figure legends.

## Supplementary information

aj-checklist

Collated Supplementary Information

Supplementary Information

Supplementary Table 2

Supplementary Table 3

Supplementary Figure 1

Supplementary Figure 2

Supplementary Figure 3

Supplementary Figure 4

Supplementary Figure 5

Supplementary Figure 6

Supplementary Figure 7

Supplementary Figure 8

Supplementary Figure 9

Supplementary Figure 10

Supplementary Figure 11

Supplementary Figure 12

Supplementary Figure 13

Original Data of western blots

## Data Availability

All data generated or analysed during this study are included in this published article (and its supplementary information files). Additional data related to this paper may be available from the corresponding author on reasonable request.
