# Supplementary material for: Long noncoding RNA ZFP36L2-AS functions as a metabolic modulator to regulate muscle development
Source: Cell Death Dis. 2022 Apr 21;13(4):389. doi: 10.1038/s41419-022-04772-2 (PMC9023450; doi:10.1038/s41419-022-04772-2)
Supplement: Supplementary file 2 — Collated Supplementary Information [file 41419_2022_4772_MOESM2_ESM.pdf]

Supplementary Table 1. The full-length sequence of lncRNA *ZFP36L2-AS*. Coordinates are listed according to GRCg6a reference, Annotation Release 104 (chromosome 3: 25089365 to 25092829).

ACAGGGTGCAAAAGGACGGGCCTGCGAGTGCGGGGTGGCTGGGCCCGCCGTCCT
ACATGCAGCCCTGGCTTTAGGGGAGCGGTCACCAGGCCATCCCGGCGGCCTCCCC
TACACGGCAGGCGGAATGTAGGGCAGCAGCTCGGGGTGCAGCCTGTACCAGGCC
CTGCGCCATCACACTGAGCCAGGGCCGCCAGGAGGCTGCAGGGAGGGCATGGAG
CTGGTAGATGTCACTGTCCCACATATACCGTGTGCAGCAGCACGGGAGGTCTTGAT
CTGCTTCAGAGAGAATAAGGGAGTGTCGGGGAAGAGGTGGGCTAAAGCAAGAAG
TGCACCTTGGGAAAGAAAAAAGCCCTCAACTCAACGAGGTGTGCACTCCCAGCT
AACGCAGTTATAGCTCAACAAGTTGTTTCCAAATCCCAAAAGCGAGTCAGGACAT
TGGCCTGGGCGAGCTGCAACCACAGCAGGGCCCCTCCTGCGGGCACCGGGGGGC
CCTCAGAGCAGCTCCTCCAGAACTGCTAAGGAAGCAATCCCCAGCAAATCCCATC
AGGCTAACCGGTAAAAAAGGGCCAATCCCAAGCAAAACCGATCGGACTGACCCA
ATGAAAAAGGCTGGCTGCTGTGGCAGGGCTGTGTTGTCATACATTGCTTACGTTTG
TTTTAATTTTAAACCCAGAGTCAGCCCAAGGTTTGTTACACAAACATATGGCACCC
GTATTACATTCTGCCTCGTATTTATAGAAAGTACCGCGGCAGTCCAGACAGCGCAG
AGATGCCATAACGTTAACAACATTTATTATTAGTATGACAAAAGAACAAAAATAAG
CCTGTAGAAACCACGACTTAAAGTTTAAGGGATTGATTGGAATAAGGAACCGAAC
TGTGAAGCTATTAGTGAAGAAAATCCCTCCAGCATTAACACACGCAATCCCCAGG
CCACCGAGCGTTTTGTTATATAAACACGCCCCCAGAAAGGCAATTTCTTTAAAAAC
TCAAGCCCTCGGTGCTGATTAACACATTTAATGCCAGCCCGATTAGCACTGCTGAG
CCCCAAGCCCAGGGAGCGCTGAGGCAGAAGTCCTCATCCCTCTCTCAGTCCCGCT
GCTGAGGAGAGCGGATCTGGGACAAAGGGTGAATCTTAACGATGCCTTTTTATTG
CCCAAACTGCCACTTCCTCCTCAGCCCAAACAAGCAGGGAAGGGTACCAGCACC
TCCCCGCCAGCGATAAATGCCATTACTAGATTTTCCCATTCCAGCTGCAAAAGGTG
CACGCAGCAGAGGCCTCATCCTCTACCCTCGGCCTAAAAAGAAAGTGGGAAGAA
AAAAGAAGAGGGAAGCAAGCAGGATAGAGCACCCCAGCAGCACTCGCGGGGCC
GAACCTTCTAGAAGAACATATTAGCTTTGGCGGCTGCCCAGCACTGGCTGCAGGA
GCACGCTGGTTAATCTGTAGCCAGACTGAGGGCGGTGGGGAGCTGGACAGGGCC
ACCCCGCAGGCAGGGAAGGGTGAGGGGTGGGGTGGGACGGGCTTGTTGCTTATG
GGTTTCTTTCTTTTTTTTTTTTTTTTCCAAAAAAATTTATTGGGAGAACTACAAA
ACATTTACAGTACAAAGTTTACAGTCTCACACAATTTGTAGTGAACTGACTCCCGA
AAAATATATTACAACTCAAGTTGACTTATCCTTTAGTTACATTCAAAACATACTTCT

GTTAAAGTAGTCCAAAAAGAGTACATAGTGCTCAATCTGTACCTATGTACAAACAA
AACTAAGCTACCGCTCATTCATCCACCGTCCAGGAAAGTTTTGGAAAACTCCCGA
CTTTCTACGCAAGTAAAAAAAAAATAATAATAATATCACAAGTTTTGGAATTCAATT
AAACAAGATATGCTCGTGTATAAAGTTTCAGATCCAAAGATGGCCGCCATTCTTAC
AAAATAAAGAGTGGTGAAATTTTGCTGTAGTCAAAAACCCATCCCTACATTCAAAT
TATCACCTCAAGCATTAAGAGAAATACTCATTTGGTTGAGAAGTATTTAAGGCAAG
CGTGGACCCTCCAGAAGGCACTGTGAGGCATAATACTGGGCACCCCAATTATTGCT
ACATCCGTTGAGACTACATCACAGTGTGAGTGGTGGAGTTAGGCAACGAGGAACA
CTTTTTGGTTACACTTGGAACGAGGCTGCGTCAGGTTCGCCACTTTGAAGTCTGAT
TGCCAAAAGTCAGTCTCCCGTTGTTGGTTTTCTTCTACTCGGACGGGCTACAACCA
TTTACATTCTACAAAAACCCCATAGAAATTCCTCAAACTACTTCCACAGCATCGAG
ACCGATTTCTGTACAGAAACCATGCAATCTTTCAGATTTACGTAAACAAGGAAAGA
AATTAACGAAATAAATATTACATACAATCTCTTAAATTAAGAATTTTTACTCATTTAC
AATAAAATAACCAAGTGAAGTTACAAAAAGGCATATATTACTGTGAAAAGAACAC
ACTCCACGTTTCGCCGATTAATAATGGCAATCATAATTTAAACATAATAAAAGAATA
TATATCTATTGCTTTTCATCATACCCGATAAATACAGTATGAACAAATTACCAATGCA
TACTTTTCACGAGATAATAAATAAGTTAAATAGTTTCATGTTGAGTTGTGTGCAGTG
ACTCATTCATGCAATCAACTCAAAACAGCTAAAAAAAAAAACAATTAAAAAAAAT
CAGCAGTTATTCTCCAACAATTACAAACTAAACTCGGTCGAGTGCTTCCAAATAAA
GCAACAACGAAAAAAAGAAAAGAGTTGTTCTAAGCCAAACATCCACTAAGTGGT
GGCAATGGAACCAATATTAAACTTTGGAATGAAGGTTTTAGCATTTGTTATAATAAT
AATATATAGATATATAAAAAAAATAGAAGGTTGTTATCAAGGCATATTCTTGGCAGG
ATGTTGGATTTCGCGCTGGAGAAGCTCGCGGTGGGTCCGGCCGCGTTCCCGGATG
GAATGGTGCTGCTGGTGCGGATCAGAACTCGGCTCCGGAGAACGAGAAGGACAA
CCCCGAAACACAAATCCCCCTTCTTCCAAGGTCTCTCTAGAAAAATAGGTTTGTTC
AGTTCACGTCAGTCGCCCTTTCGCAAGACGCTTTTCGCTTTGTTCAGCTCTCGTGT
GTACATGGATAGGTTACGCGTATAAAAAAAGACACGACAGGAGACGGACAGAGA
GGGGGGCCGGCTCGCTCCCCGCGCCCCACCGCCCGCGGCCCCCTCCTTCCATCCT
TCCATCCTTCAGTCGTCGGAGATGGAGAGGCGGCTGAAGATGGGCAGGCGGCGG
CCCGAGTCCAGGCCGGGCGACTCGGAGCCGCTGAGCGAGCCGGAGCTGAGCGAG
CCGCTCAGGTAGCTCTCGCGGTCCGAGAGCGAGTCCGGCGGGCTGGGCGGCGCG
TCGAACACGGGCGACTCGGAAAGGCGGCGCAGG

Supplementary Table 2. Comparative metabolome analysis of control group versus lncRNA *ZFP36L2-AS* knockdown gastrocnemius.

Separate Excel file.

Supplementary Table 3. lncRNA *ZFP36L2-AS* specific binding proteins identified by RNA pull-down coupled to mass spectrometry.

Separate Excel file.

Supplementary Table 4. Information of Primers.

**Primer name**	**Primer sequences (5' to 3')**	**Usage**
qPCR-ZFP36L2-AS	F: ACTGTGAGGCATAATACTGGG<br>R: AAAGTGGCGAACCTGACG	qPCR
qPCR-ACACA	F: CCCTGTTTCTGTCTGCTC<br>R: TCAGGACCTTCTCAATAACTC	qPCR
qPCR-PC	F: TCCTGCAAGGCTACATCGG<br>R: CACCTCAAACTCCTCAGCGATA	qPCR
qPCR-CCNB2	F: CAGTAAAGGCTACGAAAG<br>R: ACATCCATAGGGACAGG	qPCR
qPCR-CCND1	F: CAGAAGTGCGAAGAGGAAGT<br>R: CTGATGGAGTTGTCGGTGTA	qPCR
qPCR-CDKN1A	F: CCCGTAGACCACGAGCAGAT<br>R: CGTCTCGGTCTCGAAGTTGA	qPCR
qPCR-CDKN1B	F: TCGCTGTGCTGGGCTGAA<br>R: CAAGGACGAAAGGATGTGGG	qPCR
qPCR-MYOD	F: GCTACTACACGGAATCACCAAAT<br>R: CTGGGCTCCACTGTCACTCA	qPCR
qPCR-MYOG	F: CGGAGGCTGAAGAAGGTGAA<br>R: CGGTCCTCTGCCTGGTCAT	qPCR
qPCR-MyHC	F: CTCCTCACGCTTTGGTAA<br>R: TGATAGTCGTATGGGTTGGT	qPCR
qPCR-COX2	F: GTAGATGCCCAAGAAGTT<br>R: GTTTGATTTAGTCGTCCAG	qPCR
qPCR-β-globin	F: CAGCCAGGTGGAGGATTT<br>R: GAATAGGAGGACCCTCTGTTAG	qPCR
qPCR-CPT1	F: GCTTATTGTAGTTGTGGGTG<br>R: AAAGTTTGCCGTGTTCAG	qPCR
qPCR-FASN	F: CGCAGGCATAGCAGGAAA<br>R: CCAAAGAAGGAGGCATCAA	qPCR
qPCR-MACT	F: GTGCTGGGCTACGACCTGCT	qPCR

	R: CAGTGCCTACGGGCTTCCAG	
qPCR-OXSM	F: ACATAACAGCACCTAATCC<br>R: ATGGGACGTAATTGAGAT	qPCR
qPCR-HK1	F: CTGGATCTCGGTGGTTCTTAC<br>R: TTGTCGGCACGGGAAAGA	qPCR
qPCR-GPI	F: ATTCACTTTGGGAGCAATC<br>R: ACTCCAACTCTGGCTCAAT	qPCR
qPCR-PGAM1	F: GCGAGGCTCAGGTGAAGAT<br>R: GTCCTCCGTCAGGTCAGC	qPCR
qPCR-PGK1	F: CCCTGGATAAGGTGGATG<br>R: TTGTCAGGCATGGGAACT	qPCR
qPCR-PYGL	F: ACATTTGCCTACACGAACC<br>R: TGCCTCCCTCCTCTATCA	qPCR
qPCR-SOX6	F: TCAGGTTCAGGGTCACATGCC<br>R: TTGCTGGAGCTGTAAAGGGC	qPCR
qPCR-TNNC1	F: GTTGAGCAGTTGACAGAAGA<br>R: GAACCATCATAACAAGGAAC	qPCR
qPCR-TNNC2	F: GAGCAGCAAAGATGGCGTCA<br>R: ATCACCGTGCCCAACTCCTT	qPCR
qPCR-TNNI1	F: GAGGAGTGGGAGCAGGAGAT<br>R: TTCGTCCACAATCTCAACCT	qPCR
qPCR-TNNT1	F: GAGCCGCACGGAGAAGGAGC<br>R: CCCGAAGTGGGGCATGTTGG	qPCR
qPCR-TNNT3	F: AGAGGGAAGAAGCAAACAGC<br>R: GTCCCACAGTTCCTTAGCCT	qPCR
qPCR-ATROGIN1	F: TCAACGGGTCGGCAAGTCT<br>R: TCCCTCCCATCGCTCAGTC	qPCR
qPCR-MURF1	F: GGACGAGCGGATCAACAT<br>R: GGGAGATGATGGTCTGGATG	qPCR
qPCR-LC3B	F: GAGCAAAGAGTTGAAGATG<br>R: GTCCTAGACGGAAGATTG	qPCR
qPCR-SQSTM1	F: AGCGACGAGGAGCTGGATC<br>R: CCTTGTGGATGCCTTTACCC	qPCR
qPCR-ULK1	F: TCGTTGCCTTGTATGACTT<br>R: TTTATGCGAATGTTGTTGG	qPCR
qPCR-β-actin	F: GATATTGCTGCGCTCGTTG<br>R: TTCAGGGTCAGGATACCTCTTT	qPCR
5’ RACE-ZFP36L2-AS	Outer: TGGTTGCAGCTCGCCCAGGCCAAT<br>Inner: GCCAATGTCCTGACTCGCTTTTG	RACE
3’ RACE- ZFP36L2-AS	Outer: CACCGCCCGCGGCCCCCTCCTTC<br>Inner: TTCAGTCGTCGGAGATGGAGAGG	RACE

ZFP36L2-AS-ORF1-3xFLAG	F: **AAGCTT**ATGGAGCTGGTAGATGTCACT<br>R: **CTCGAG**TAACTGCGTTAGCTGGGAG	Vector construction
ZFP36L2-AS-ORF2-3xFLAG	F: **AAGCTT**ATGAAAAAGGCTGGCTG<br>R: **CTCGAG**ATAATAAATGTTGTTAACG	Vector construction
ZFP36L2-AS-ORF3-3xFLAG	F: **AAGCTT**ATGCCTTTTTATTGCCCA<br>R: **CTCGAG**TCGCTGGCGGGGAGGTG	Vector construction
ZFP36L2-AS-ORF4-3xFLAG	F: **AAGCTT**ATGCCATTACTAGATTTTCC<br>R: **CTCGAG**ATATGTTCTTCTAGAAGGTTCG	Vector construction
ZFP36L2-AS-ORF5-3xFLAG	F: **AAGCTT**ATGGGTTTCTTTCTTTTT<br>R: **CTCGAG**CTTTAACAGAAGTATGTTTTG	Vector construction
ZFP36L2-AS-ORF6-3xFLAG	F: **AAGCTT**ATGAACAAATTACCAATGC<br>R: **CTCGAG**CTGCACACAACTCAACATG	Vector construction
ZFP36L2-AS-ORF7-3xFLAG	F: **AAGCTT**ATGGAACCAATATTAAACT<br>R: **CTCGAG**TTTTTCTAGAGAGACCTTG	Vector construction
ZFP36L2-AS-ORF8-3xFLAG	F: **AAGCTT**ATGAAGGTTTTAGCATTTG<br>R: **CTCGAG**TTTTTCTAGAGAGACCTTGG	Vector construction
ZFP36L2-AS-ORF9-3xFLAG	F: **AAGCTT**ATGTTGGATTTCGCGCT<br>R: **CTCGAG**TACGCGTAACCTATCCATGTA	Vector construction
ZFP36L2-AS-ORF10-3xFLAG	F: **AAGCTT**ATGGAATGGTGCTGCTGG<br>R: **CTCGAG**TTTTTCTAGAGAGACCTTGGAAG	Vector construction
ZFP36L2-AS-ORF11-3xFLAG	F: **AAGCTT**ATGGTGCTGCTGGTGCG<br>R: **CTCGAG**TACGCGTAACCTATCCATGTAC	Vector construction
ZFP36L2-AS-ORF12-3xFLAG	F: **AAGCTT**ATGGAGAGGCGGCTGAAGAT<br>R: **CTCGAG**CCTGAGCGGCTCGCTCA	Vector construction
ZFP36L2-AS-ORF13-3xFLAG	F: **AAGCTT**ATGGGCAGGCGGCGGC<br>R: **CTCGAG**CCTGAGCGGCTCGCTCAGCT	Vector construction
pcDNA3.1- ZFP36L2-AS	F: **AAGCTT**ACAGGGTGCAAAAGGACGGGC<br>R: **GCTAGC**ATGTCGCAGCTGTGTGTCCCC	Vector construction
pcDNA3.1-PC	F: **GCTAGC**ATGAACTACATAAACTCAAAAT<br>R: **AAGCTT**TTACTCGATCTCGGCGATGAGG	Vector construction
pDC316-mCMV-ZsGreen-ZFP36L2-AS	F: **GCTAGC**ACAGGGTGCAAAAGGACGGGCCTGCGAG<br>R: **AAGCTT**CCTGCGCCGCCTTTCCGAGTCGCCCGTGT	Vector construction
pLVX-shRNA2-Puro-ZFP36L2-AS	F: **GATCC**CCCGATAAATACAGTATGA**TTCAAGAGA**TCATACTGTATTTATCGGGTTTTT**G**<br>R: **AATTC**AAAAAACCCGATAAATACAGTATGA**TCTCTTGAA**TCATACTGTATTTATCGGG**G**	Vector construction

Sequences in bold represent the enzyme cutting sites.

Supplementary Table 5. Oligonucleotide sequences in this study.

Fragment name	Sequences (5' to 3')
si-ZFP36L2-AS	CCCGATAAATACAGTATGA
ASO-ZFP36L2-AS	AGTCAAAAACCCATCCCTAC
si-ACACA	ACGTATCACCAGTGAGAAT
si-PC	TCGAATTCCTGCAAGGCTA

Supplementary Figure 1. Characterization of the lncRNA *ZFP26L2-AS*. (**A**) Conservative analysis of *ZFP26L2-AS* performed by using the NCBI's BLAST. A total of eighteen species, including *Anas platyrhynchos*, *Anser cygnoides*, *Apteryx mantelli mantelli*, *Aquila chrysaetos*, *Bos taurus*, *Coturnix japonica*, *Gallus gallus*, *Geospiza fortis*, *Homo sapiens*, *Meleagris gallopavo*, *Melopsittacus undulatus*, *Mus musculus*, *Numida meleagris*, *Ovis aries*, *Pan troglodytes*, *Rattus norvegicus*, *Sus scrofa* and *Zebra finch* were used for Nucleotide BLAST. Top 5 most conservative results were listed above. (**B**) Relative *ZFP26L2-AS* expression in muscle-resident cells.

Supplementary Figure 2. Overexpression of *ZFP26L2-AS* inhibits myoblast proliferation but promotes myogenic differentiation. (**A-K**) Relative *ZFP26L2-AS* expression (**A**), EdU proliferation assays (**B**), proliferation rate of myoblasts (**C**), CCK-8 assays (**D**), cell cycle analysis (**E**), relative mRNA levels of several cell cycle genes (**F**), MyHC immunostaining (**G**), myotube area (**H**), myoblast fusion index (**I**) and relative mRNA (**J**) and protein (**K**) expression levels of myoblast differentiation marker genes with *ZFP26L2-AS* overexpression *in vitro*. In panel (**K**), the numbers shown below the bands were folds of band intensities relative to control. Band intensities were quantified by ImageJ and normalized to β-Tubulin. Data are expressed as a fold-change relative to the control. Results are presented as mean ± SEM. In panels (**A**, **C-F**, and **H-J**), statistical significance of differences between means was assessed using independent sample *t*-test.

Supplementary Figure 3. Overexpression of *ZFP26L2-AS* represses cellular respiration and fatty acid oxidation in skeletal muscle. (**A-E**) OCR (**A**), basal respiration, maximal respiration and ATP production (**B**), ECAR (**C**), glycolysis and glycolytic capacity (**D**), and relative cellular ATP content (**E**) with *ZFP26L2-AS* overexpression in CPMs. (**F-K**) Relative *ZFP26L2-AS* expression (**F**), relative mtDNA content (**G**), relative fatty acid β-oxidation rate (**H**), relative mRNA (**I**) and protein (**J**) expression levels of fatty acid oxidation or synthesis related-genes, and relative FFA and TG content (**K**) in gastrocnemius with *ZFP26L2-AS* overexpression *in vivo*. In panel (**J**), the numbers shown below the bands were folds of band intensities relative to control. Band intensities were quantified by ImageJ and normalized to β-Tubulin. Data are expressed as a fold-change relative to the control. Results are shown as mean ± SEM. In panels (**B**, **D-I** and **K**), statistical significance of differences between means was assessed using independent sample *t*-test.

Supplementary Figure 4. *ZFP36L2-AS* does not regulate cellular ATP content in satellite cells. (**A** and **B**) Relative cellular ATP content with *ZFP26L2-AS* interference (**A**) and overexpression (**B**) in satellite cells. Results are shown as mean ± SEM. In all panels, statistical significance of differences between means was assessed using independent sample *t*-test.

Supplementary Figure 5. Overexpression of *ZFP36L2-AS* activates fast-twitch muscle phenotype and induces muscle atrophy. (**A-L**) Relative glycogen content (**A**), relative mRNA expression levels of glycogenolytic and glycolytic genes (**B**), relative enzymes activity of LDH and SDH (**C**), immunohistochemistry analysis of MYH1/MYH7 (**D**), MYH1/MYH7 protein content (**E**), relative mRNA expression levels of several fast-/slow-twitch myofiber genes (**F**), relative gastrocnemius muscle weight (**G**), H&E staining (**H**) and frequency distribution of fiber CSA (**I**), relative mRNA expression of the atrophy and autophagy-related genes (**J**), and the protein expression levels of mTOR signaling in gastrocnemius with *ZFP36L2-AS* overexpression *in vivo*. In panel (**J**), the numbers shown below the bands were folds of band intensities relative to control. Band intensities were quantified by ImageJ and normalized to β-Tubulin. Data are expressed as a fold-change relative to the control. Results are shown as mean ± SEM. In panels (**A-C**, **E-G**, and **J**), statistical significance of differences between means was assessed using independent sample *t*-test.

Supplementary Figure 6. GO functions and KEGG pathways analysis of lncRNA *ZFP36L2-AS* specific binding proteins identified by RNA pull-down coupled to mass spectrometry. (**A**) GO functions analysis of lncRNA *ZFP36L2-AS* specific binding proteins identified by RNA pull-down coupled to mass spectrometry. (**B**) KEGG pathways analysis of lncRNA *ZFP36L2-AS* specific binding proteins identified by RNA pull-down coupled to mass spectrometry.

Supplementary Figure 7. lncRNA *ZFP36L2-AS* 816-1785 region is necessary for *ZFP36L2-AS* to function. (**A-K**) Relative *ZFP26L2-AS* 816-1785 region expression (**A**), relative mRNA levels of several cell cycle genes (**B**), relative mRNA expression levels of myoblast differentiation marker genes (**C**), relative mRNA expression levels of fatty acid oxidation or synthesis related-genes (**D**), relative mRNA expression levels of several fast-/slow-twitch myofiber genes (**E**), relative mRNA expression of the atrophy and autophagy-related genes (**F**), the protein expression levels of ACACA and phosphorylated ACACA (**G**), relative ACACA activity (**H**), the protein expression level of PC (**I**), relative PC activity (**J**), and relative acetyl-CoA content (**K**) with *ZFP26L2-AS* 816-1785 region overexpression *in vitro*. In panels (**G** and

I), the numbers shown below the bands were folds of band intensities relative to control. Band intensities were quantified by ImageJ and normalized to β-Tubulin. Data are expressed as a fold-change relative to the control. Results are presented as mean ± SEM. In panels (**A**-**F**, **H**, **J** and **K**), statistical significance of differences between means was assessed using independent sample *t*-test.

Supplementary Figure 8. The mRNA level of *ACACA* and *PC* didn't change with *ZFP36L2-AS* overexpression and knockdown both *in vitro* and *in vivo*. (**A** and **B**) The mRNA level of *ACACA* with *ZFP36L2-AS* overexpression and knockdown *in vitro* (**A**) and *in vivo* (**B**). (**C** and **D**) The mRNA level of *PC* with *ZFP36L2-AS* overexpression and knockdown *in vitro* (**C**) and *in vivo* (**D**). Results are shown as mean ± SEM. In all panels, statistical significance of differences between means was assessed using independent sample *t*-test.

Supplementary Figure 9. Interference of *ACACA* promotes myoblast proliferation and inhibits myoblast differentiation. (**A**-**L**) Relative mRNA (**A**) and protein (**B**) expression levels of *ACACA*, EdU proliferation assays (**C**), proliferation rate of myoblasts (**D**), CCK-8 assays (**E**), cell cycle analysis (**F**), relative mRNA levels of several cell cycle genes (**G**), MyHC immunostaining (**H**), myotube area (**I**), myoblast fusion index (**J**) and relative mRNA (**K**) and protein (**L**) expression levels of myoblast differentiation marker genes with *ACACA* interference *in vitro*. In panels (**B** and **L**), the numbers shown below the bands were folds of band intensities relative to control. Band intensities were quantified by ImageJ and normalized to β-Tubulin. Data are expressed as a fold-change relative to the control. Results are presented as mean ± SEM. In panels (**A**, **D**-**G**, and **I**-**K**), statistical significance of differences between means was assessed using independent sample *t*-test.

Supplementary Figure 10. The expression and location analysis of *ACACA* and *PC*. (**A** and **D**) Tissue expression profiles of *ACACA* (**A**) and *PC* (**D**). The horizontal axis and vertical axis indicate different tissues and their relative expression values, respectively. (**B** and **E**) Relative *ACACA* (**B**) and *PC* (**E**) expression during CPM proliferation and differentiation. (**C** and **F**) Subcellular location of ACACA and PC protein annotated by UniProt Knowledgebase (https://www.uniprot.org/). In panels (**A**, **B**, **D**, and **E**), results are presented as mean ± SEM.

Supplementary Figure 11. *PC* promotes myoblast proliferation and inhibits myoblast differentiation. (**A**-**X**) Relative mRNA (**A** or **M**) and protein (**B** or **N**) expression levels of *PC*,

EdU proliferation assays (**C** or **O**), proliferation rate of myoblasts (**D** or **P**), CCK-8 assays (**E** or **Q**), cell cycle analysis (**F** or **R**), relative mRNA levels of several cell cycle genes (**G** or **S**), MyHC immunostaining (**H** or **T**), myotube area (**I** or **U**), myoblast fusion index (**J** or **V**) and relative mRNA (**K** or **W**) and protein (**L** or **X**) expression levels of myoblast differentiation marker genes with *PC* overexpression or interference *in vitro*. In panels (**B**, **L**, **N**, and **X**), the numbers shown below the bands were folds of band intensities relative to control. Band intensities were quantified by ImageJ and normalized to β-Tubulin. Data are expressed as a fold-change relative to the control. Results are presented as mean ± SEM. In panels (**A**, **D**-**G**, **I**-**K**, **M**, **P**-**S** and **U**-**W**), statistical significance of differences between means was assessed using independent sample *t*-test.

Supplementary Figure 12. *PC* promotes cellular mitochondrial respiration and decreases glycolytic capacity. (**A**-**H**) OCR (**A** or **E**), basal respiration, maximal respiration and ATP production (**B** or **F**), ECAR (**C** or **G**), and glycolysis and glycolytic capacity (**D** or **H**) of CPMs with *PC* overexpression or interference. Results are shown as mean ± SEM. In all panels, statistical significance of differences between means was assessed using independent sample *t*-test.

Supplementary Figure 13. Model of lncRNA *ZFP36L2-AS* interacts with ACACA and PC to facilitate intramuscular fat deposition, as well as activate fast-twitch muscle phenotype and induce muscle atrophy.

A

Organism	Max Score	Total Score	Query Cover	E value	Percent Identity	Accession
Gallus gallus	6399	6399	100%	0.0	100.00%	NC_006090.5
Coturnix japonica	4835	4835	100%	0.0	91.92%	NC_029518.1
Numida meleagris	4669	4669	100%	0.0	91.23%	NC_034411.1
Meleagris gallopavo	4573	4573	92%	0.0	92.87%	NC_015012.2
Anas platyrhynchos	1122	2951	66%	0.0	95.17%	NC_051774.1

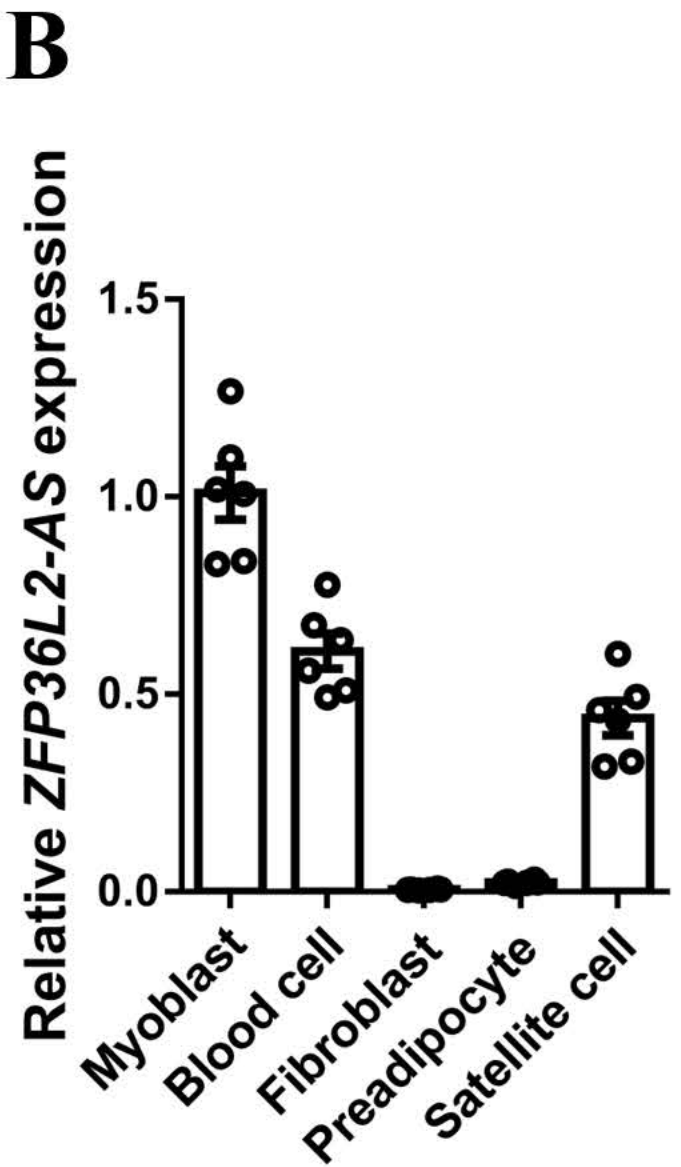

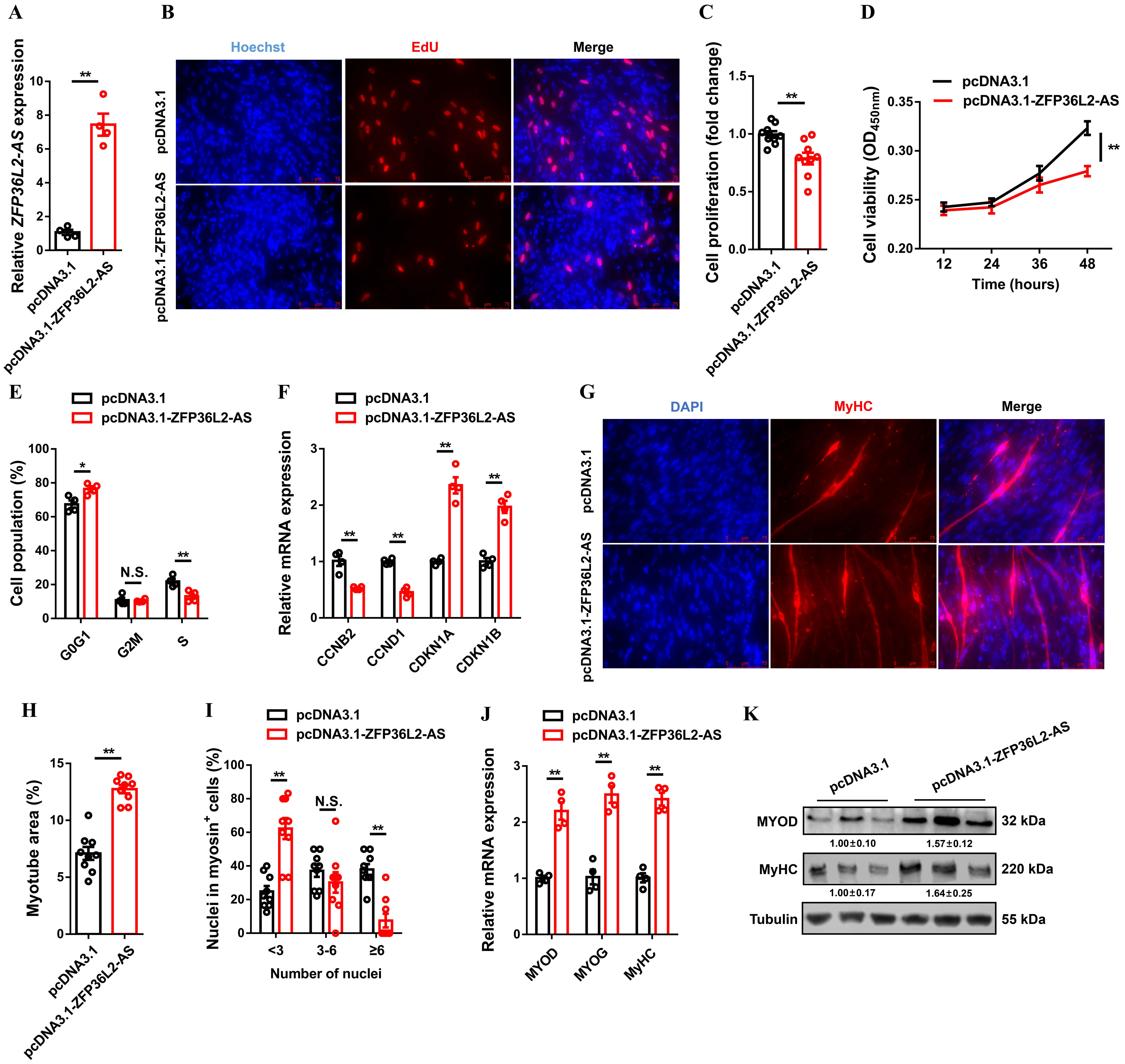

A
Relative ZFP36L2-AS expression
pcDNA3.1
pcDNA3.1-ZFP36L2-AS
B
Hoechst
EdU
Merge
C
Cell proliferation (fold change)
D
Cell viability (OD450nm)
Time (hours)
E
Cell population (%)
G0G1
G2M
S
N.S.
F
Relative mRNA expression
CCNB2
CCND1
CDKN1A
CDKN1B
G
DAPI
MyHC
Merge
H
Myotube area (%)
I
Nuclei in myosin+ cells (%)
Number of nuclei
<3
3-6
≥6
J
MYOD
MYOG
MyHC
K
MYOD
32 kDa
1.00±0.10
1.57±0.12
220 kDa
1.00±0.17
1.64±0.25
Tubulin
55 kDa

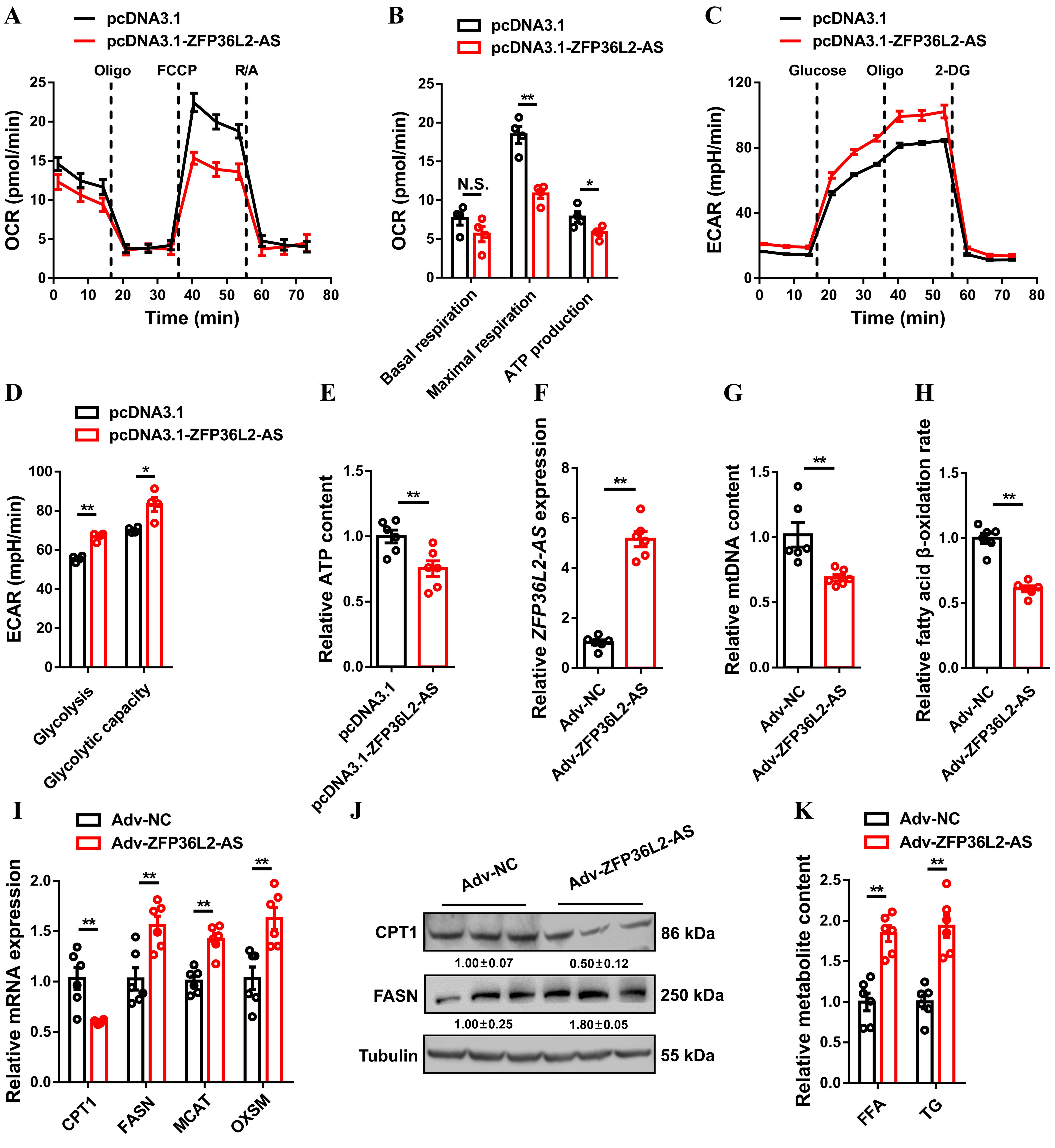

A
pcDNA3.1
pcDNA3.1-ZFP36L2-AS
Oligo
FCCP
R/A
OCR (pmol/min)
Time (min)
B
pcDNA3.1
pcDNA3.1-ZFP36L2-AS
N.S.
**
*
OCR (pmol/min)
Basal respiration
Maximal respiration
ATP production
C
pcDNA3.1
pcDNA3.1-ZFP36L2-AS
Glucose
Oligo
2-DG
ECAR (mpH/min)
Time (min)
D
pcDNA3.1
pcDNA3.1-ZFP36L2-AS
**
*
ECAR (mpH/min)
Glycolysis
Glycolytic capacity
E
**
Relative ATP content
pcDNA3.1
pcDNA3.1-ZFP36L2-AS
F
**
Relative ZFP36L2-AS expression
Adv-NC
Adv-ZFP36L2-AS
G
**
Relative mtDNA content
Adv-NC
Adv-ZFP36L2-AS
H
**
Relative fatty acid β-oxidation rate
Adv-NC
Adv-ZFP36L2-AS
I
Adv-NC
Adv-ZFP36L2-AS
**
Relative mRNA expression
CPT1
FASN
MCAT
OXSM
J
Adv-NC
Adv-ZFP36L2-AS
CPT1
86 kDa
1.00±0.07
0.50±0.12
FASN
250 kDa
1.00±0.25
1.80±0.05
Tubulin
55 kDa
K
Adv-NC
Adv-ZFP36L2-AS
**
Relative metabolite content
FFA
TG

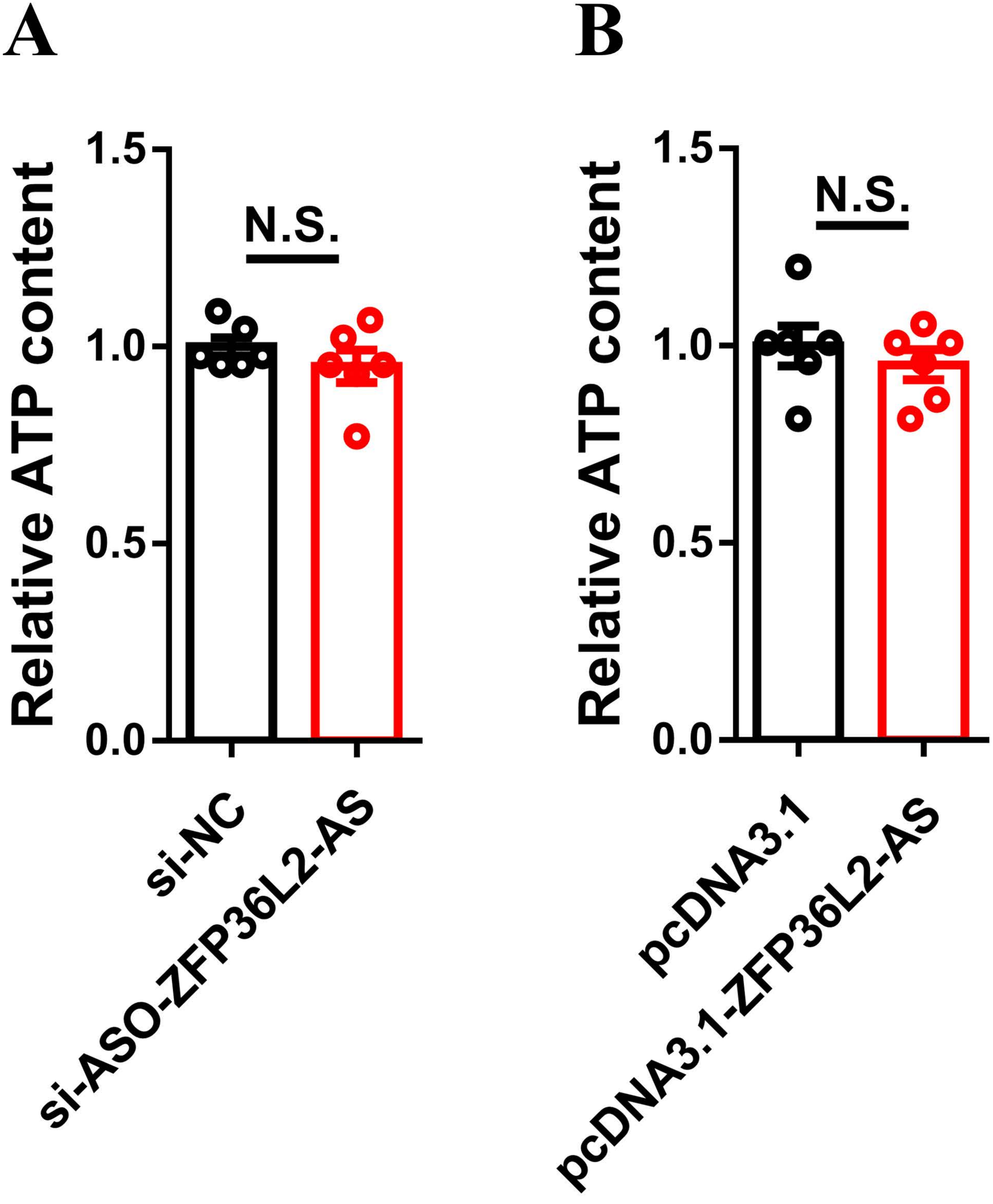
A
B
Relative ATP content
1.5
1.0
0.5
0.0
N.S.
si-NC
si-ASO-ZFP36L2-AS
pcDNA3.1
pcDNA3.1-ZFP36L2-AS

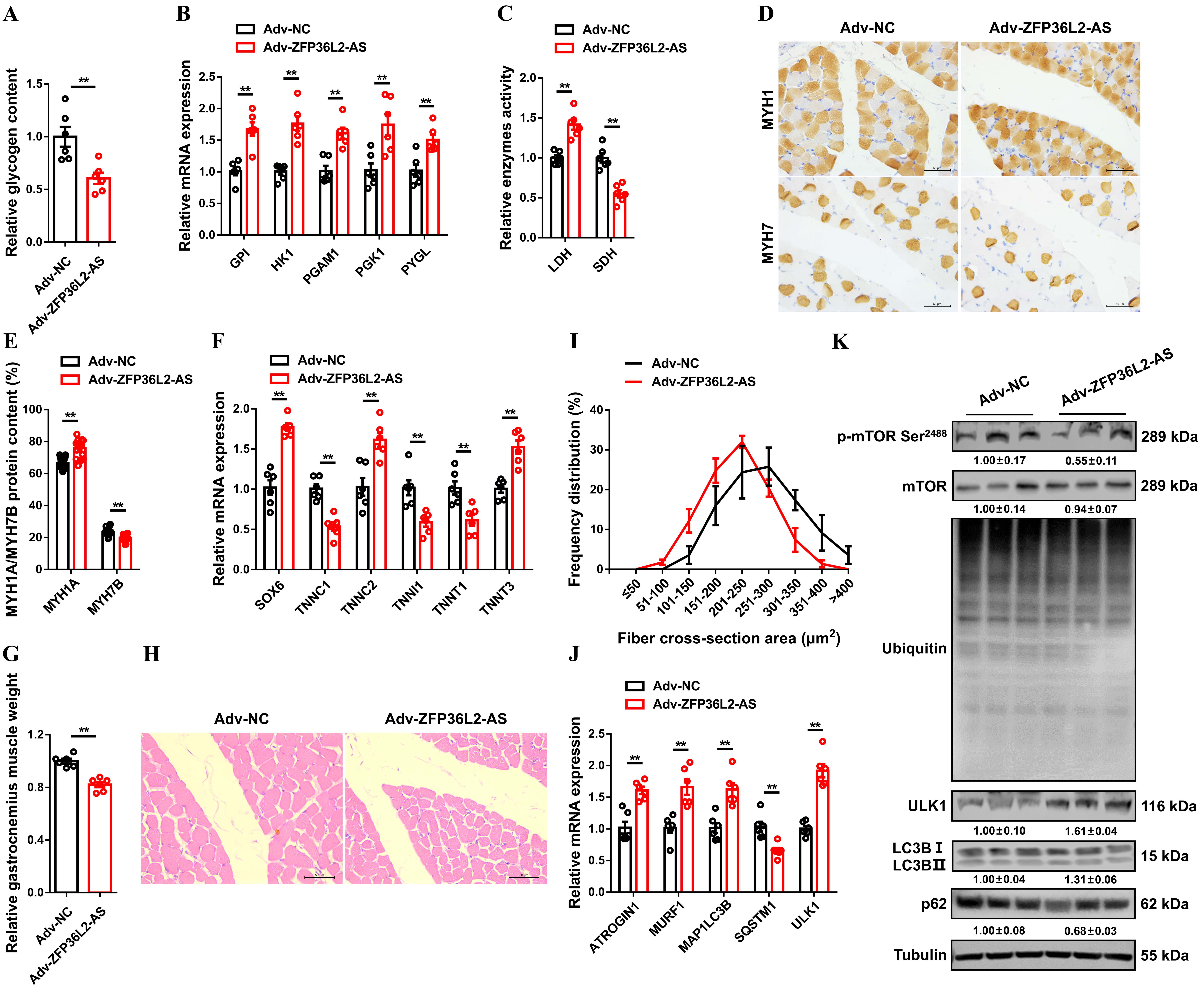

A
Relative glycogen content
Adv-NC
Adv-ZFP36L2-AS
B
Adv-NC
Adv-ZFP36L2-AS
Relative mRNA expression
GPI
HK1
PGAM1
PGK1
PYGL
C
Adv-NC
Adv-ZFP36L2-AS
Relative enzymes activity
LDH
SDH
D
Adv-NC
Adv-ZFP36L2-AS
MYH1
MYH7
E
Adv-NC
Adv-ZFP36L2-AS
MYH1A/MYH7B protein content (%)
MYH1A
MYH7B
F
Adv-NC
Adv-ZFP36L2-AS
Relative mRNA expression
SOX6
TNNC1
TNNC2
TNNI1
TNNT1
TNNT3
I
Adv-NC
Adv-ZFP36L2-AS
Frequency distribution (%)
≤50
51-100
101-150
151-200
201-250
251-300
301-350
351-400
>400
Fiber cross-section area (μm2)
K
Adv-NC
Adv-ZFP36L2-AS
p-mTOR Ser2488
289 kDa
1.00±0.17
0.55±0.11
mTOR
289 kDa
1.00±0.14
0.94±0.07
Ubiquitin
ULK1
116 kDa
1.00±0.10
1.61±0.04
LC3B Ⅰ
LC3B Ⅱ
15 kDa
1.00±0.04
1.31±0.06
p62
62 kDa
1.00±0.08
0.68±0.03
Tubulin
55 kDa
G
Relative gastrocnemius muscle weight
Adv-NC
Adv-ZFP36L2-AS
H
Adv-NC
Adv-ZFP36L2-AS
J
Adv-NC
Adv-ZFP36L2-AS
Relative mRNA expression
ATROGIN1
MURF1
MAP1LC3B
SQSTM1
ULK1

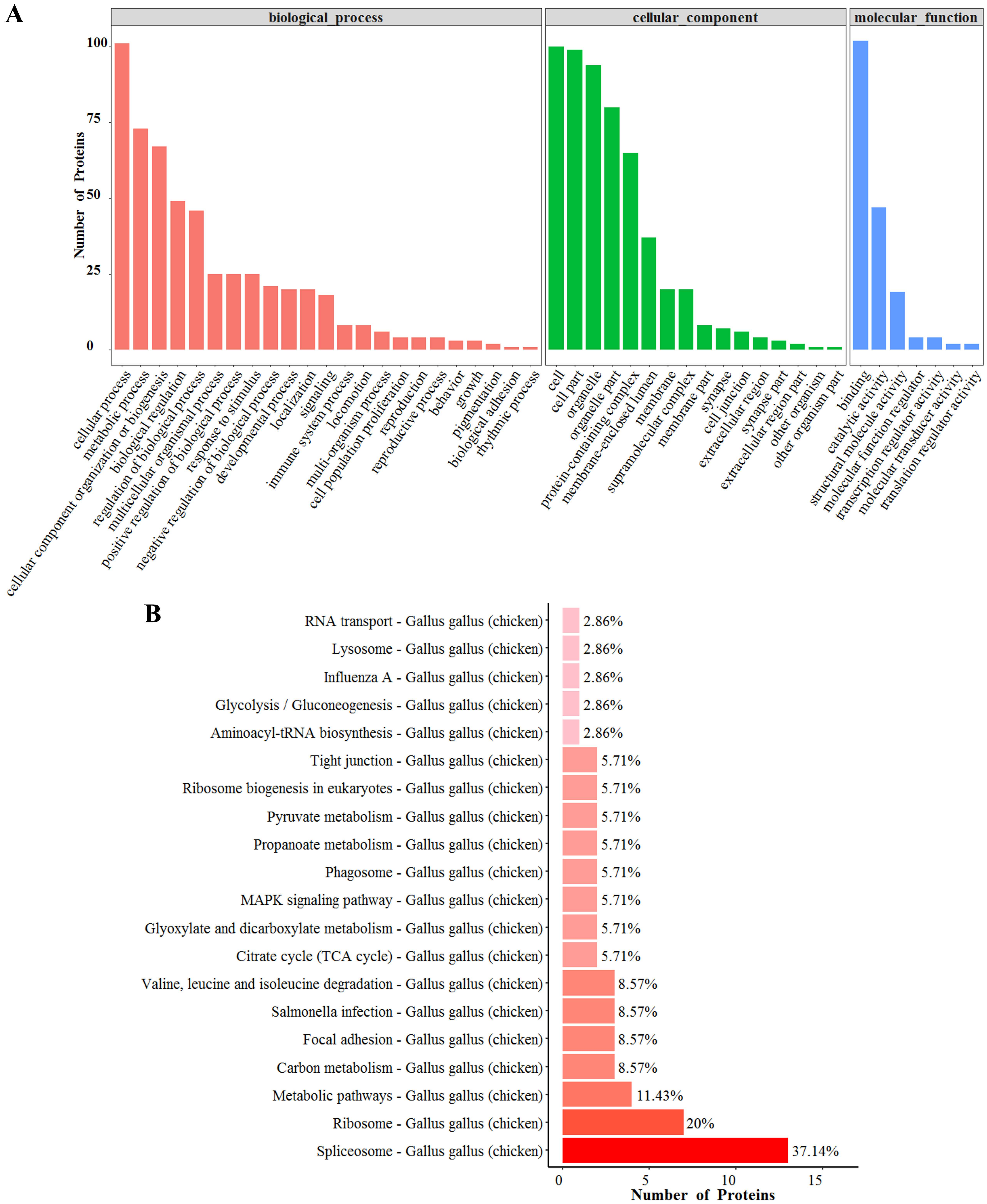

A
biological_process
cellular_component
molecular_function
Number of Proteins
100
75
50
25
0
cellular process
metabolic process
cellular component organization or biogenesis
biological regulation
regulation of biological process
multicellular organismal process
positive regulation of biological process
response to stimulus
negative regulation of biological process
developmental process
localization
signaling
immune system process
locomotion
multi-organism process
cell population proliferation
reproduction
reproductive process
behavior
growth
pigmentation
biological adhesion
rhythmic process
cell
cell part
organelle
organelle part
protein-containing complex
membrane-enclosed lumen
membrane
supramolecular complex
membrane part
synapse
cell junction
extracellular region
synapse part
extracellular region part
other organism
other organism part
binding
catalytic activity
structural molecule activity
molecular function regulator
transcription regulator activity
molecular transducer activity
translation regulator activity
B
RNA transport - Gallus gallus (chicken) 2.86%
Lysosome - Gallus gallus (chicken) 2.86%
Influenza A - Gallus gallus (chicken) 2.86%
Glycolysis / Gluconeogenesis - Gallus gallus (chicken) 2.86%
Aminoacyl-tRNA biosynthesis - Gallus gallus (chicken) 2.86%
Tight junction - Gallus gallus (chicken) 5.71%
Ribosome biogenesis in eukaryotes - Gallus gallus (chicken) 5.71%
Pyruvate metabolism - Gallus gallus (chicken) 5.71%
Propanoate metabolism - Gallus gallus (chicken) 5.71%
Phagosome - Gallus gallus (chicken) 5.71%
MAPK signaling pathway - Gallus gallus (chicken) 5.71%
Glyoxylate and dicarboxylate metabolism - Gallus gallus (chicken) 5.71%
Citrate cycle (TCA cycle) - Gallus gallus (chicken) 5.71%
Valine, leucine and isoleucine degradation - Gallus gallus (chicken) 8.57%
Salmonella infection - Gallus gallus (chicken) 8.57%
Focal adhesion - Gallus gallus (chicken) 8.57%
Carbon metabolism - Gallus gallus (chicken) 8.57%
Metabolic pathways - Gallus gallus (chicken) 11.43%
Ribosome - Gallus gallus (chicken) 20%
Spliceosome - Gallus gallus (chicken) 37.14%
0
5
10
15
Number of Proteins

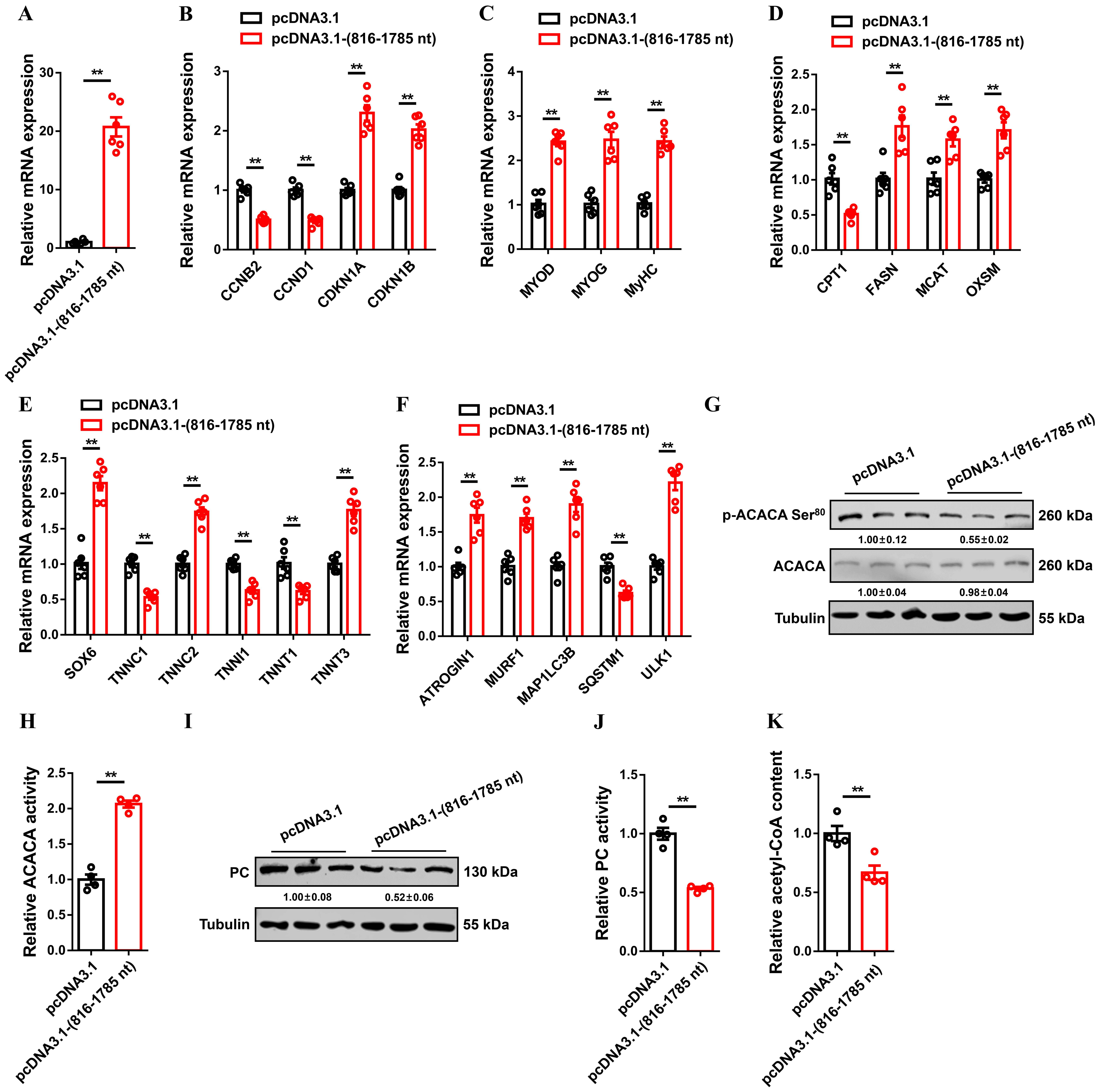
A
Relative mRNA expression
pcDNA3.1
pcDNA3.1-(816-1785 nt)
**
B
pcDNA3.1
pcDNA3.1-(816-1785 nt)
Relative mRNA expression
CCNB2
CCND1
CDKN1A
CDKN1B
C
pcDNA3.1
pcDNA3.1-(816-1785 nt)
Relative mRNA expression
MYOD
MYOG
MyHC
D
pcDNA3.1
pcDNA3.1-(816-1785 nt)
Relative mRNA expression
CPT1
FASN
MCAT
OXSM
E
pcDNA3.1
pcDNA3.1-(816-1785 nt)
Relative mRNA expression
SOX6
TNNC1
TNNC2
TNNI1
TNNT1
TNNT3
F
pcDNA3.1
pcDNA3.1-(816-1785 nt)
Relative mRNA expression
ATROGIN1
MURF1
MAP1LC3B
SQSTM1
ULK1
G
pcDNA3.1
pcDNA3.1-(816-1785 nt)
p-ACACA Ser80
260 kDa
1.00±0.12
0.55±0.02
ACACA
260 kDa
1.00±0.04
0.98±0.04
Tubulin
55 kDa
H
Relative ACACA activity
pcDNA3.1
pcDNA3.1-(816-1785 nt)
I
pcDNA3.1
pcDNA3.1-(816-1785 nt)
PC
130 kDa
1.00±0.08
0.52±0.06
Tubulin
55 kDa
J
Relative PC activity
pcDNA3.1
pcDNA3.1-(816-1785 nt)
K
Relative acetyl-CoA content
pcDNA3.1
pcDNA3.1-(816-1785 nt)

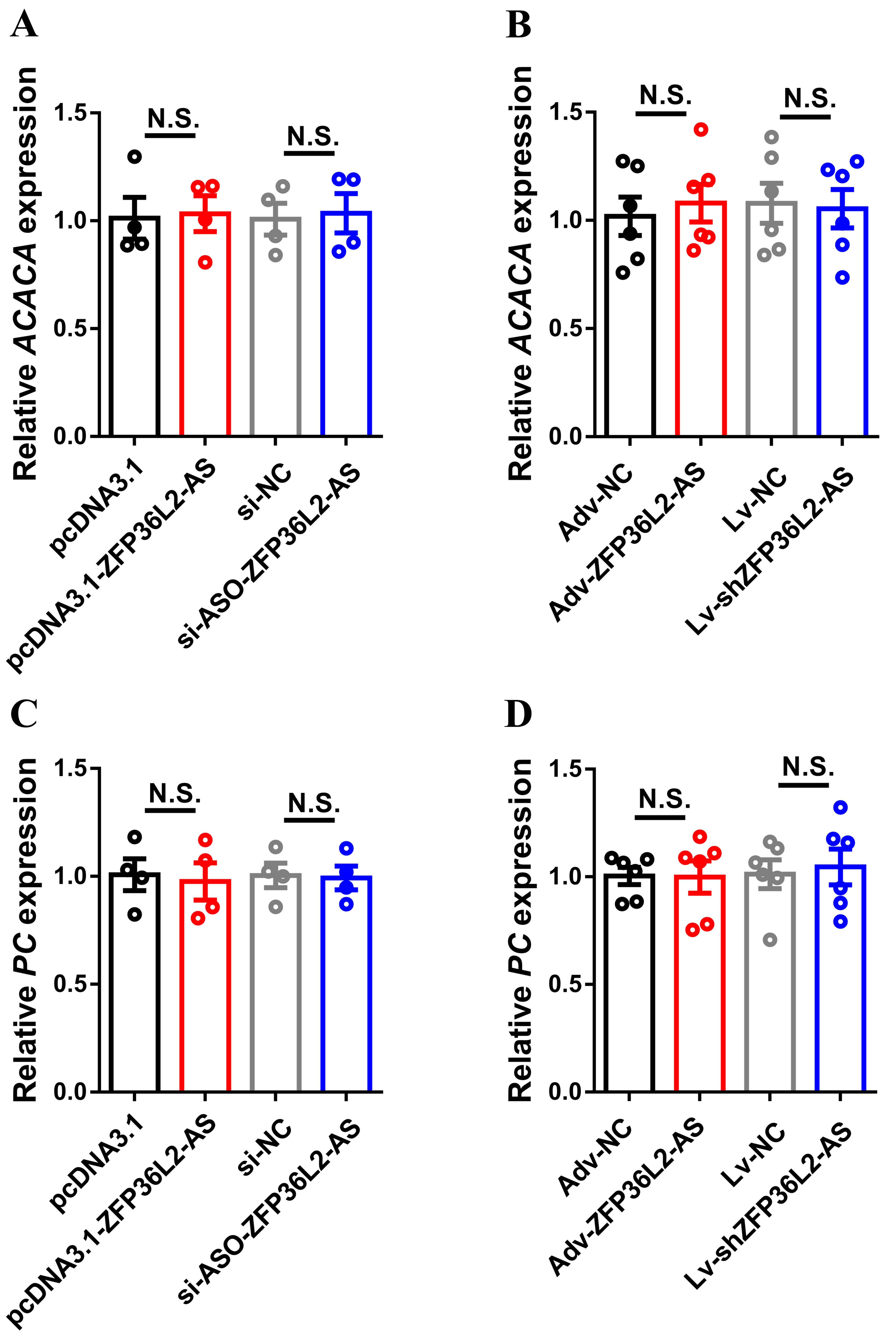
A
Relative ACACA expression
1.5
1.0
0.5
0.0
N.S.
N.S.
pcDNA3.1
pcDNA3.1-ZFP36L2-AS
si-NC
si-ASO-ZFP36L2-AS
B
Relative ACACA expression
1.5
1.0
0.5
0.0
N.S.
N.S.
Adv-NC
Adv-ZFP36L2-AS
Lv-NC
Lv-shZFP36L2-AS
C
Relative PC expression
1.5
1.0
0.5
0.0
N.S.
N.S.
pcDNA3.1
pcDNA3.1-ZFP36L2-AS
si-NC
si-ASO-ZFP36L2-AS
D
Relative PC expression
1.5
1.0
0.5
0.0
N.S.
N.S.
Adv-NC
Adv-ZFP36L2-AS
Lv-NC
Lv-shZFP36L2-AS

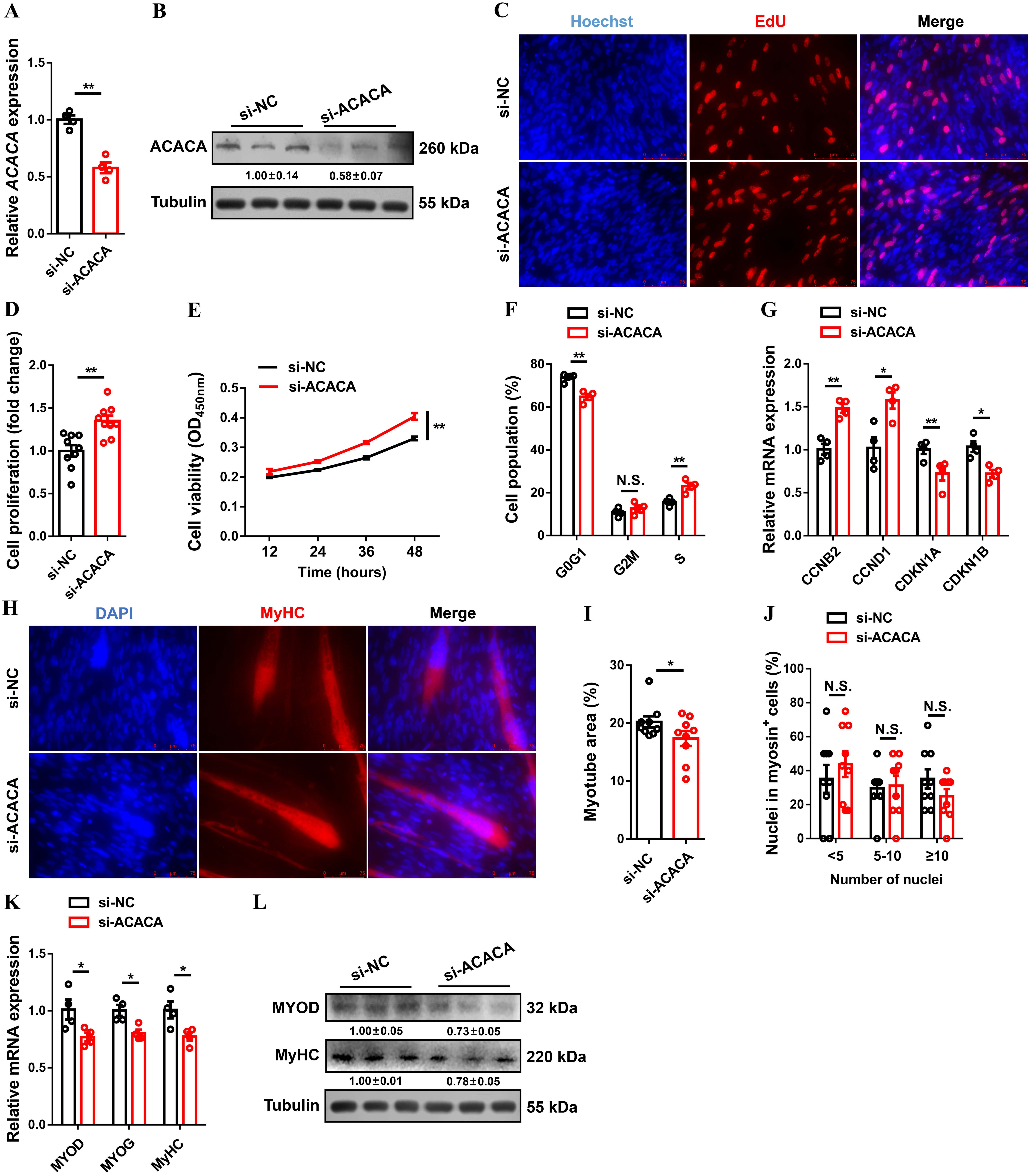

A
Relative ACACA expression
si-NC
si-ACACA
**
B
si-NC
si-ACACA
ACACA
260 kDa
1.00±0.14
0.58±0.07
Tubulin
55 kDa
C
Hoechst
EdU
Merge
si-NC
si-ACACA
D
Cell proliferation (fold change)
E
Cell viability (OD450nm)
Time (hours)
F
Cell population (%)
G0G1
G2M
S
N.S.
G
Relative mRNA expression
CCNB2
CCND1
CDKN1A
CDKN1B
H
DAPI
MyHC
Merge
I
Myotube area (%)
J
Nuclei in myosin+ cells (%)
Number of nuclei
<5
5-10
≥10
K
Relative mRNA expression
MYOD
MYOG
MyHC
L
MYOD
32 kDa
1.00±0.05
0.73±0.05
MyHC
220 kDa
1.00±0.01
0.78±0.05
Tubulin
55 kDa

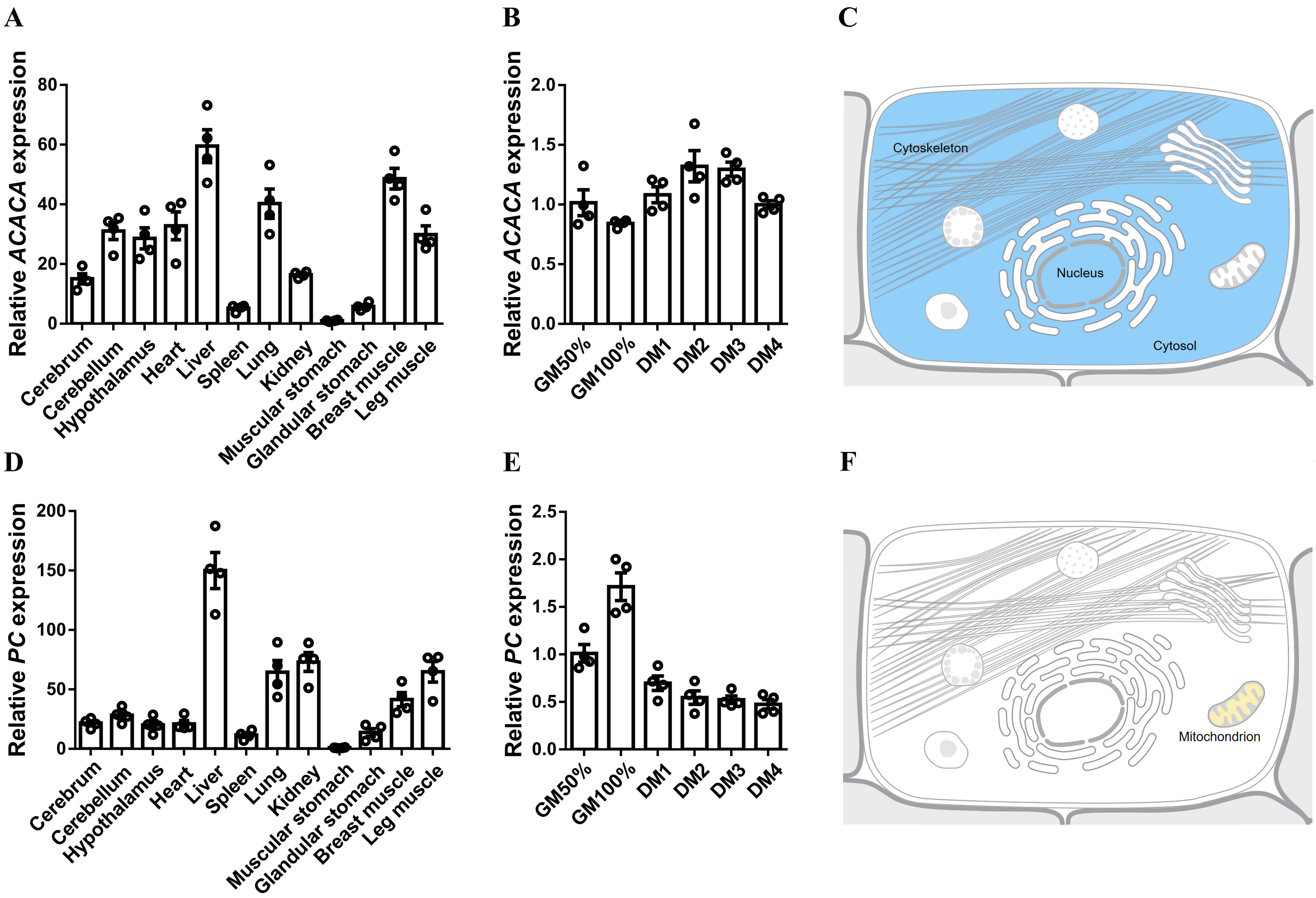

A
Relative ACACA expression
0
20
40
60
80
Cerebrum
Cerebellum
Hypothalamus
Heart
Liver
Spleen
Lung
Kidney
Muscular stomach
Glandular stomach
Breast muscle
Leg muscle
B
Relative ACACA expression
0.0
0.5
1.0
1.5
2.0
GM50%
GM100%
DM1
DM2
DM3
DM4
C
Cytoskeleton
Nucleus
Cytosol
D
Relative PC expression
0
50
100
150
200
Cerebrum
Cerebellum
Hypothalamus
Heart
Liver
Spleen
Lung
Kidney
Muscular stomach
Glandular stomach
Breast muscle
Leg muscle
E
Relative PC expression
0.0
0.5
1.0
1.5
2.0
2.5
GM50%
GM100%
DM1
DM2
DM3
DM4
F
Mitochondrion

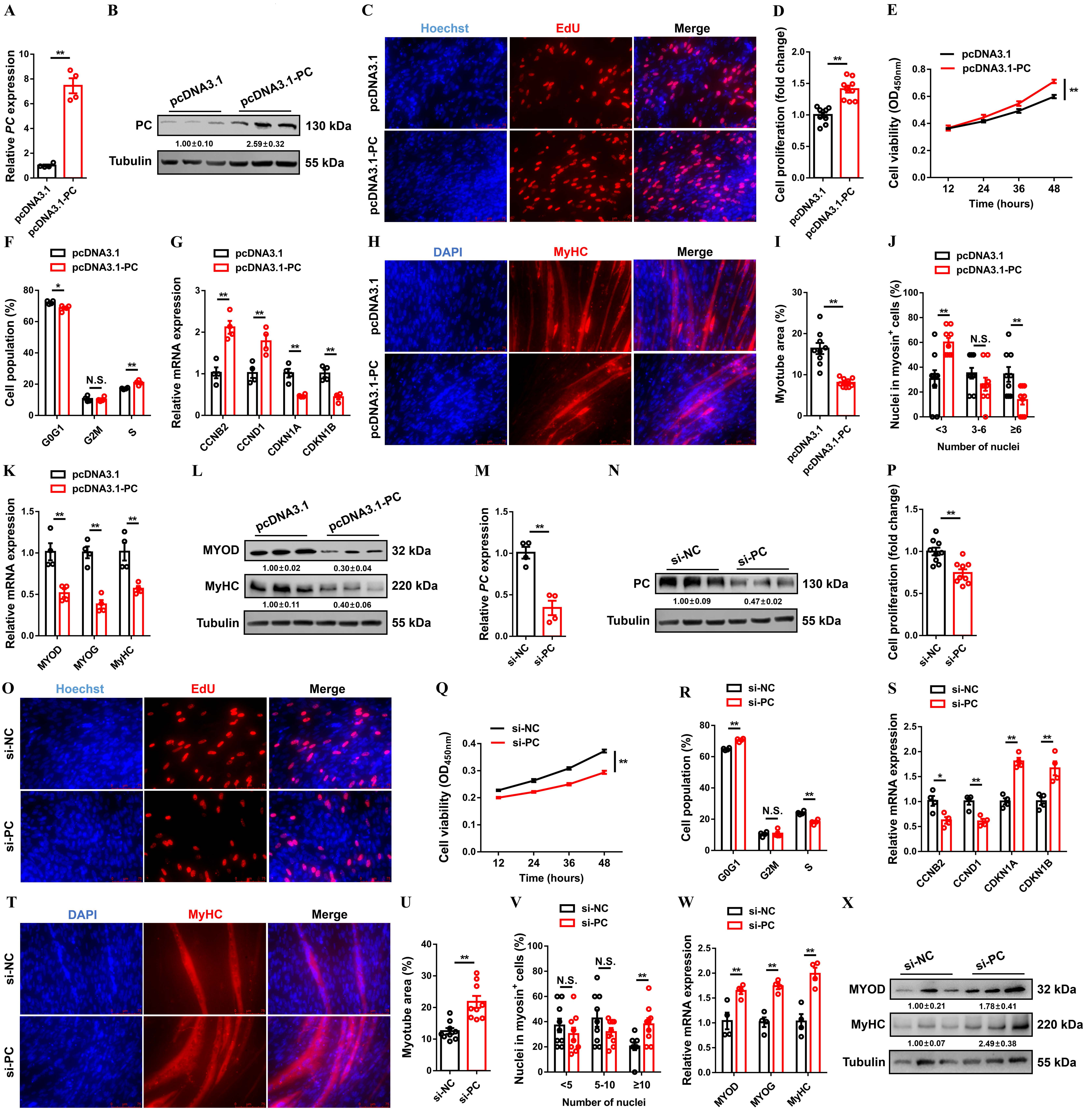

A
B
C
D
E
Relative PC expression
pcDNA3.1
pcDNA3.1-PC
PC
130 kDa
Tubulin
55 kDa
1.00±0.10
2.59±0.32
Hoechst
EdU
Merge
Cell proliferation (fold change)
Cell viability (OD450nm)
Time (hours)
F
G
H
I
J
Cell population (%)
G0G1
G2M
S
N.S.
Relative mRNA expression
CCNB2
CCND1
CDKN1A
CDKN1B
DAPI
MyHC
Myotube area (%)
Nuclei in myosin+ cells (%)
Number of nuclei
K
L
M
N
P
MYOD
MYOG
32 kDa
220 kDa
1.00±0.02
0.30±0.04
1.00±0.11
0.40±0.06
si-NC
si-PC
1.00±0.09
0.47±0.02
O
Q
R
S
T
U
V
W
X
1.00±0.21
1.78±0.41
1.00±0.07
2.49±0.38

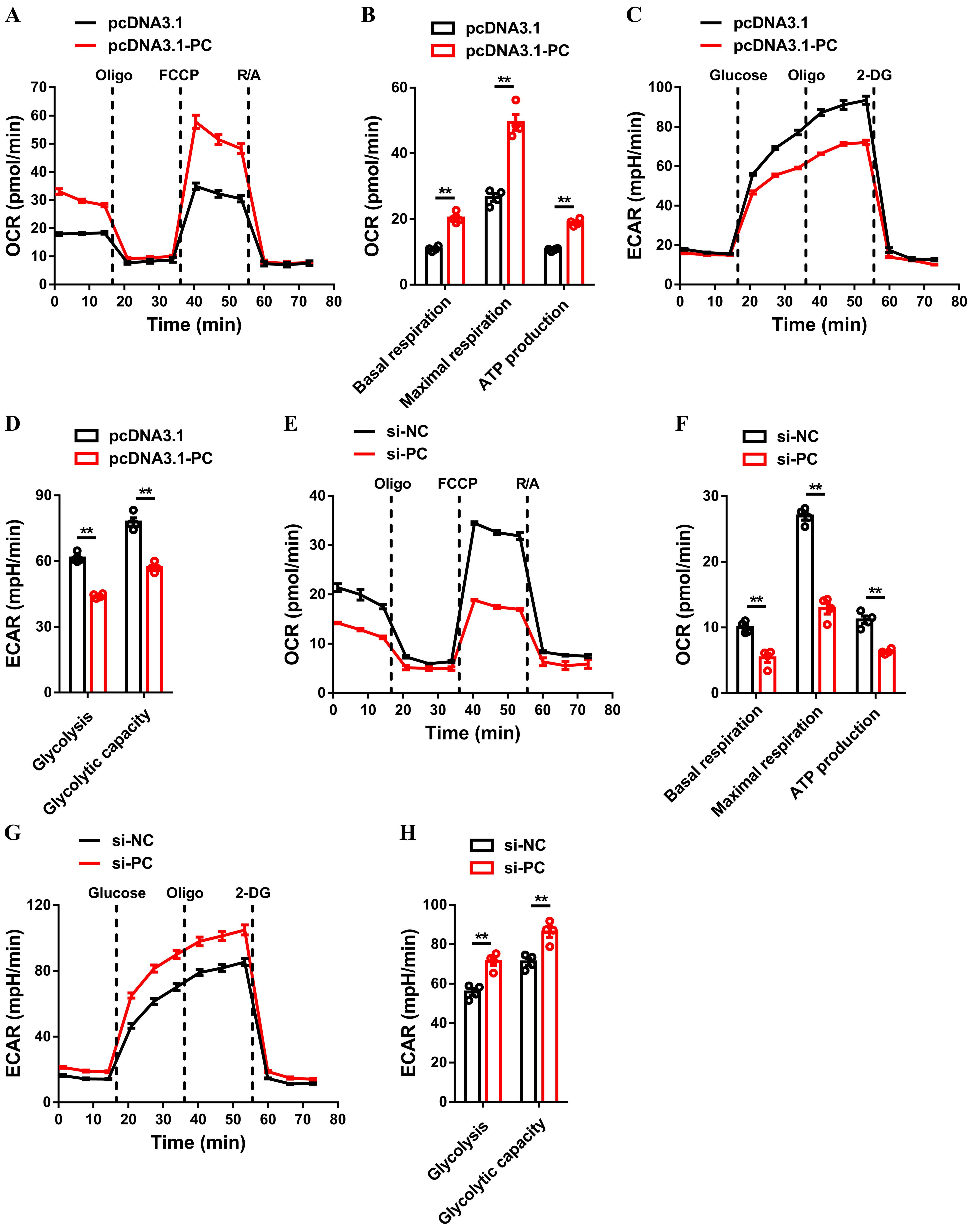
A
pcDNA3.1
pcDNA3.1-PC
Oligo
FCCP
R/A
OCR (pmol/min)
Time (min)
B
pcDNA3.1
pcDNA3.1-PC
OCR (pmol/min)
Basal respiration
Maximal respiration
ATP production
C
pcDNA3.1
pcDNA3.1-PC
Glucose
Oligo
2-DG
ECAR (mpH/min)
Time (min)
D
pcDNA3.1
pcDNA3.1-PC
ECAR (mpH/min)
Glycolysis
Glycolytic capacity
E
si-NC
si-PC
Oligo
FCCP
R/A
OCR (pmol/min)
Time (min)
F
si-NC
si-PC
OCR (pmol/min)
Basal respiration
Maximal respiration
ATP production
G
si-NC
si-PC
Glucose
Oligo
2-DG
ECAR (mpH/min)
Time (min)
H
si-NC
si-PC
ECAR (mpH/min)
Glycolysis
Glycolytic capacity

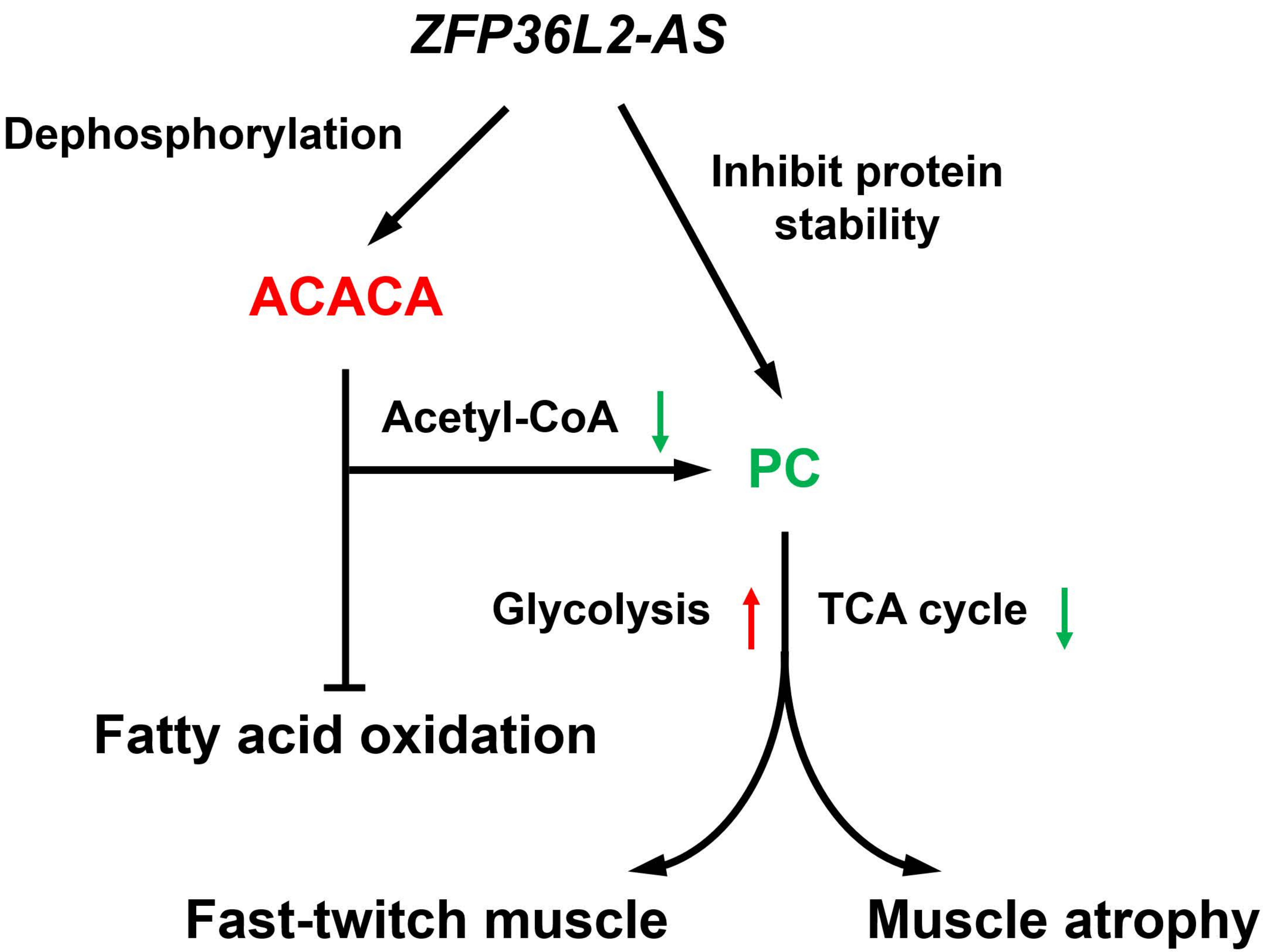
ZFP36L2-AS
Dephosphorylation
Inhibit protein stability
ACACA
Acetyl-CoA
PC
Glycolysis
TCA cycle
Fatty acid oxidation
Fast-twitch muscle
Muscle atrophy
