# Supplementary material for: Long noncoding RNA ZFP36L2-AS functions as a metabolic modulator to regulate muscle development
Source: Cell Death Dis. 2022 Apr 21;13(4):389. doi: 10.1038/s41419-022-04772-2 (PMC9023450; doi:10.1038/s41419-022-04772-2)
Supplement: Supplementary file 3 — Supplementary Information [file 41419_2022_4772_MOESM3_ESM.docx]

**Supplementary Table 1.** The full-length sequence of lncRNA *ZFP36L2-AS*. Coordinates are listed according to GRCg6a reference, Annotation Release 104 (chromosome 3: 25089365 to 25092829).

ACAGGGTGCAAAAGGACGGGCCTGCGAGTGCGGGGTGGCTGGGCCCGCCGTCCTACATGCAGCCCTGGCTTTAGGGGAGCGGTCACCAGGCCATCCCGGCGGCCTCCCCTACACGGCAGGCGGAATGTAGGGCAGCAGCTCGGGGTGCAGCCTGTACCAGGCCCTGCGCCATCACACTGAGCCAGGGCCGCCAGGAGGCTGCAGGGAGGGCATGGAGCTGGTAGATGTCACTGTCCCACATATACCGTGTGCAGCAGCACGGGAGGTCTTGATCTGCTTCAGAGAGAATAAGGGAGTGTCGGGGAAGAGGTGGGCTAAAGCAAGAAGTGCACCTTGGGAAAGAAAAAAGCCCTCAACTCAACGAGGTGTGCACTCCCAGCTAACGCAGTTATAGCTCAACAAGTTGTTTCCAAATCCCAAAAGCGAGTCAGGACATTGGCCTGGGCGAGCTGCAACCACAGCAGGGCCCCTCCTGCGGGCACCGGGGGGCCCTCAGAGCAGCTCCTCCAGAACTGCTAAGGAAGCAATCCCCAGCAAATCCCATCAGGCTAACCGGTAAAAAAGGGCCAATCCCAAGCAAAACCGATCGGACTGACCCAATGAAAAAGGCTGGCTGCTGTGGCAGGGCTGTGTTGTCATACATTGCTTACGTTTGTTTTAATTTTAAACCCAGAGTCAGCCCAAGGTTTGTTACACAAACATATGGCACCCGTATTACATTCTGCCTCGTATTTATAGAAAGTACCGCGGCAGTCCAGACAGCGCAGAGATGCCATAACGTTAACAACATTTATTATTAGTATGACAAAAGAACAAAAATAAGCCTGTAGAAACCACGACTTAAAGTTTAAGGGATTGATTGGAATAAGGAACCGAACTGTGAAGCTATTAGTGAAGAAAATCCCTCCAGCATTAACACACGCAATCCCCAGGCCACCGAGCGTTTTGTTATATAAACACGCCCCCAGAAAGGCAATTTCTTTAAAAACTCAAGCCCTCGGTGCTGATTAACACATTTAATGCCAGCCCGATTAGCACTGCTGAGCCCCAAGCCCAGGGAGCGCTGAGGCAGAAGTCCTCATCCCTCTCTCAGTCCCGCTGCTGAGGAGAGCGGATCTGGGACAAAGGGTGAATCTTAACGATGCCTTTTTATTGCCCAAACTGCCACTTCCTCCTCAGCCCAAACAAGCAGGGAAGGGTACCAGCACCTCCCCGCCAGCGATAAATGCCATTACTAGATTTTCCCATTCCAGCTGCAAAAGGTGCACGCAGCAGAGGCCTCATCCTCTACCCTCGGCCTAAAAAGAAAGTGGGAAGAAAAAAGAAGAGGGAAGCAAGCAGGATAGAGCACCCCAGCAGCACTCGCGGGGCCGAACCTTCTAGAAGAACATATTAGCTTTGGCGGCTGCCCAGCACTGGCTGCAGGAGCACGCTGGTTAATCTGTAGCCAGACTGAGGGCGGTGGGGAGCTGGACAGGGCCACCCCGCAGGCAGGGAAGGGTGAGGGGTGGGGTGGGACGGGCTTGTTGCTTATGGGTTTCTTTCTTTTTTTTTTTTTTTTTCCAAAAAAATTTTATTGGGAGAACTACAAAACATTTACAGTACAAAGTTTACAGTCTCACACAATTTGTAGTGAACTGACTCCCGAAAAATATATTACAACTCAAGTTGACTTATCCTTTAGTTACATTCAAAACATACTTCTGTTAAAGTAGTCCAAAAAGAGTACATAGTGCTCAATCTGTACCTATGTACAAACAAAACTAAGCTACCGCTCATTCATCCACCGTCCAGGAAAGTTTTGGAAAACTCCCGACTTTCTACGCAAGTAAAAAAAAAATAATAATAATATCACAAGTTTTGGAATTCAATTAAACAAGATATGCTCGTGTATAAAGTTTCAGATCCAAAGATGGCCGCCATTCTTACAAAATAAAGAGTGGTGAAATTTTGCTGTAGTCAAAAACCCATCCCTACATTCAAATTATCACCTCAAGCATTAAGAGAAATACTCATTTGGTTGAGAAGTATTTAAGGCAAGCGTGGACCCTCCAGAAGGCACTGTGAGGCATAATACTGGGCACCCCAATTATTGCTACATCCGTTGAGACTACATCACAGTGTGAGTGGTGGAGTTAGGCAACGAGGAACACTTTTTGGTTACACTTGGAACGAGGCTGCGTCAGGTTCGCCACTTTGAAGTCTGATTGCCAAAAGTCAGTCTCCCGTTGTTGGTTTTCTTCTACTCGGACGGGCTACAACCATTTACATTCTACAAAAACCCCATAGAAATTCCTCAAACTACTTCCACAGCATCGAGACCGATTTCTGTACAGAAACCATGCAATCTTTCAGATTTACGTAAACAAGGAAAGAAATTAACGAAATAAATATTACATACAATCTCTTAAATTAAGAATTTTTACTCATTTACAATAAAATAACCAAGTGAAGTTACAAAAAGGCATATATTACTGTGAAAAGAACACACTCCACGTTTCGCCGATTAATAATGGCAATCATAATTTAAACATAATAAAAGAATATATATCTATTGCTTTTCATCATACCCGATAAATACAGTATGAACAAATTACCAATGCATACTTTTCACGAGATAATAAATAAGTTAAATAGTTTCATGTTGAGTTGTGTGCAGTGACTCATTCATGCAATCAACTCAAAACAGCTAAAAAAAAAAACAATTAAAAAAAATCAGCAGTTATTCTCCAACAATTACAAACTAAACTCGGTCGAGTGCTTCCAAATAAAGCAACAACGAAAAAAAGAAAAGAGTTGTTCTAAGCCAAACATCCACTAAGTGGTGGCAATGGAACCAATATTAAACTTTGGAATGAAGGTTTTAGCATTTGTTATAATAATAATATATAGATATATAAAAAAAATAGAAGGTTGTTATCAAGGCATATTCTTGGCAGGATGTTGGATTTCGCGCTGGAGAAGCTCGCGGTGGGTCCGGCCGCGTTCCCGGATGGAATGGTGCTGCTGGTGCGGATCAGAACTCGGCTCCGGAGAACGAGAAGGACAACCCCGAAACACAAATCCCCCTTCTTCCAAGGTCTCTCTAGAAAAATAGGTTTGTTCAGTTCACGTCAGTCGCCCTTTCGCAAGACGCTTTTCGCTTTGTTCAGCTCTCGTGTGTACATGGATAGGTTACGCGTATAAAAAAAGACACGACAGGAGACGGACAGAGAGGGGGGCCGGCTCGCTCCCCGCGCCCCACCGCCCGCGGCCCCCTCCTTCCATCCTTCCATCCTTCAGTCGTCGGAGATGGAGAGGCGGCTGAAGATGGGCAGGCGGCGGCCCGAGTCCAGGCCGGGCGACTCGGAGCCGCTGAGCGAGCCGGAGCTGAGCGAGCCGCTCAGGTAGCTCTCGCGGTCCGAGAGCGAGTCCGGCGGGCTGGGCGGCGCGTCGAACACGGGCGACTCGGAAAGGCGGCGCAGG

**Supplementary Table 2.** Comparative metabolome analysis of control group versus lncRNA *ZFP36L2-AS* knockdown gastrocnemius.

Separate Excel file.

**Supplementary Table 3.** lncRNA *ZFP36L2-AS* specific binding proteins identified by RNA pull-down coupled to mass spectrometry.

Separate Excel file.

**Supplementary Table 4.** Information of Primers.

| **Primer name** | **Primer sequences (5’ to 3’)** | **Usage** |
| --- | --- | --- |
| qPCR-ZFP36L2-AS | F: ACTGTGAGGCATAATACTGGG | qPCR |
|  | R: AAAGTGGCGAACCTGACG |  |
| qPCR-ACACA | F: CCCTGTTTCTGTCTGCTC | qPCR |
|  | R: TCAGGACCTTCTCAATAACTC |  |
| qPCR-PC | F: TCCTGCAAGGCTACATCGG | qPCR |
|  | R: CACCTCAAACTCCTCAGCGATA |  |
| qPCR-CCNB2 | F: CAGTAAAGGCTACGAAAG | qPCR |
|  | R: ACATCCATAGGGACAGG |  |
| qPCR-CCND1 | F: CAGAAGTGCGAAGAGGAAGT | qPCR |
|  | R: CTGATGGAGTTGTCGGTGTA |  |
| qPCR-CDKN1A | F: CCCGTAGACCACGAGCAGAT | qPCR |
|  | R: CGTCTCGGTCTCGAAGTTGA |  |
| qPCR-CDKN1B | F: TCGCTGTGCTGGGCTGAA | qPCR |
|  | R: CAAGGACGAAAGGATGTGGG |  |
| qPCR-MYOD | F: GCTACTACACGGAATCACCAAAT | qPCR |
|  | R: CTGGGCTCCACTGTCACTCA |  |
| qPCR-MYOG | F: CGGAGGCTGAAGAAGGTGAA | qPCR |
|  | R: CGGTCCTCTGCCTGGTCAT |  |
| qPCR-MyHC | F: CTCCTCACGCTTTGGTAA | qPCR |
|  | R: TGATAGTCGTATGGGTTGGT |  |
| qPCR-COX2 | F: GTAGATGCCCAAGAAGTT | qPCR |
|  | R: GTTTGATTTAGTCGTCCAG |  |
| qPCR-β-globin | F: CAGCCAGGTGGAGGATTT | qPCR |
|  | R: GAATAGGAGGACCCTCTGTTAG |  |
| qPCR-CPT1 | F: GCTTATTGTAGTTGTGGGTG | qPCR |
|  | R: AAAGTTTGCCGTGTTCAG |  |
| qPCR-FASN | F: CGCAGGCATAGCAGGAAA | qPCR |
|  | R: CCAAAGAAGGAGGCATCAA |  |
| qPCR-MACT | F: GTGCTGGGCTACGACCTGCT | qPCR |
|  | R: CAGTGCCTACGGGCTTCCAG |  |
| qPCR-OXSM | F: ACATAACAGCACCTAATCC | qPCR |
|  | R: ATGGGACGTAATTGAGAT |  |
| qPCR-HK1 | F: CTGGATCTCGGTGGTTCTTAC | qPCR |
|  | R: TTGTCGGCACGGGAAAGA |  |
| qPCR-GPI | F: ATTCACTTTGGGAGCAATC | qPCR |
|  | R: ACTCCAACTCTGGCTCAAT |  |
| qPCR-PGAM1 | F: GCGAGGCTCAGGTGAAGAT | qPCR |
|  | R: GTCCTCCGTCAGGTCAGC |  |
| qPCR-PGK1 | F: CCCTGGATAAGGTGGATG | qPCR |
|  | R: TTGTCAGGCATGGGAACT |  |
| qPCR-PYGL | F: ACATTTGCCTACACGAACC | qPCR |
|  | R: TGCCTCCCTCCTCTATCA |  |
| qPCR-SOX6 | F: TCAGGTTCAGGGTCACATGCC | qPCR |
|  | R: TTGCTGGAGCTGTAAAGGGC |  |
| qPCR-TNNC1 | F: GTTGAGCAGTTGACAGAAGA | qPCR |
|  | R: GAACCATCATAACAAGGAAC |  |
| qPCR-TNNC2 | F: GAGCAGCAAAGATGGCGTCA | qPCR |
|  | R: ATCACCGTGCCCAACTCCTT |  |
| qPCR-TNNI1 | F: GAGGAGTGGGAGCAGGAGAT | qPCR |
|  | R: TTCGTCCACAATCTCAACCT |  |
| qPCR-TNNT1 | F: GAGCCGCACGGAGAAGGAGC | qPCR |
|  | R: CCCGAAGTGGGGCATGTTGG |  |
| qPCR-TNNT3 | F: AGAGGGAAGAAGCAAACAGC | qPCR |
|  | R: GTCCCACAGTTCCTTAGCCT |  |
| qPCR-ATROGIN1 | F: TCAACGGGTCGGCAAGTCT | qPCR |
|  | R: TCCCTCCCATCGCTCAGTC |  |
| qPCR-MURF1 | F: GGACGAGCGGATCAACAT | qPCR |
|  | R: GGGAGATGATGGTCTGGATG |  |
| qPCR-LC3B | F: GAGCAAAGAGTTGAAGATG | qPCR |
|  | R: GTCCTAGACGGAAGATTG |  |
| qPCR-SQSTM1 | F: AGCGACGAGGAGCTGGATC | qPCR |
|  | R: CCTTGTGGATGCCTTTACCC |  |
| qPCR-ULK1 | F: TCGTTGCCTTGTATGACTT | qPCR |
|  | R: TTTATGCGAATGTTGTTGG |  |
| qPCR-β-actin | F: GATATTGCTGCGCTCGTTG | qPCR |
|  | R: TTCAGGGTCAGGATACCTCTTT |  |
| 5’ RACE-ZFP36L2-AS | Outer: TGGTTGCAGCTCGCCCAGGCCAAT | RACE |
|  | Inner: GCCAATGTCCTGACTCGCTTTTG |  |
| 3’ RACE- ZFP36L2-AS | Outer: CACCGCCCGCGGCCCCCTCCTTC | RACE |
|  | Inner: TTCAGTCGTCGGAGATGGAGAGG |  |
| ZFP36L2-AS-ORF1-3xFLAG | F: **AAGCTT**ATGGAGCTGGTAGATGTCACT | Vector construction |
|  | R: **CTCGAG**TAACTGCGTTAGCTGGGAG |  |
| ZFP36L2-AS-ORF2-3xFLAG | F: **AAGCTT**ATGAAAAAGGCTGGCTG | Vector construction |
|  | R: **CTCGAG**ATAATAAATGTTGTTAACG |  |
| ZFP36L2-AS-ORF3-3xFLAG | F: **AAGCTT**ATGCCTTTTTATTGCCCA | Vector construction |
|  | R: **CTCGAG**TCGCTGGCGGGGAGGTG |  |
| ZFP36L2-AS-ORF4-3xFLAG | F: **AAGCTT**ATGCCATTACTAGATTTTCC | Vector construction |
|  | R: **CTCGAG**ATATGTTCTTCTAGAAGGTTCG |  |
| ZFP36L2-AS-ORF5-3xFLAG | F: **AAGCTT**ATGGGTTTCTTTCTTTTT | Vector construction |
|  | R: **CTCGAG**CTTTAACAGAAGTATGTTTTG |  |
| ZFP36L2-AS-ORF6-3xFLAG | F: **AAGCTT**ATGAACAAATTACCAATGC | Vector construction |
|  | R: **CTCGAG**CTGCACACAACTCAACATG |  |
| ZFP36L2-AS-ORF7-3xFLAG | F: **AAGCTT**ATGGAACCAATATTAAACT | Vector construction |
|  | R: **CTCGAG**TTTTTCTAGAGAGACCTTG |  |
| ZFP36L2-AS-ORF8-3xFLAG | F: **AAGCTT**ATGAAGGTTTTAGCATTTG | Vector construction |
|  | R: **CTCGAG**TTTTTCTAGAGAGACCTTGG |  |
| ZFP36L2-AS-ORF9-3xFLAG | F: **AAGCTT**ATGTTGGATTTCGCGCT | Vector construction |
|  | R: **CTCGAG**TACGCGTAACCTATCCATGTA |  |
| ZFP36L2-AS-ORF10-3xFLAG | F: **AAGCTT**ATGGAATGGTGCTGCTGG | Vector construction |
|  | R: **CTCGAG**TTTTTCTAGAGAGACCTTGGAAG |  |
| ZFP36L2-AS-ORF11-3xFLAG | F: **AAGCTT**ATGGTGCTGCTGGTGCG | Vector construction |
|  | R: **CTCGAG**TACGCGTAACCTATCCATGTAC |  |
| ZFP36L2-AS-ORF12-3xFLAG | F: **AAGCTT**ATGGAGAGGCGGCTGAAGAT | Vector construction |
|  | R: **CTCGAG**CCTGAGCGGCTCGCTCA |  |
| ZFP36L2-AS-ORF13-3xFLAG | F: **AAGCTT**ATGGGCAGGCGGCGGC | Vector construction |
|  | R: **CTCGAG**CCTGAGCGGCTCGCTCAGCT |  |
| pcDNA3.1- ZFP36L2-AS | F: **AAGCTT**ACAGGGTGCAAAAGGACGGGC | Vector construction |
|  | R: **GCTAGC**ATGTCGCAGCTGTGTGTCCCC |  |
| pcDNA3.1-PC | F: **GCTAGC**ATGAACTACATAAACTCAAAAT | Vector construction |
|  | R: **AAGCTT**TTACTCGATCTCGGCGATGAGG |  |
| pDC316-mCMV-ZsGreen-ZFP36L2-AS | F: **GCTAGC**ACAGGGTGCAAAAGGACGGGCCTGCGAG | Vector construction |
|  | R: **AAGCTT**CCTGCGCCGCCTTTCCGAGTCGCCCGTGT |  |
| pLVX-shRNA2-Puro-ZFP36L2-AS | F: **GATCC**CCCGATAAATACAGTATGA**TTCAAGAGA**TCATACTGTATTTATCGGGTTTTTT**G** | Vector construction |
|  | R: **AATTC**AAAAAACCCGATAAATACAGTATGA**TCTCTTGAA**TCATACTGTATTTATCGGG**G** |  |

Sequences in bold represent the enzyme cutting sites.

**Supplementary Table 5.** Oligonucleotide sequences in this study.

| **Fragment name** | **Sequences (5’ to 3’)** |
| --- | --- |
| si-ZFP36L2-AS | CCCGATAAATACAGTATGA |
| ASO-ZFP36L2-AS | AGTCAAAAACCCATCCCTAC |
| si-ACACA | ACGTATCACCAGTGAGAAT |
| si-PC | TCGAATTCCTGCAAGGCTA |

**Supplementary Figure 1**. Characterization of the lncRNA *ZFP26L2-AS*. (**A**) Conservative analysis of *ZFP26L2-AS* performed by using the NCBI’s BLAST. A total of eighteen species, including *Anas platyrhynchos*, *Anser cygnoides*, *Apteryx mantelli mantelli*, *Aquila chrysaetos*, *Bos taurus*, *Coturnix japonica*, *Gallus gallus*, *Geospiza fortis*, *Homo sapiens*, *Meleagris gallopavo*, *Melopsittacus undulatus*, *Mus musculus*, *Numida meleagris*, *Ovis aries*, *Pan troglodytes*, *Rattus norvegicus*, *Sus scrofa* and *Zebra finch* were used for Nucleotide BLAST. Top 5 most conservative results were listed above. (**B**) Relative *ZFP26L2-AS* expression in muscle-resident cells.

**Supplementary Figure 2**. Overexpression of *ZFP26L2-AS* inhibits myoblast proliferation but promotes myogenic differentiation. (**A**-**K**) Relative *ZFP26L2-AS* expression (**A**), EdU proliferation assays (**B**), proliferation rate of myoblasts (**C**), CCK-8 assays (**D**), cell cycle analysis (**E**), relative mRNA levels of several cell cycle genes (**F**), MyHC immunostaining (**G**), myotube area (**H**), myoblast fusion index (**I**) and relative mRNA (**J**) and protein (**K**) expression levels of myoblast differentiation marker genes with *ZFP26L2-AS* overexpression *in vitro*. In panel (**K**), the numbers shown below the bands were folds of band intensities relative to control. Band intensities were quantified by ImageJ and normalized to β-Tubulin. Data are expressed as a fold-change relative to the control. Results are presented as mean ± SEM. In panels (**A**, **C**-**F**, and **H**-**J**), statistical significance of differences between means was assessed using independent sample *t*-test.

**Supplementary Figure 3**. Overexpression of *ZFP26L2-AS* represses cellular respiration and fatty acid oxidation in skeletal muscle. (**A**-**E**) OCR (**A**), basal respiration, maximal respiration and ATP production (**B**), ECAR (**C**), glycolysis and glycolytic capacity (**D**), and relative cellular ATP content (**E**) with *ZFP26L2-AS* overexpression in CPMs. (**F**-**K**) Relative *ZFP26L2-AS* expression (**F**), relative mtDNA content (**G**), relative fatty acid β-oxidation rate (**H**), relative mRNA (**I**) and protein (**J**) expression levels of fatty acid oxidation or synthesis related-genes, and relative FFA and TG content (**K**) in gastrocnemius with *ZFP26L2-AS* overexpression *in vivo*. In panel (**J**), the numbers shown below the bands were folds of band intensities relative to control. Band intensities were quantified by ImageJ and normalized to β-Tubulin. Data are expressed as a fold-change relative to the control. Results are shown as mean ± SEM. In panels (**B**, **D**-**I** and **K**), statistical significance of differences between means was assessed using independent sample *t*-test.

**Supplementary Figure 4**. *ZFP36L2-AS* does not regulate cellular ATP content in satellite cells. (**A** and **B**) Relative cellular ATP content with *ZFP26L2-AS* interference (**A**) and overexpression (**B**) in satellite cells. Results are shown as mean ± SEM. In all panels, statistical significance of differences between means was assessed using independent sample *t*-test.

**Supplementary Figure 5**. Overexpression of *ZFP36L2-AS* activates fast-twitch muscle phenotype and induces muscle atrophy. (**A**-**L**) Relative glycogen content (**A**), relative mRNA expression levels of glycogenolytic and glycolytic genes (**B**), relative enzymes activity of LDH and SDH (**C**), immunohistochemistry analysis of MYH1/MYH7 (**D**), MYH1/MYH7 protein content (**E**), relative mRNA expression levels of several fast-/slow-twitch myofiber genes (**F**), relative gastrocnemius muscle weight (**G**), H&E staining (**H**) and frequency distribution of fiber CSA (**I**), relative mRNA expression of the atrophy and autophagy-related genes (**J**), and the protein expression levels of mTOR signaling in gastrocnemius with *ZFP36L2-AS* overexpression *in vivo*. In panel (**J**), the numbers shown below the bands were folds of band intensities relative to control. Band intensities were quantified by ImageJ and normalized to β-Tubulin. Data are expressed as a fold-change relative to the control. Results are shown as mean ± SEM. In panels (**A**-**C**, **E**-**G**, and **J**), statistical significance of differences between means was assessed using independent sample *t*-test.

**Supplementary Figure 6**. GO functions and KEGG pathways analysis of lncRNA *ZFP36L2-AS* specific binding proteins identified by RNA pull-down coupled to mass spectrometry. (**A**) GO functions analysis of lncRNA *ZFP36L2-AS* specific binding proteins identified by RNA pull-down coupled to mass spectrometry. (**B**) KEGG pathways analysis of lncRNA *ZFP36L2-AS* specific binding proteins identified by RNA pull-down coupled to mass spectrometry.

**Supplementary Figure 7**. lncRNA *ZFP36L2-AS* 816-1785 region is necessary for *ZFP36L2-AS* to function. (**A**-**K**) Relative *ZFP26L2-AS* 816-1785 region expression (**A**), relative mRNA levels of several cell cycle genes (**B**), relative mRNA expression levels of myoblast differentiation marker genes (**C**), relative mRNA expression levels of fatty acid oxidation or synthesis related-genes (**D**), relative mRNA expression levels of several fast-/slow-twitch myofiber genes (**E**), relative mRNA expression of the atrophy and autophagy-related genes (**F**), the protein expression levels of ACACA and phosphorylated ACACA (**G**), relative ACACA activity (**H**), the protein expression level of PC (**I**), relative PC activity (**J**), and relative acetyl-CoA content (**K**) with *ZFP26L2-AS* 816-1785 region overexpression *in vitro*. In panels (**G** and **I**), the numbers shown below the bands were folds of band intensities relative to control. Band intensities were quantified by ImageJ and normalized to β-Tubulin. Data are expressed as a fold-change relative to the control. Results are presented as mean ± SEM. In panels (**A**-**F**, **H**, **J** and **K**), statistical significance of differences between means was assessed using independent sample *t*-test.

**Supplementary Figure 8**. The mRNA level of *ACACA* and *PC* didn’t change with *ZFP36L2-AS* overexpression and knockdown both *in vitro* and *in vivo*. (**A** and **B**) The mRNA level of *ACACA* with *ZFP36L2-AS* overexpression and knockdown *in vitro* (**A**) and *in vivo* (**B**). (**C** and **D**) The mRNA level of *PC* with *ZFP36L2-AS* overexpression and knockdown *in vitro* (**C**) and *in vivo* (**D**). Results are shown as mean ± SEM. In all panels, statistical significance of differences between means was assessed using independent sample *t*-test.

**Supplementary Figure 9**. Interference of *ACACA* promotes myoblast proliferation and inhibits myoblast differentiation. (**A**-**L**) Relative mRNA (**A**) and protein (**B**) expression levels of *ACACA*, EdU proliferation assays (**C**), proliferation rate of myoblasts (**D**), CCK-8 assays (**E**), cell cycle analysis (**F**), relative mRNA levels of several cell cycle genes (**G**), MyHC immunostaining (**H**), myotube area (**I**), myoblast fusion index (**J**) and relative mRNA (**K**) and protein (**L**) expression levels of myoblast differentiation marker genes with *ACACA* interference *in vitro*. In panels (**B** and **L**), the numbers shown below the bands were folds of band intensities relative to control. Band intensities were quantified by ImageJ and normalized to β-Tubulin. Data are expressed as a fold-change relative to the control. Results are presented as mean ± SEM. In panels (**A**, **D**-**G**, and **I**-**K**), statistical significance of differences between means was assessed using independent sample *t*-test.

**Supplementary Figure 10**. The expression and location analysis of *ACACA* and *PC*. (**A** and **D**) Tissue expression proﬁles of *ACACA* (**A**) and *PC* (**D**). The horizontal axis and vertical axis indicate different tissues and their relative expression values, respectively. (**B** and **E**) Relative *ACACA* (**B**) and *PC* (**E**) expression during CPM proliferation and differentiation. (**C** and **F**) Subcellular location of ACACA and PC protein annotated by UniProt Knowledgebase (https://www.uniprot.org/). In panels (**A**, **B**, **D**, and **E**), results are presented as mean ± SEM.

**Supplementary Figure 11**. *PC* promotes myoblast proliferation and inhibits myoblast differentiation. (**A**-**X**) Relative mRNA (**A** or **M**) and protein (**B** or **N**) expression levels of *PC*, EdU proliferation assays (**C** or **O**), proliferation rate of myoblasts (**D** or **P**), CCK-8 assays (**E** or **Q**), cell cycle analysis (**F** or **R**), relative mRNA levels of several cell cycle genes (**G** or **S**), MyHC immunostaining (**H** or **T**), myotube area (**I** or **U**), myoblast fusion index (**J** or **V**) and relative mRNA (**K** or **W**) and protein (**L** or **X**) expression levels of myoblast differentiation marker genes with *PC* overexpression or interference *in vitro*. In panels (**B**, **L**, **N**, and **X**), the numbers shown below the bands were folds of band intensities relative to control. Band intensities were quantified by ImageJ and normalized to β-Tubulin. Data are expressed as a fold-change relative to the control. Results are presented as mean ± SEM. In panels (**A**, **D**-**G**, **I**-**K**, **M**, **P**-**S** and **U**-**W**), statistical significance of differences between means was assessed using independent sample *t*-test.

**Supplementary Figure 12**. *PC* promotes cellular mitochondrial respiration and decreases glycolytic capacity. (**A**-**H**) OCR (**A** or **E**), basal respiration, maximal respiration and ATP production (**B** or **F**), ECAR (**C** or **G**), and glycolysis and glycolytic capacity (**D** or **H**) of CPMs with *PC* overexpression or interference. Results are shown as mean ± SEM. In all panels, statistical significance of differences between means was assessed using independent sample *t*-test.

**Supplementary Figure 13**. Model of lncRNA *ZFP36L2-AS* interacts with ACACA and PC to facilitate intramuscular fat deposition, as well as activate fast-twitch muscle phenotype and induce muscle atrophy.
