# Supplementary figures and images for: Long noncoding RNA ZFP36L2-AS functions as a metabolic modulator to regulate muscle development
Source: Cell Death Dis. 2022 Apr 21;13(4):389. doi: 10.1038/s41419-022-04772-2 (PMC9023450; doi:10.1038/s41419-022-04772-2)

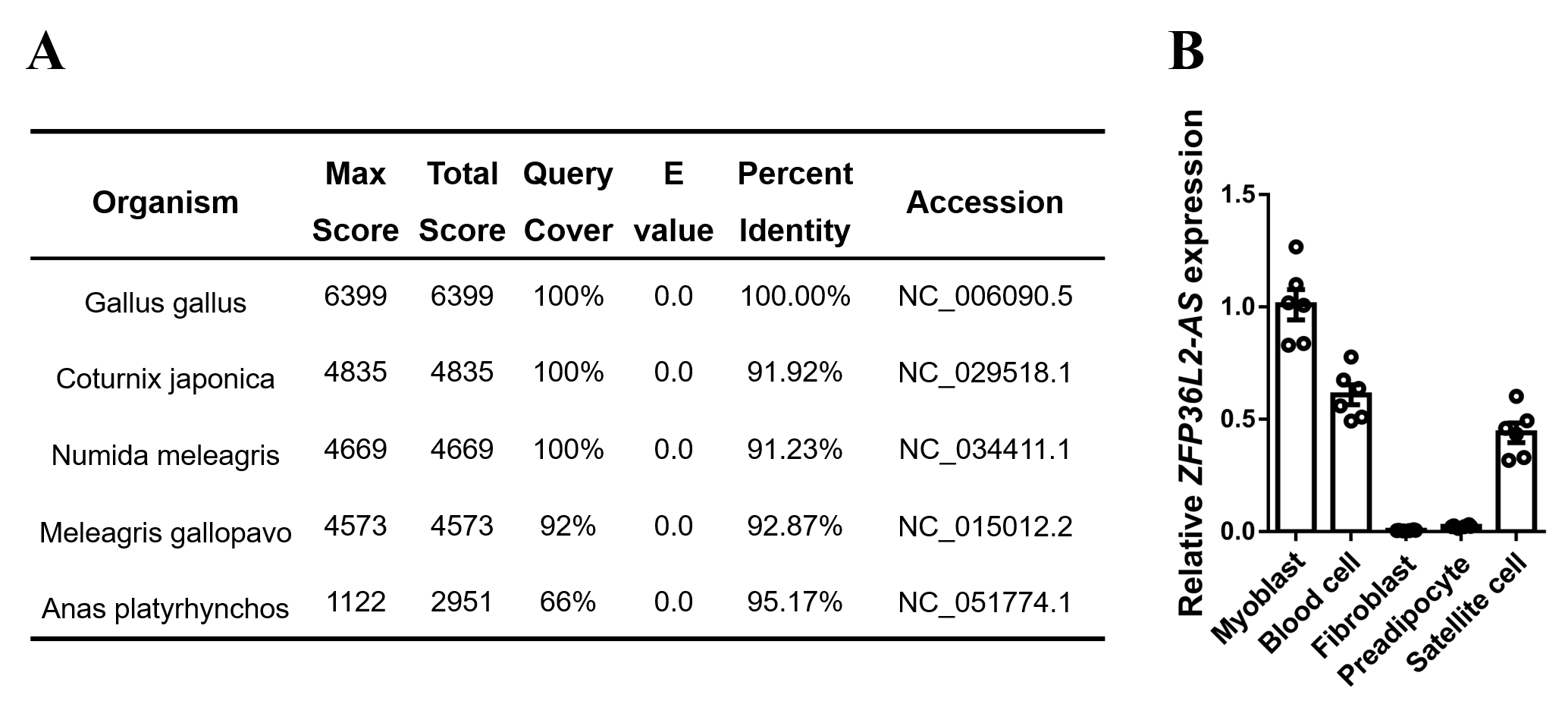

Supplement: Supplementary file 6 — Supplementary Figure 1 [file 41419_2022_4772_MOESM6_ESM.tif]

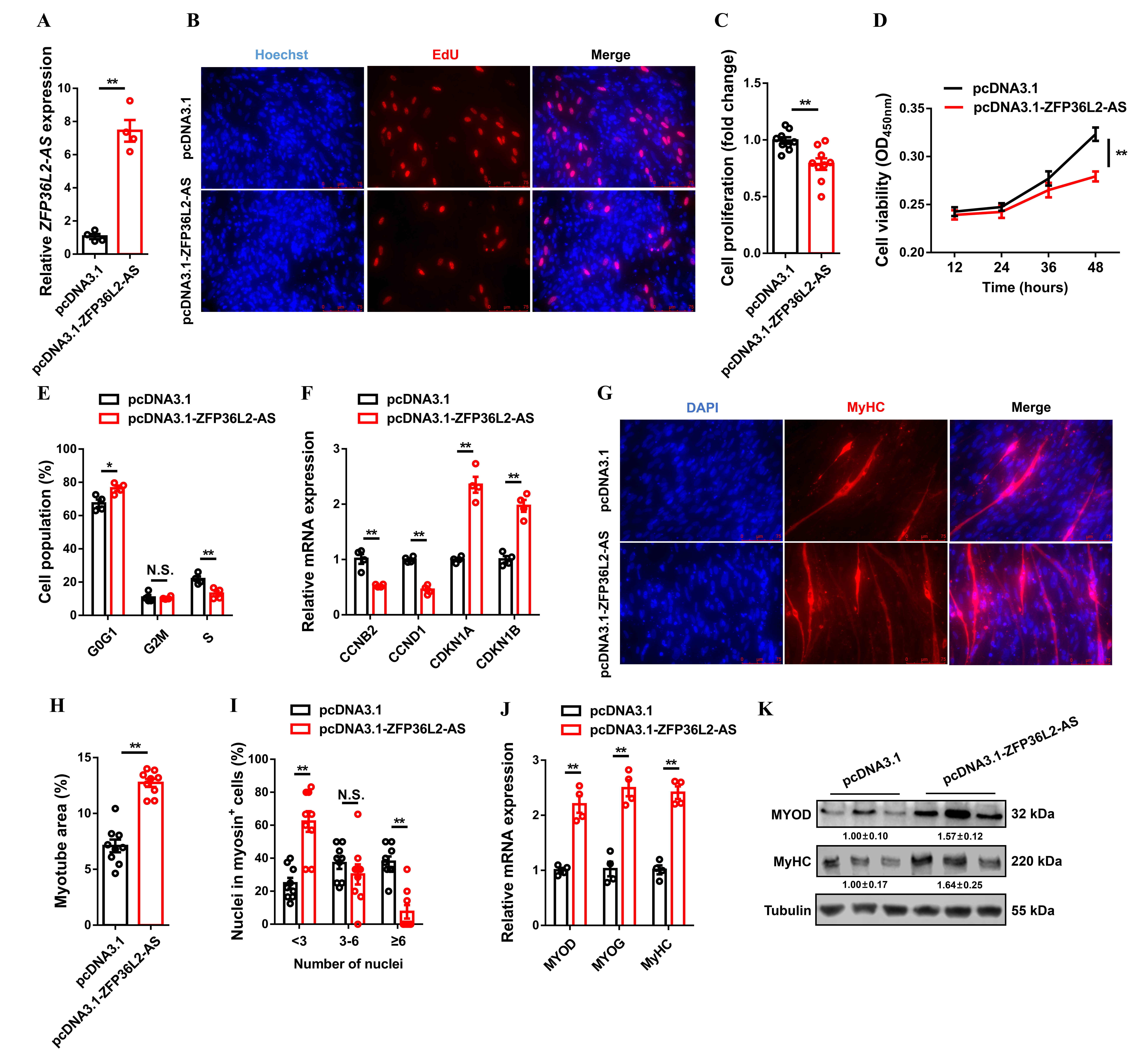

Supplement: Supplementary file 7 — Supplementary Figure 2 [file 41419_2022_4772_MOESM7_ESM.tif]

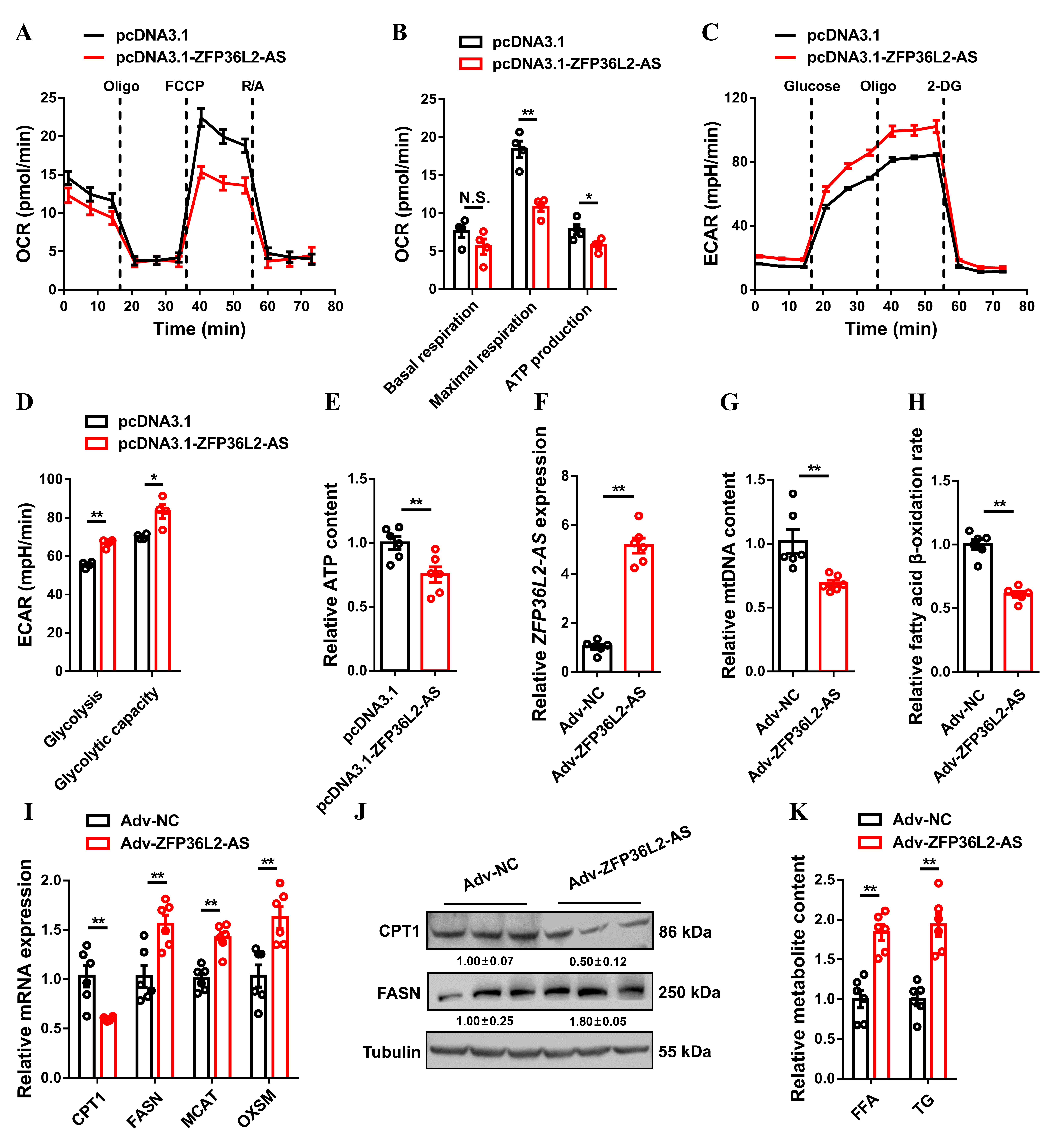

Supplement: Supplementary file 8 — Supplementary Figure 3 [file 41419_2022_4772_MOESM8_ESM.tif]

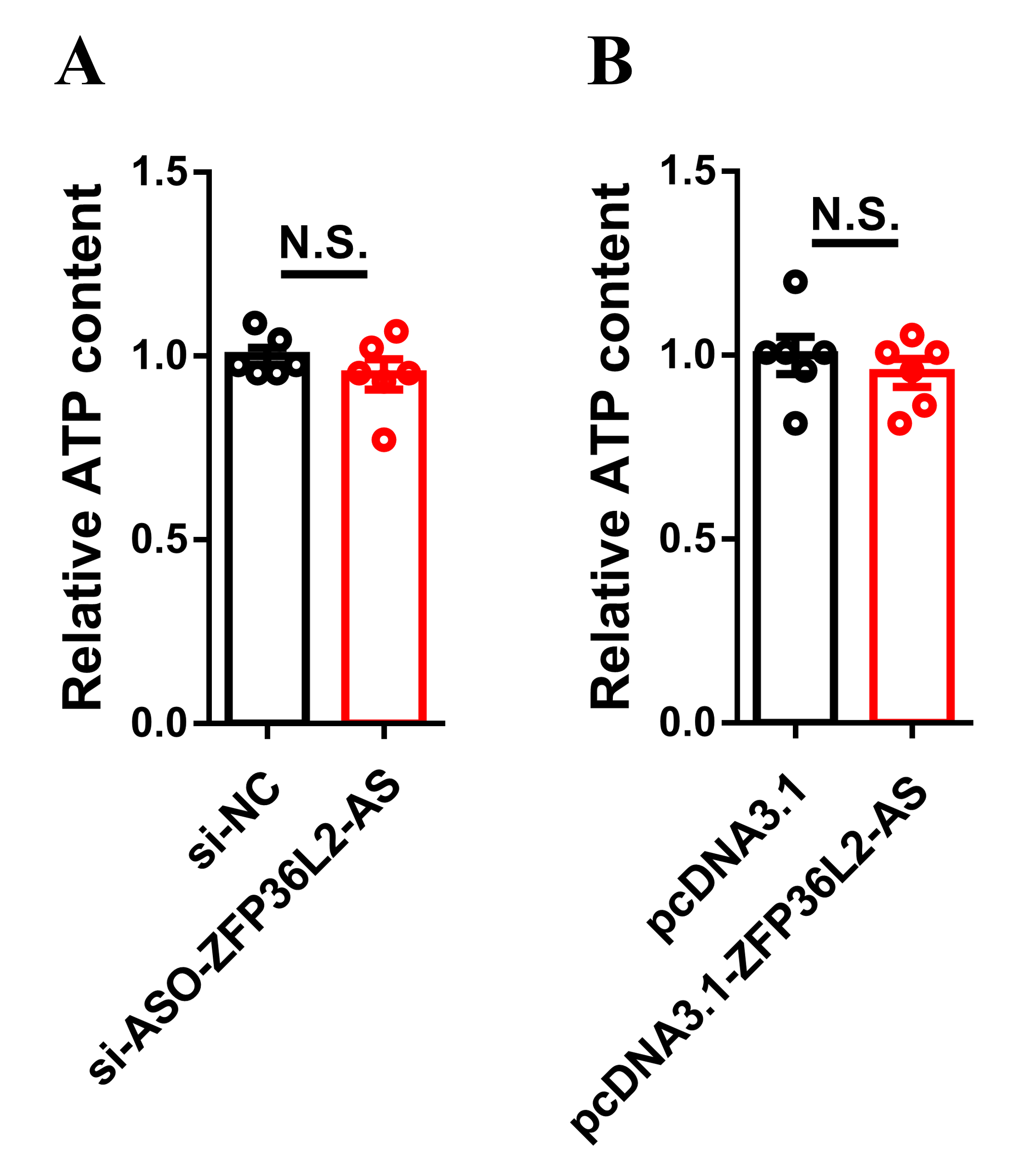

Supplement: Supplementary file 9 — Supplementary Figure 4 [file 41419_2022_4772_MOESM9_ESM.tif]

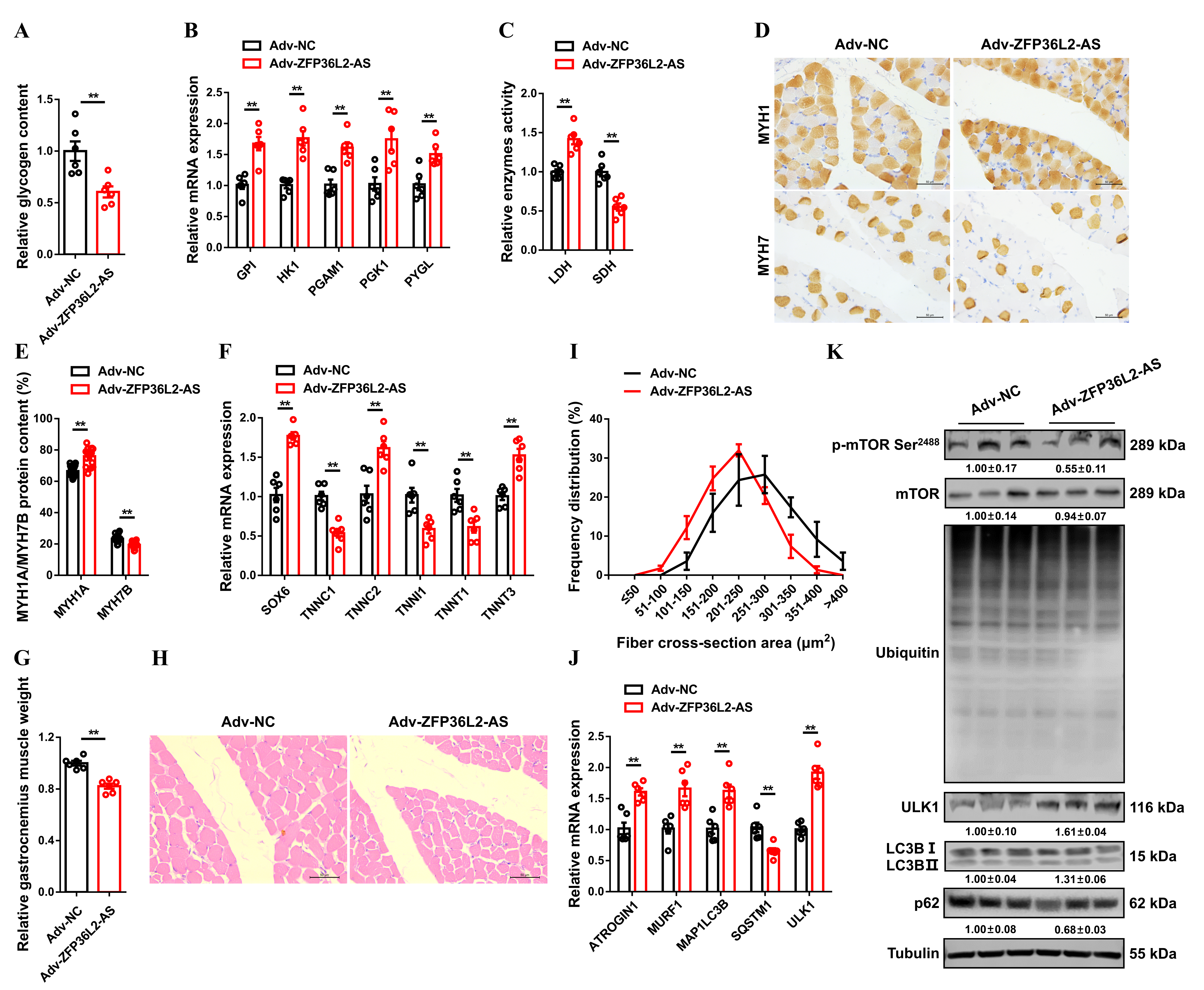

Supplement: Supplementary file 10 — Supplementary Figure 5 [file 41419_2022_4772_MOESM10_ESM.tif]

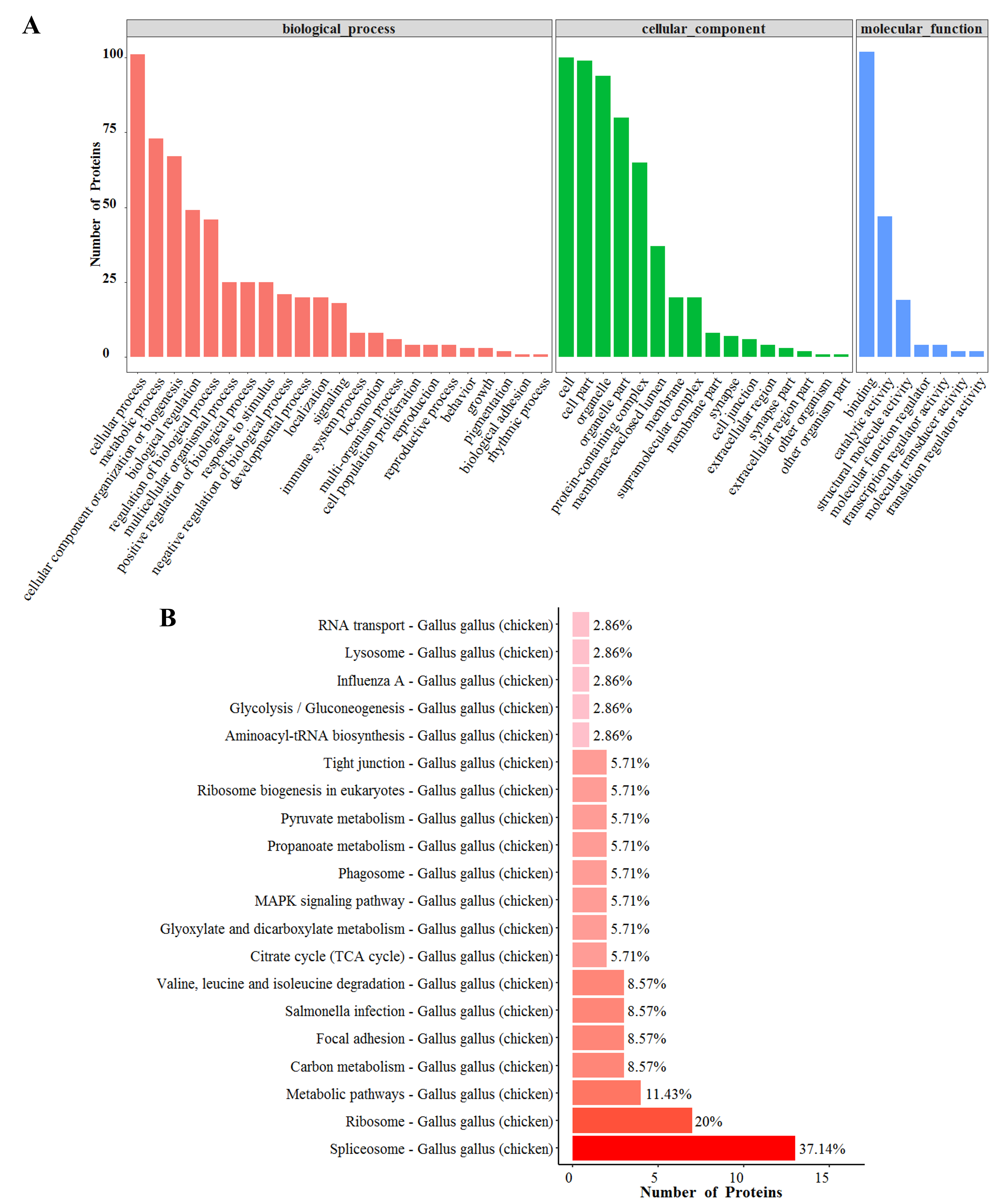

Supplement: Supplementary file 11 — Supplementary Figure 6 [file 41419_2022_4772_MOESM11_ESM.tif]

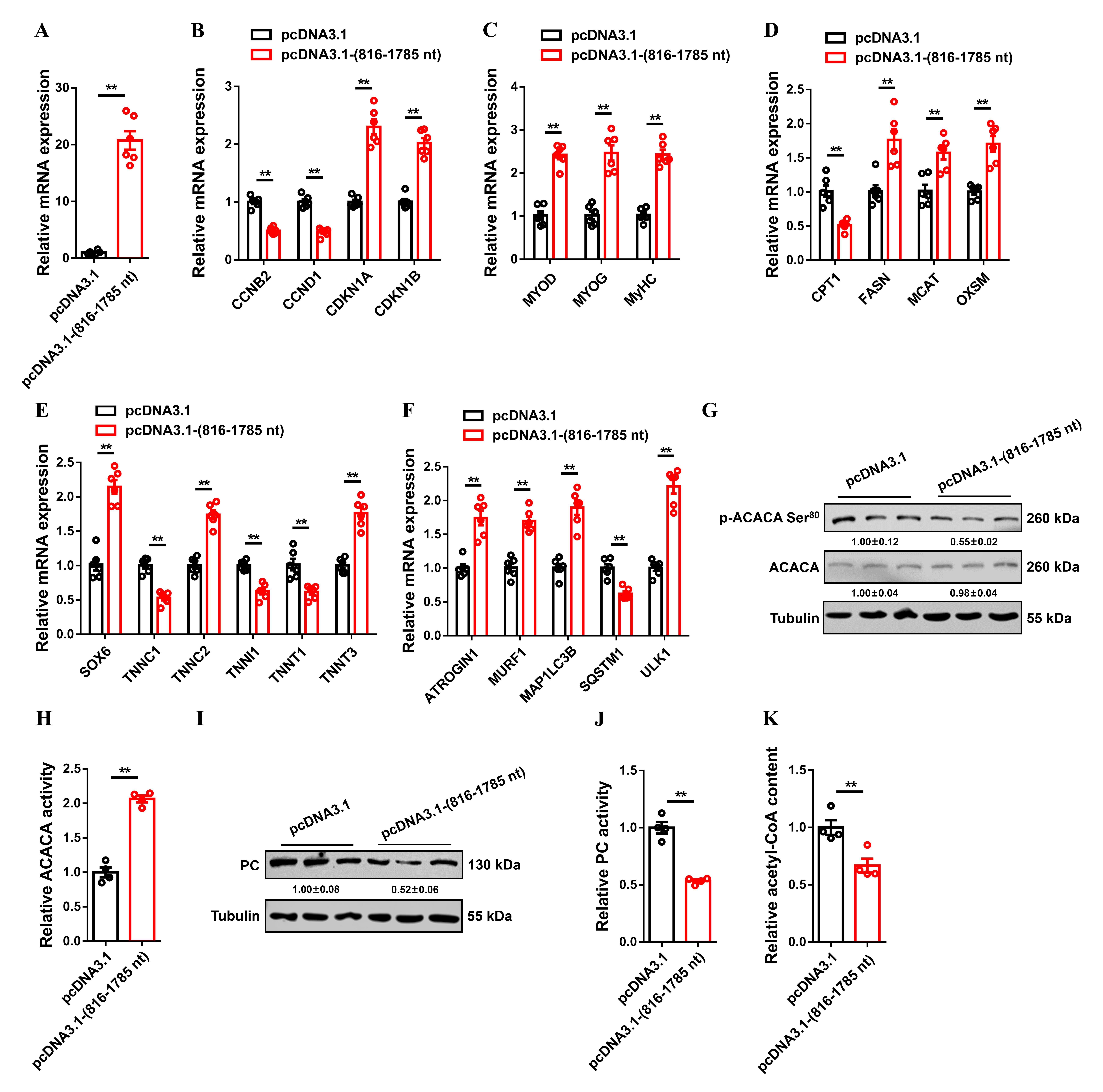

Supplement: Supplementary file 12 — Supplementary Figure 7 [file 41419_2022_4772_MOESM12_ESM.tif]

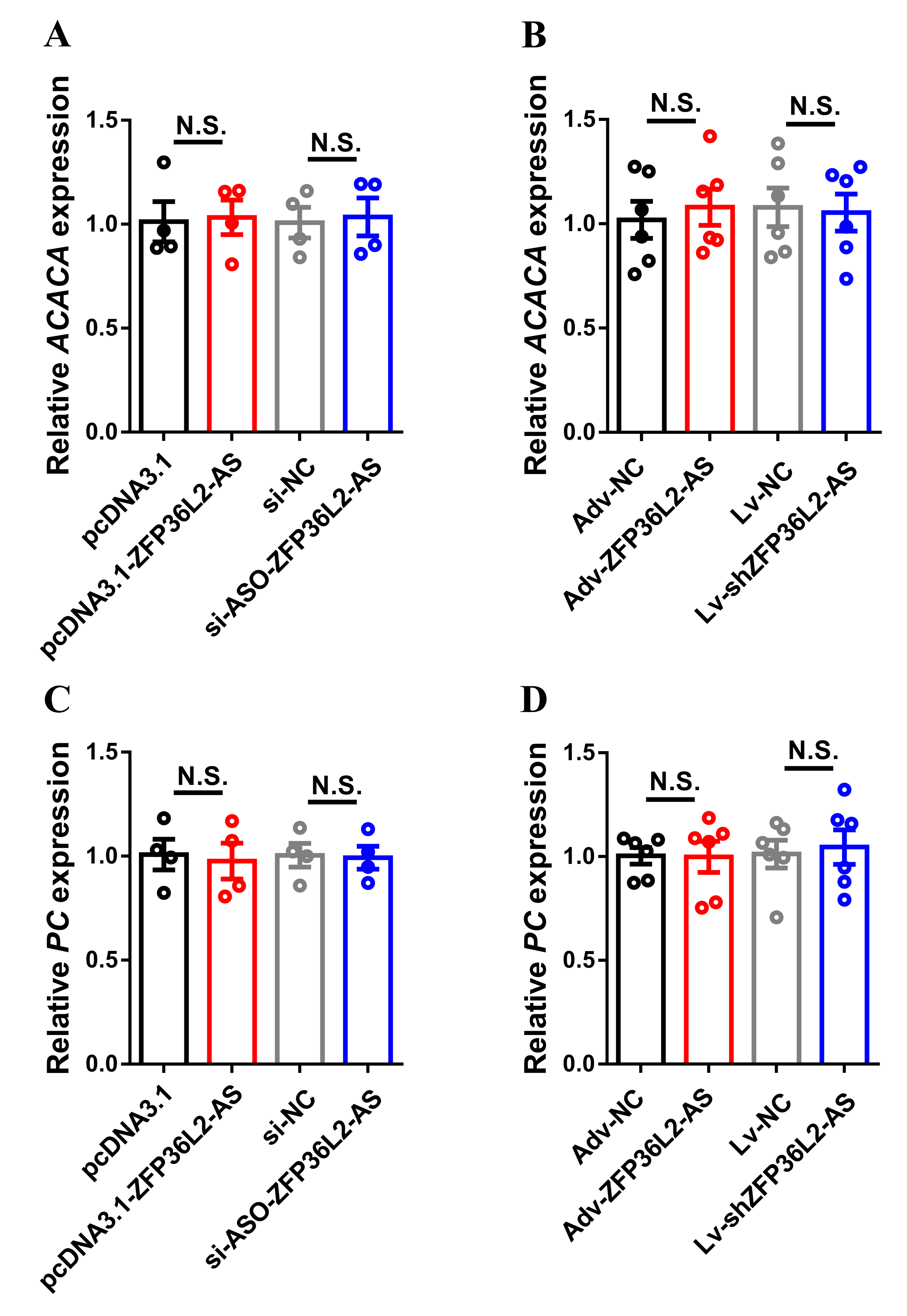

Supplement: Supplementary file 13 — Supplementary Figure 8 [file 41419_2022_4772_MOESM13_ESM.tif]

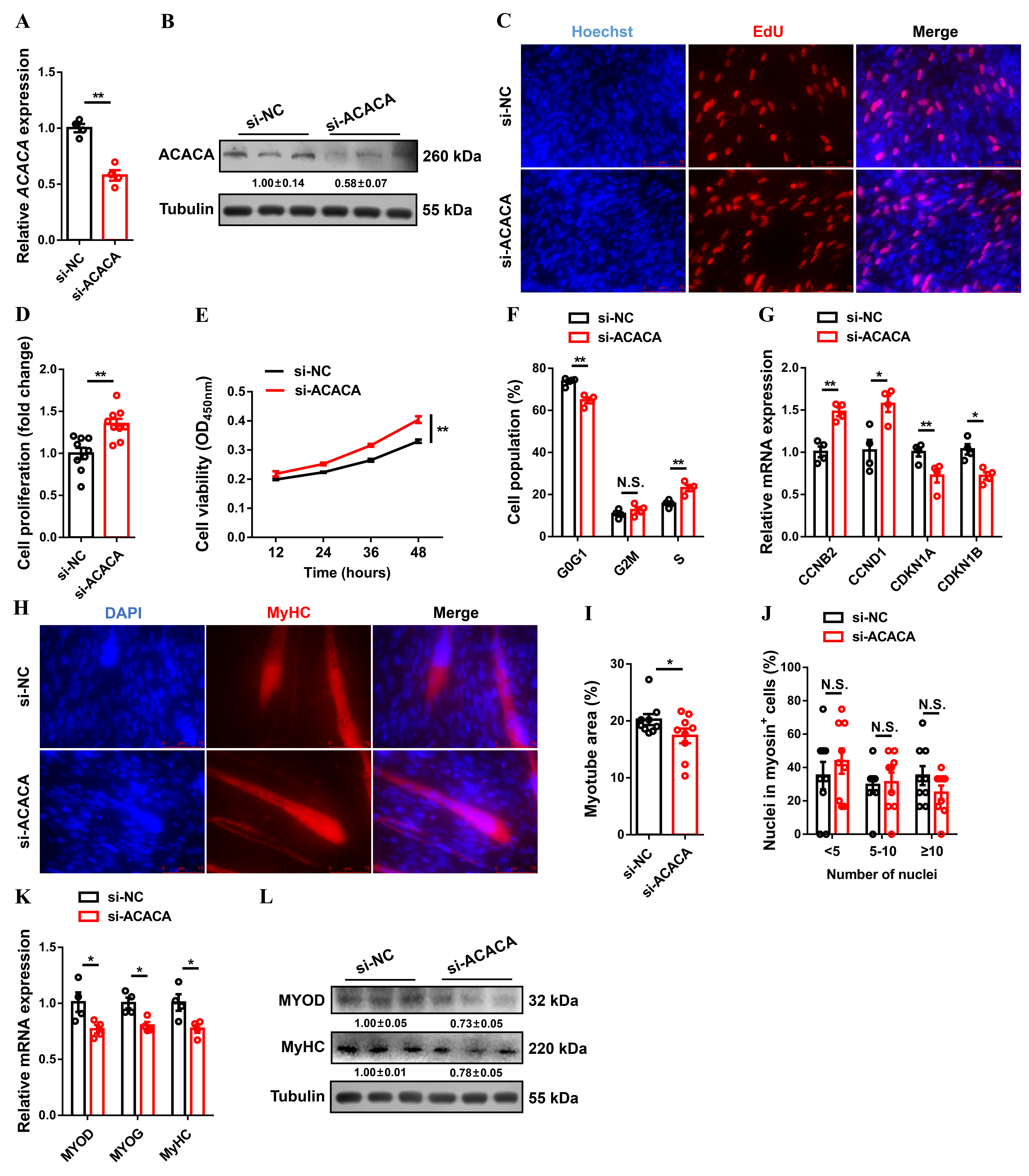

Supplement: Supplementary file 14 — Supplementary Figure 9 [file 41419_2022_4772_MOESM14_ESM.tif]

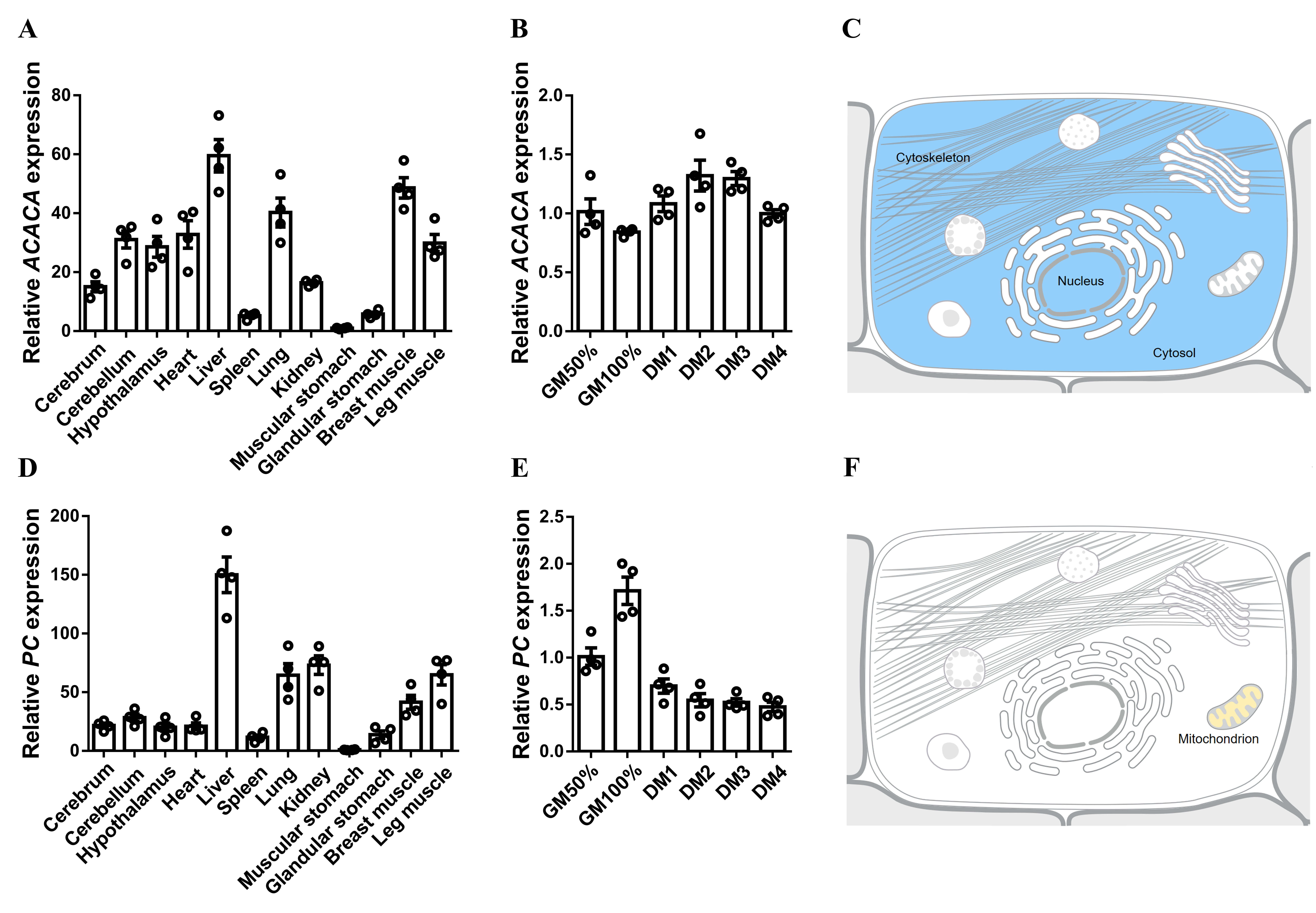

Supplement: Supplementary file 15 — Supplementary Figure 10 [file 41419_2022_4772_MOESM15_ESM.tif]

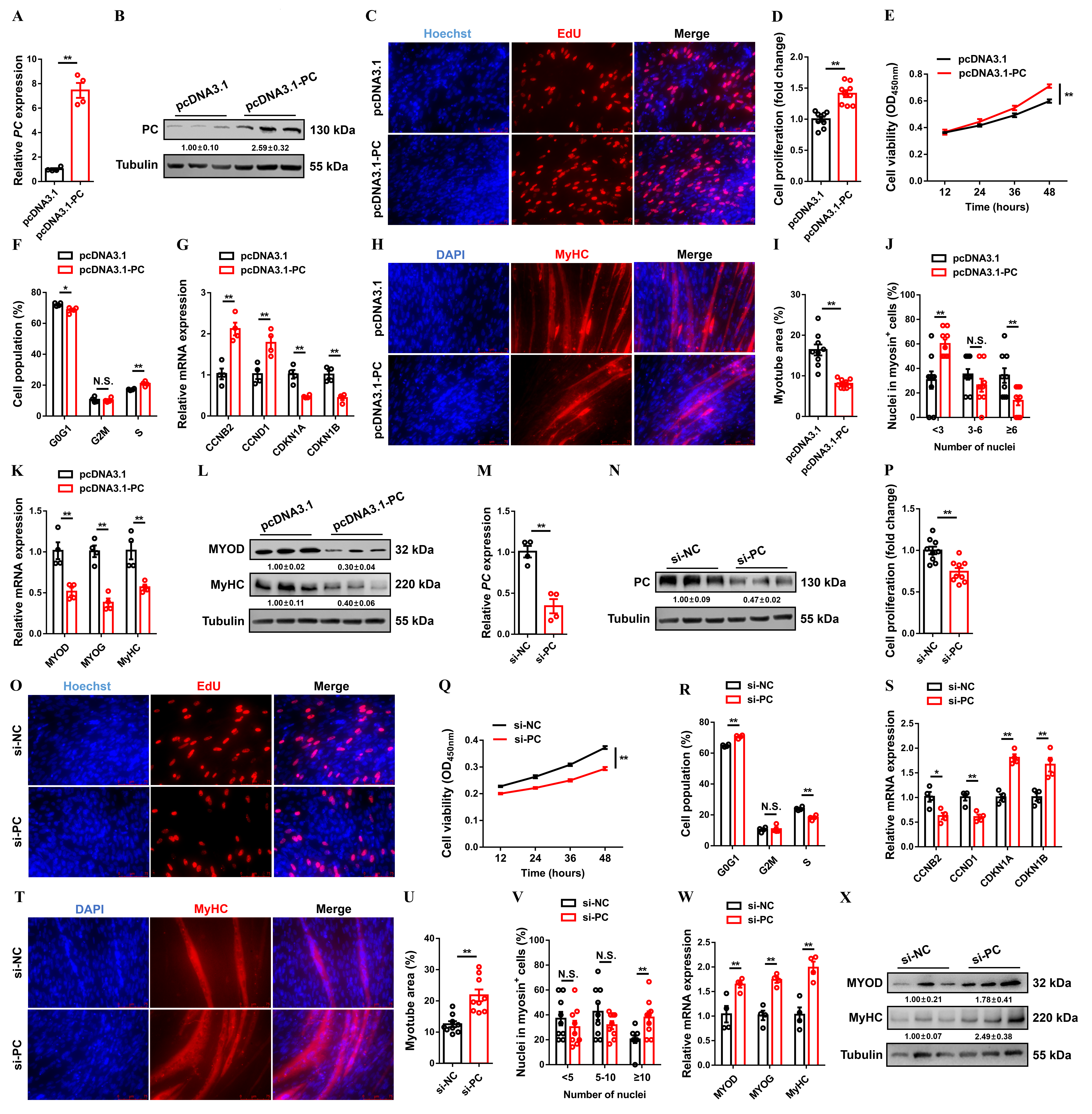

Supplement: Supplementary file 16 — Supplementary Figure 11 [file 41419_2022_4772_MOESM16_ESM.tif]

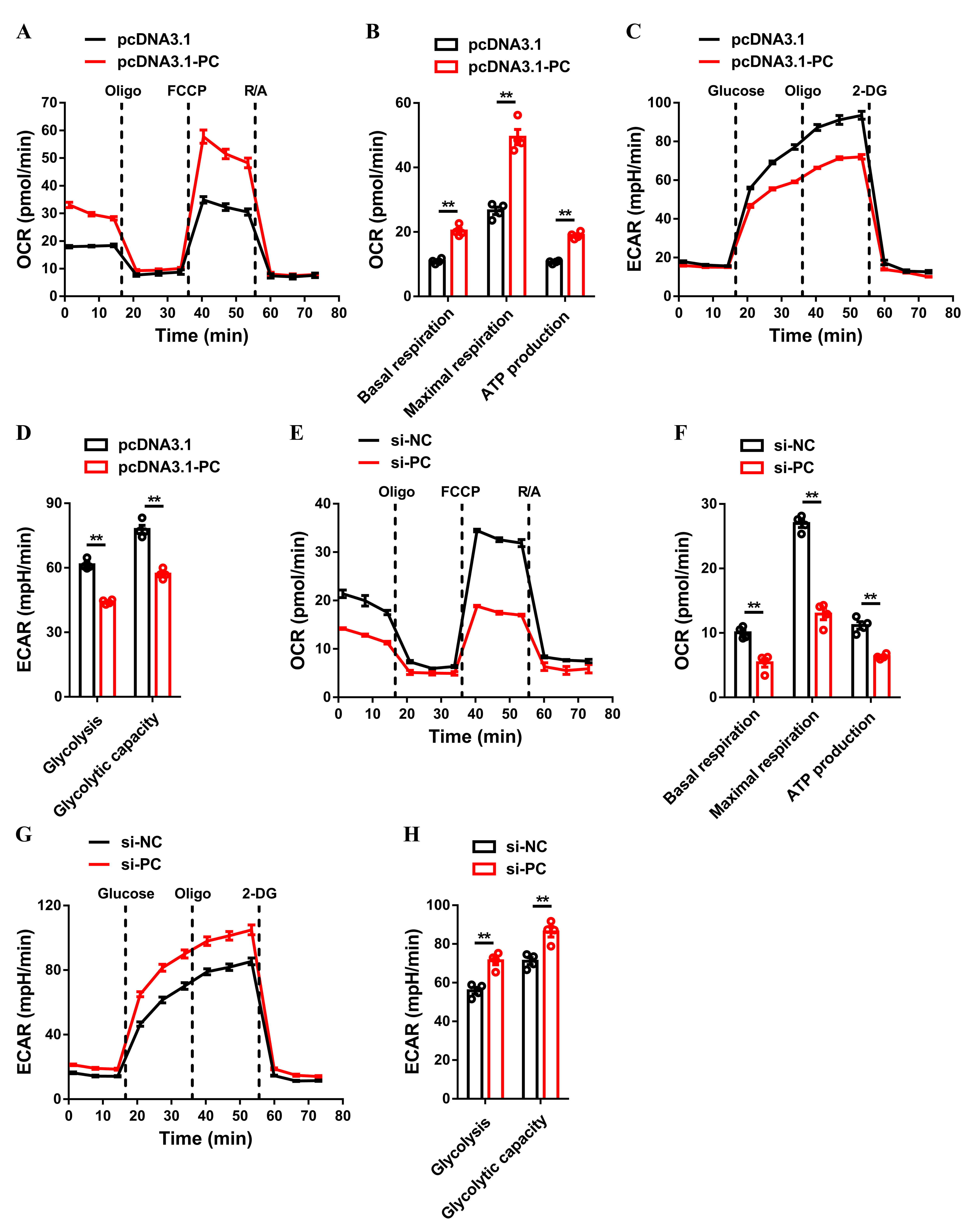

Supplement: Supplementary file 17 — Supplementary Figure 12 [file 41419_2022_4772_MOESM17_ESM.tif]

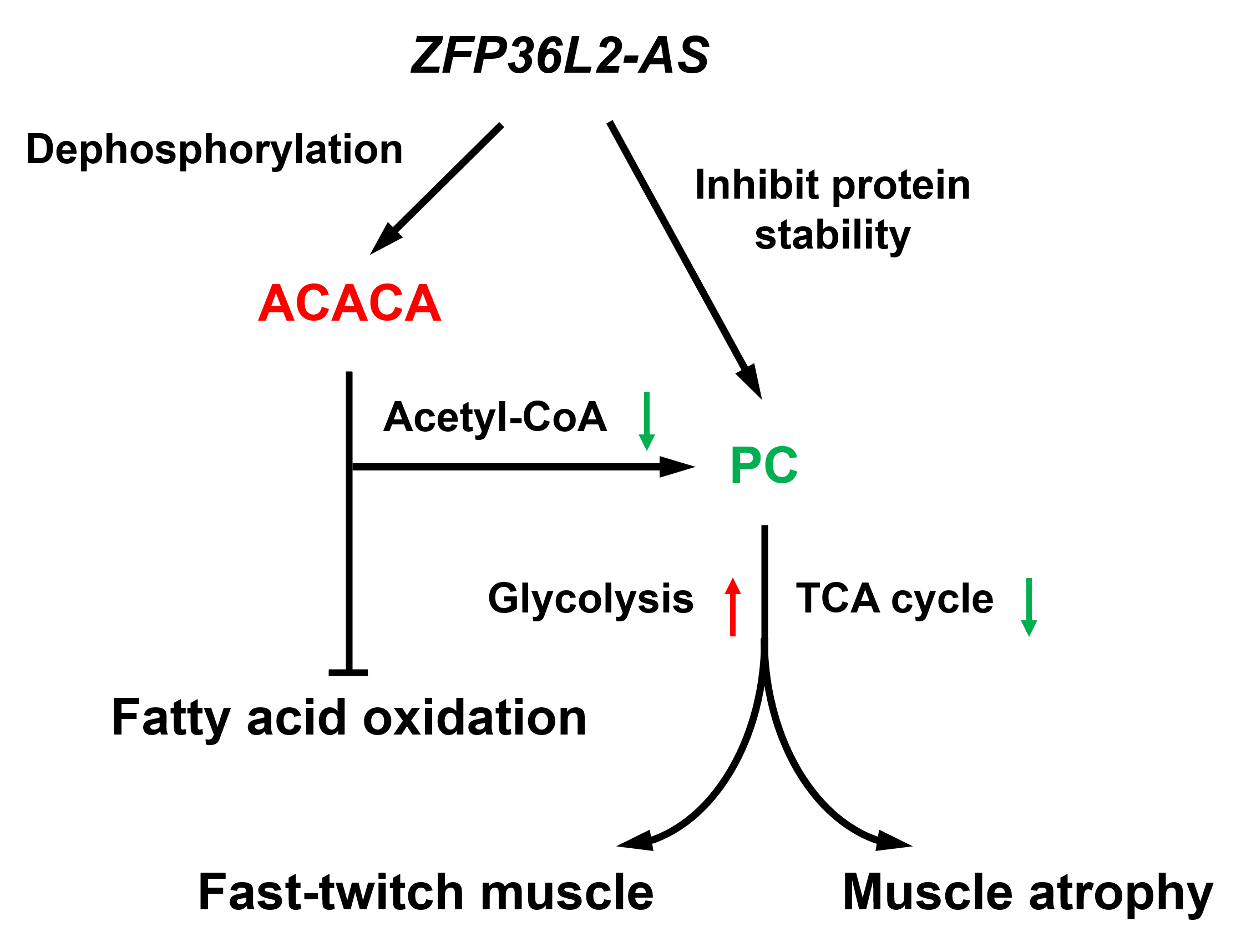

Supplement: Supplementary file 18 — Supplementary Figure 13 [file 41419_2022_4772_MOESM18_ESM.tif]
