# Supplementary material for: Long noncoding RNA ZFP36L2-AS functions as a metabolic modulator to regulate muscle development
Source: Cell Death Dis. 2022 Apr 21;13(4):389. doi: 10.1038/s41419-022-04772-2 (PMC9023450; doi:10.1038/s41419-022-04772-2)

Fig. 1H anti-Flag

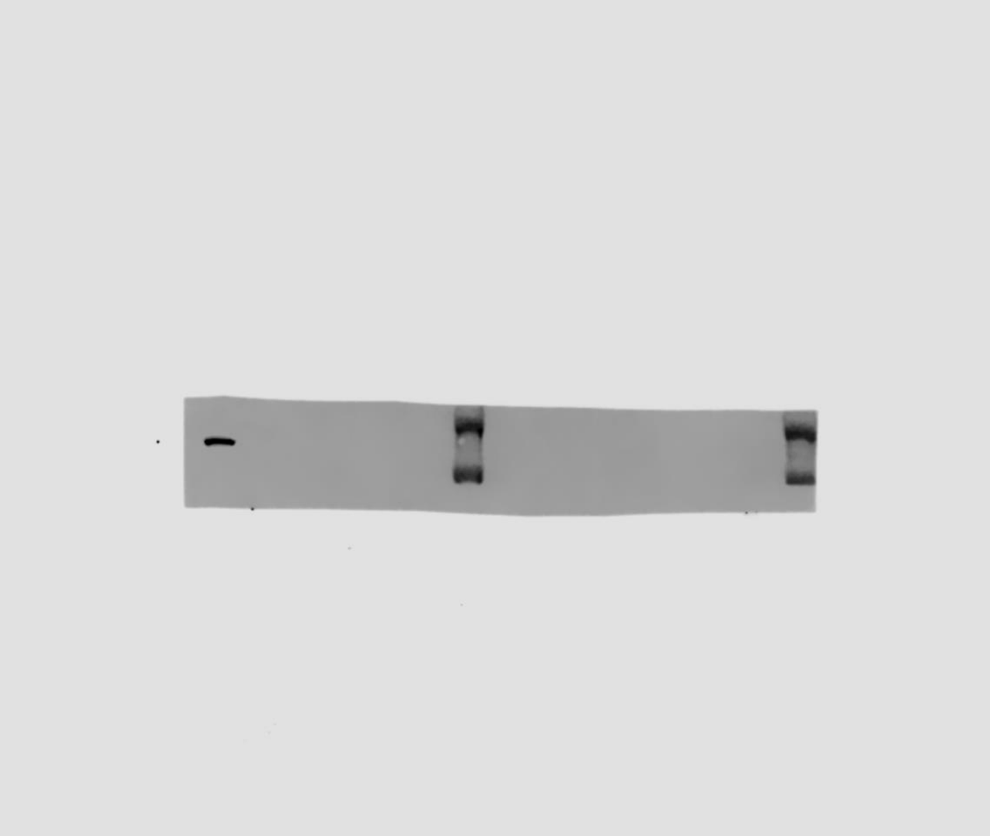

Fig. 1H anti-Tubulin

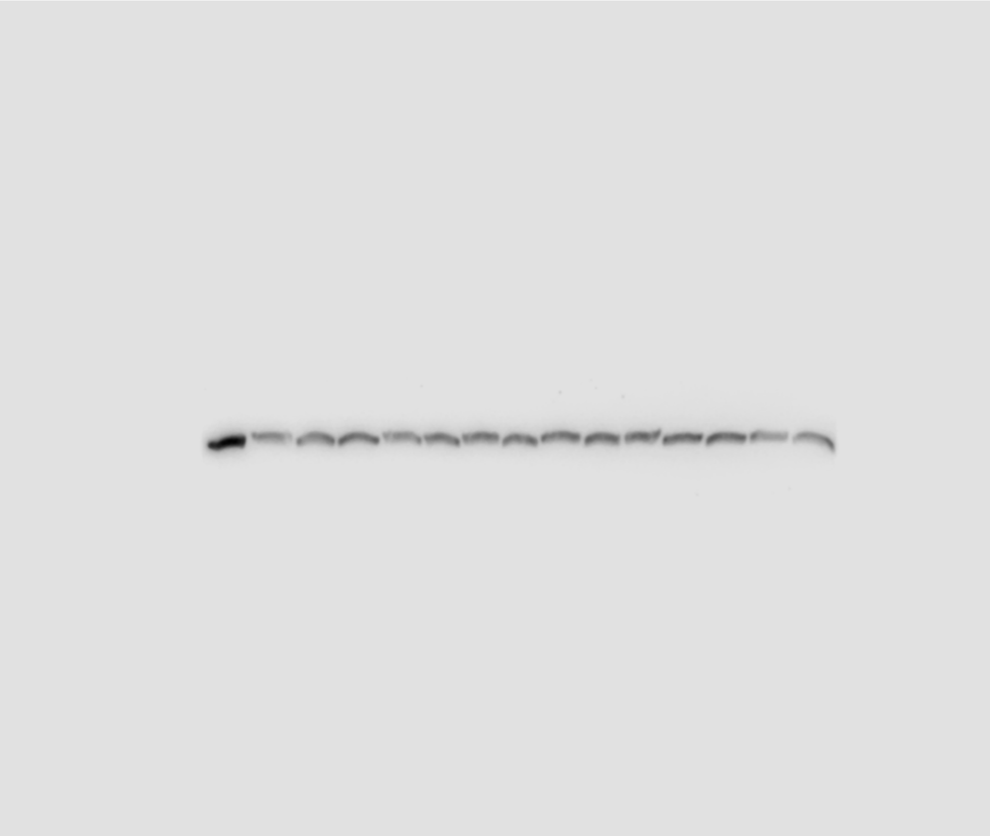

Fig. 1H ORF anti-Flag

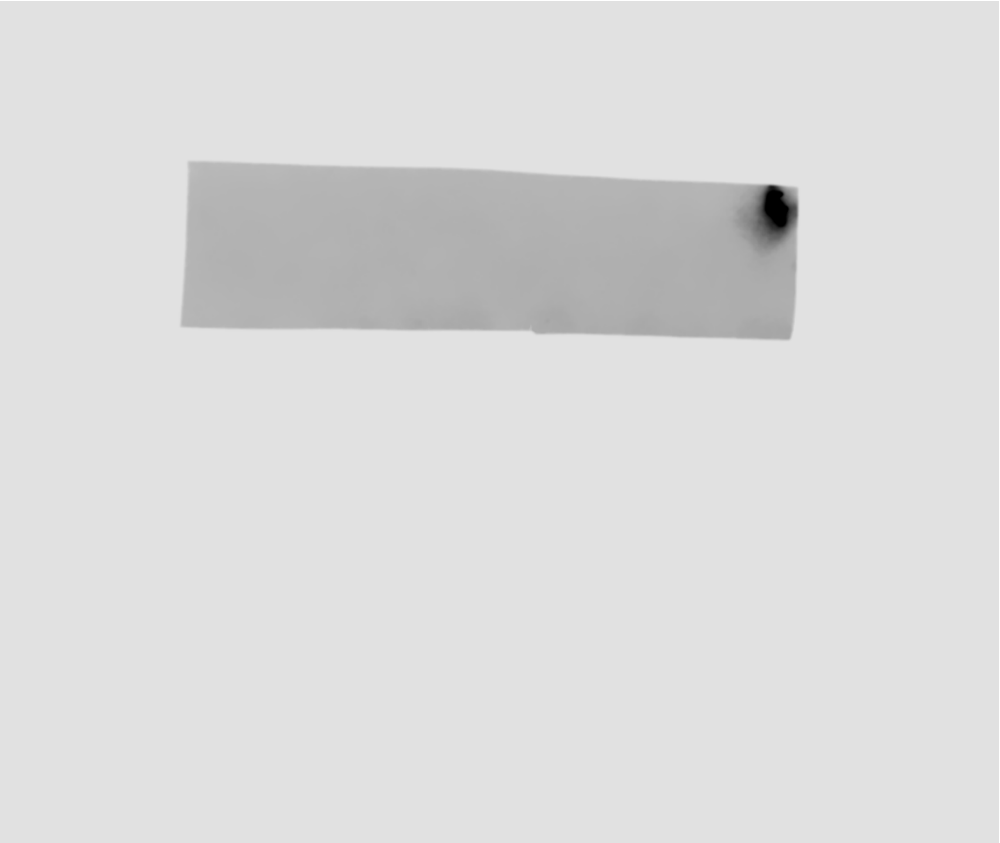

Fig. 2K anti-MyHC

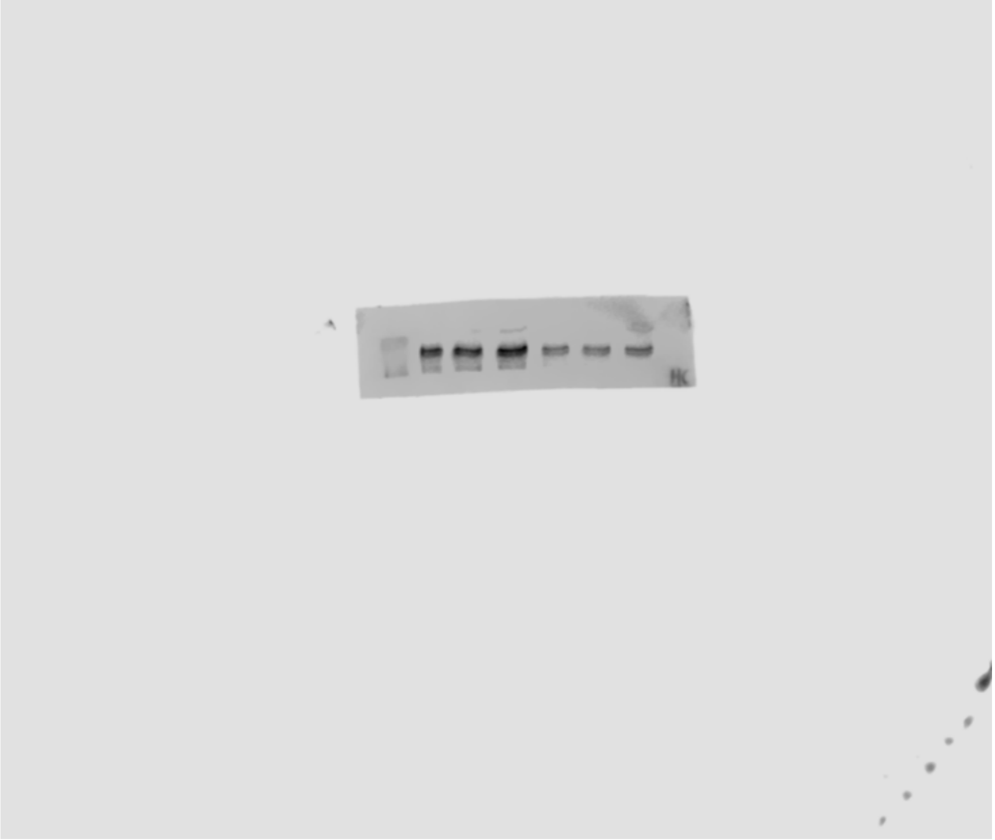

Fig. 2K anti-MYOD

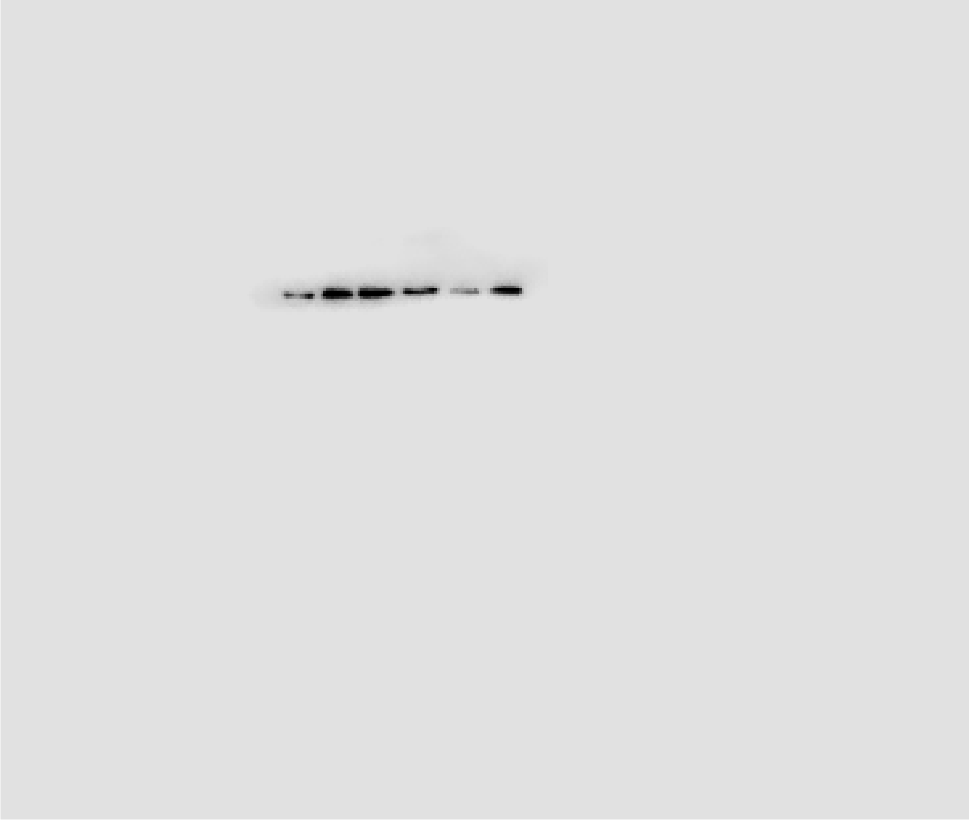

Fig. 2K anti-Tubulin (Right)

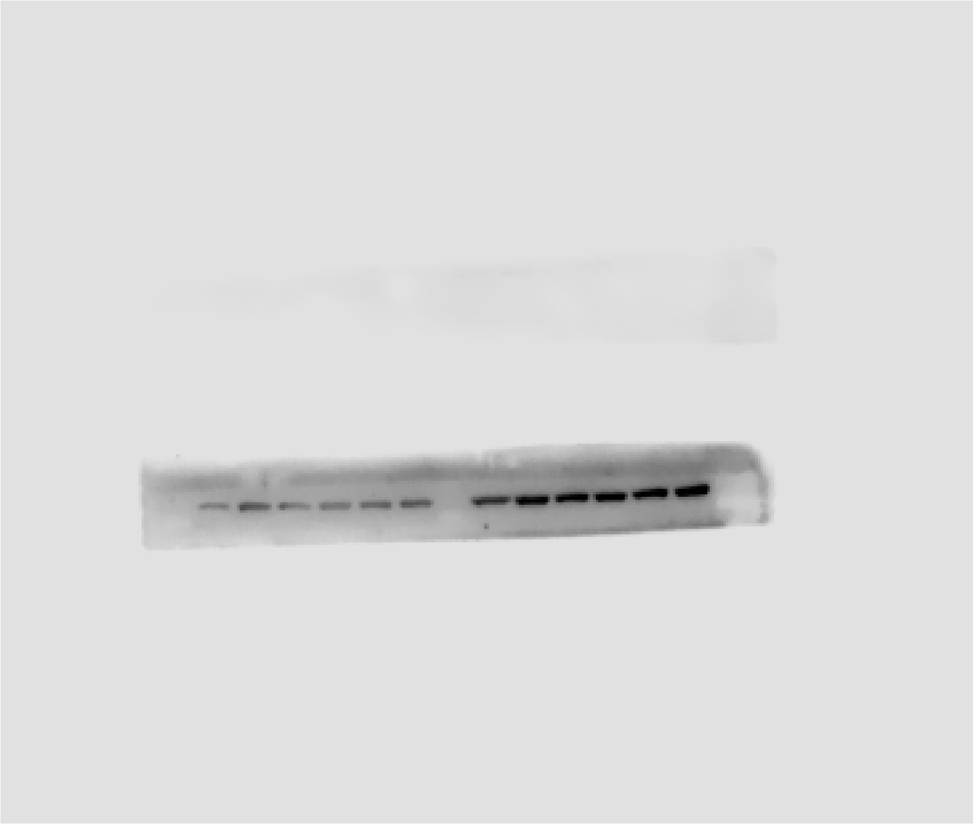

Fig. 3J anti-CPT1 (Left)

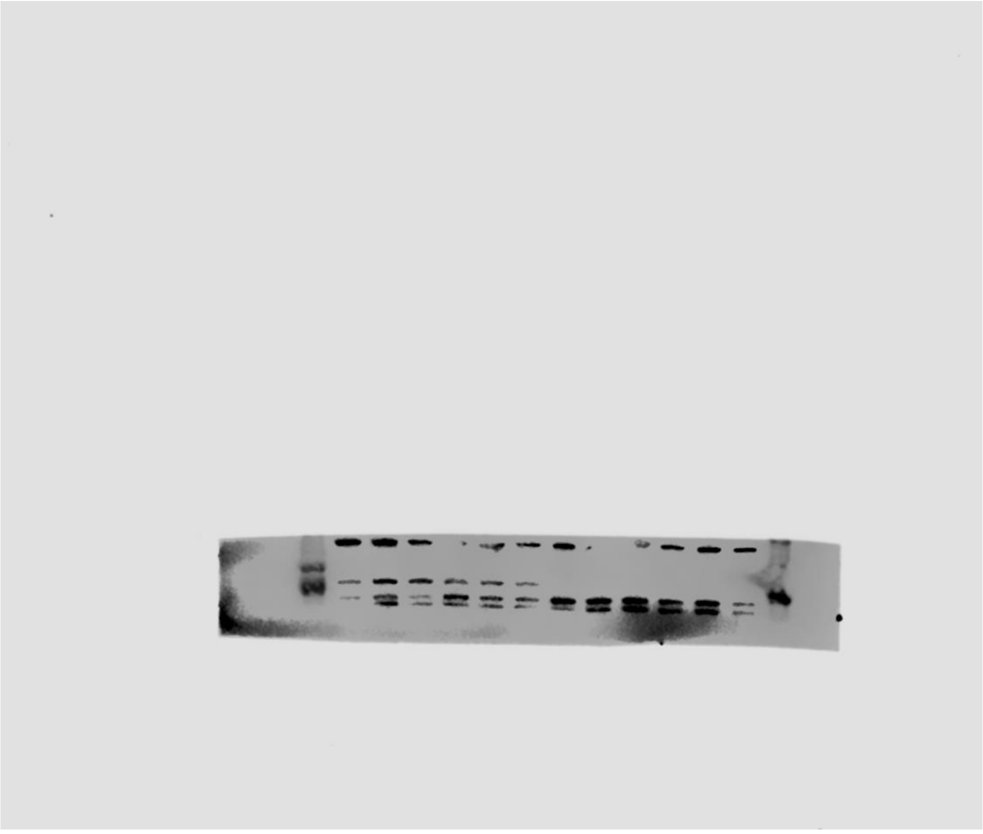

Fig. 3J anti-FASN (Right)

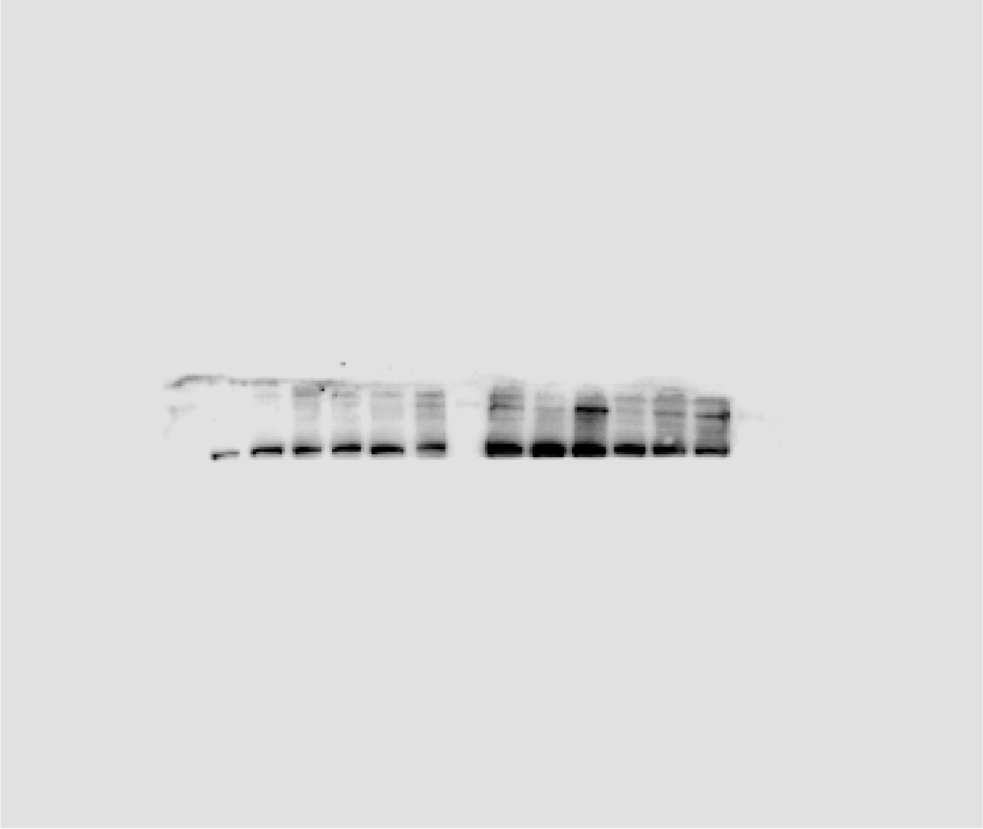

Fig. 3J anti-Tubulin (Left)

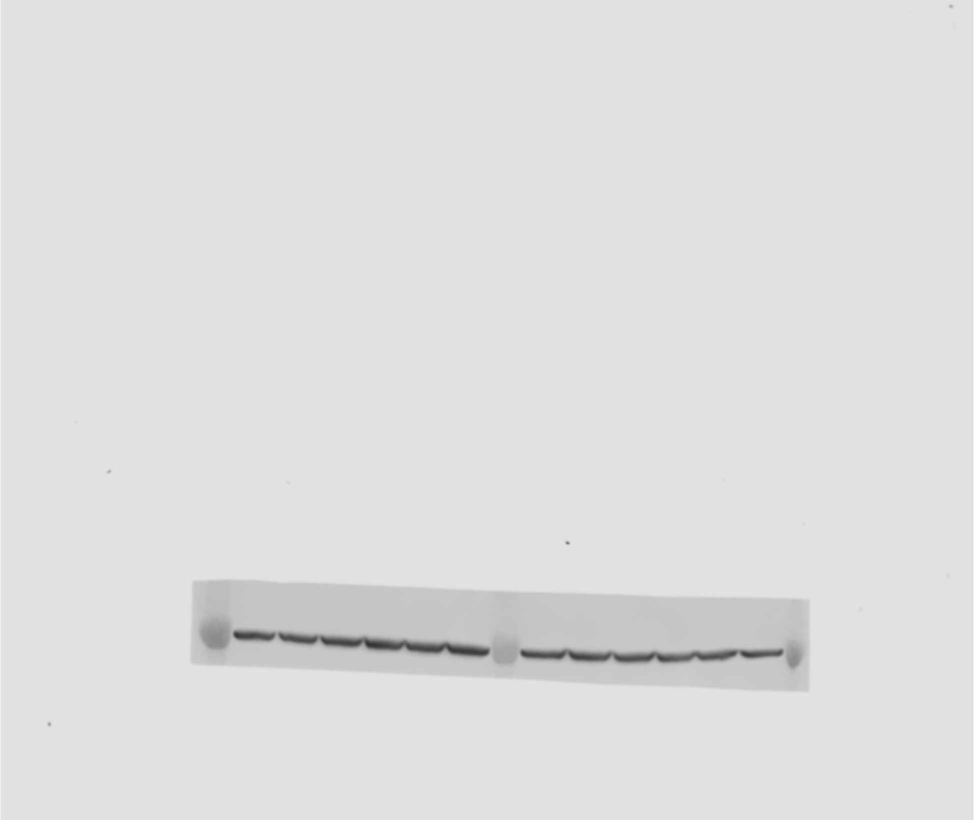

Fig. 5K anti-LC3B

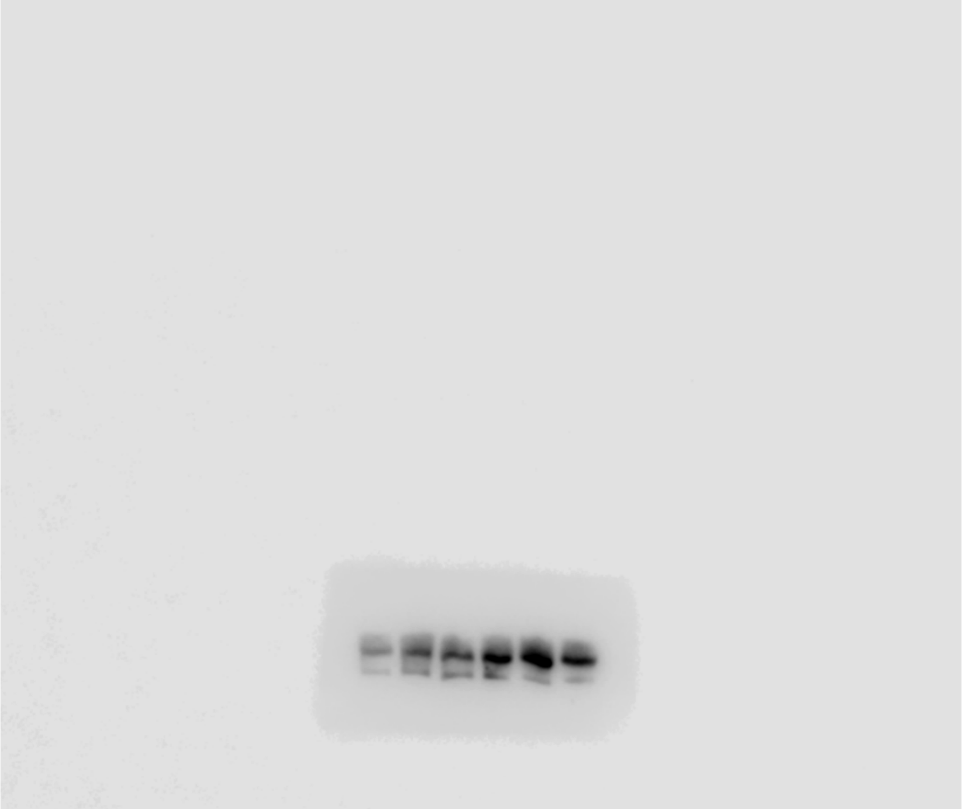

Fig. 5K anti-mTOR

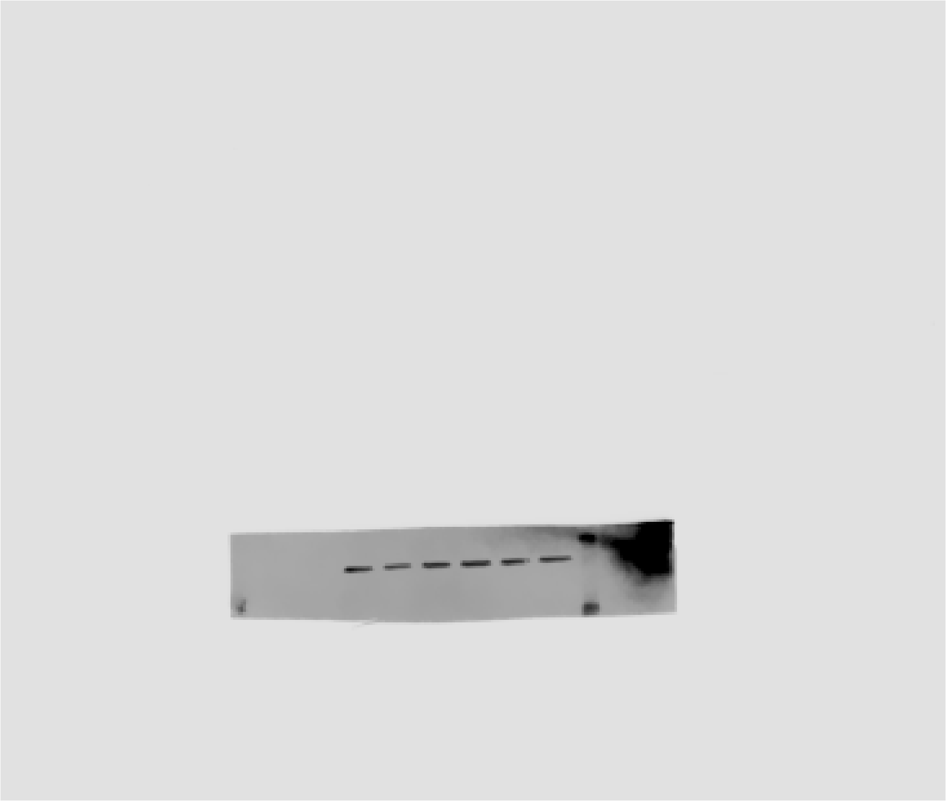

Fig. 5K anti-p62 (Right)

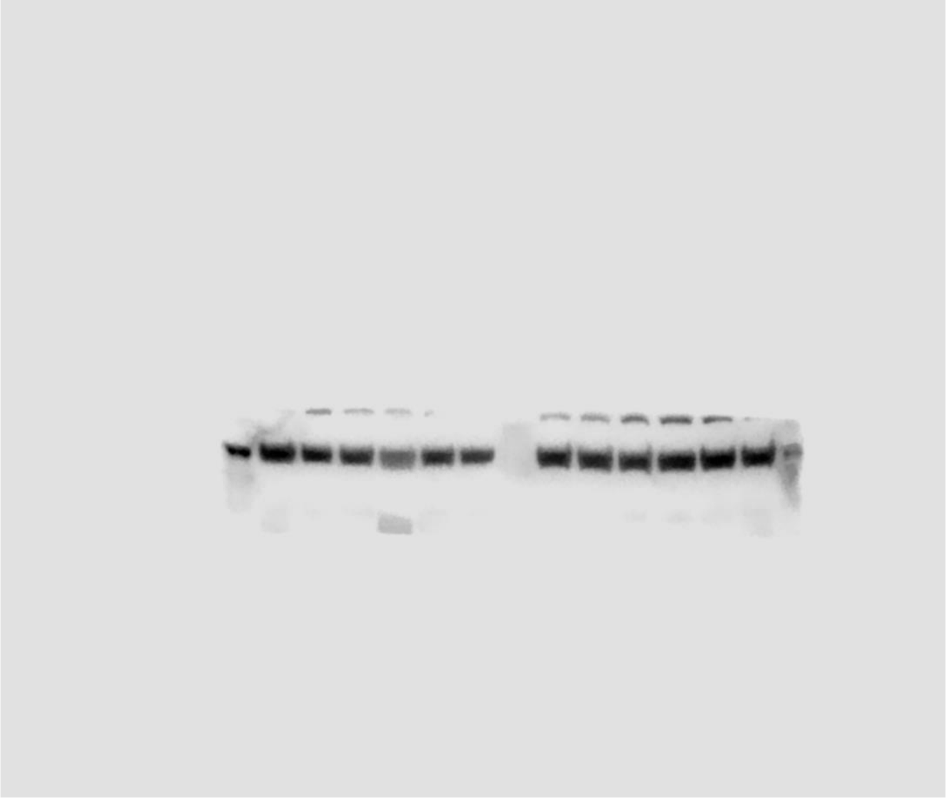

Fig. 5K anti-p-mTOR (Right)

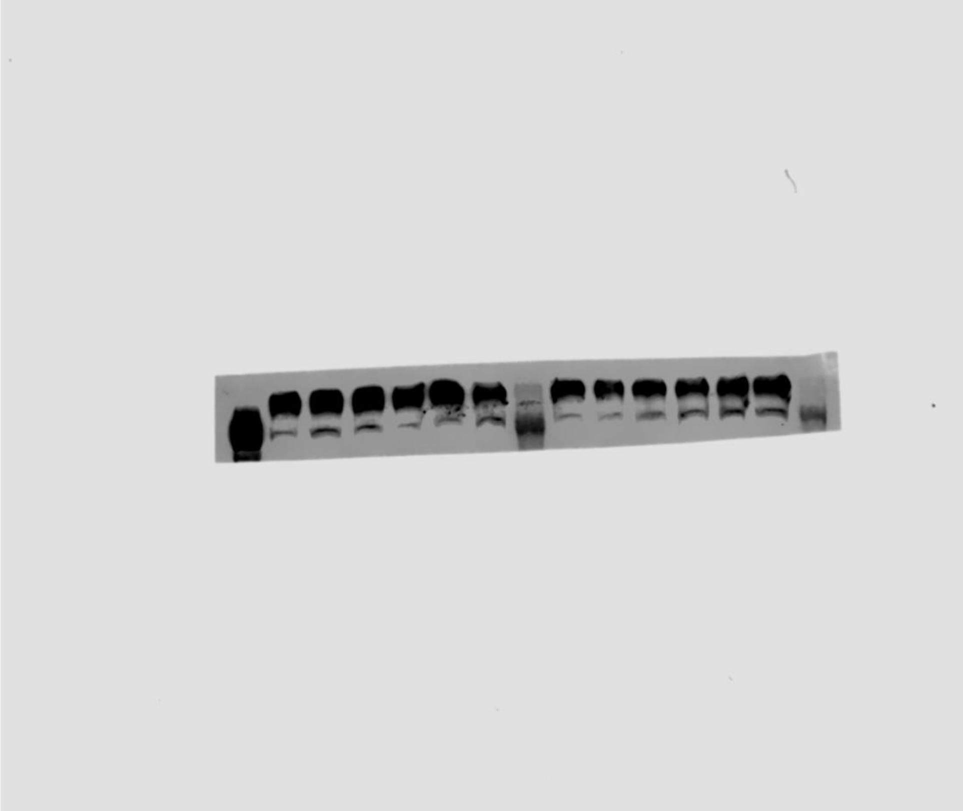

Fig. 5K anti-Tubulin (Left)

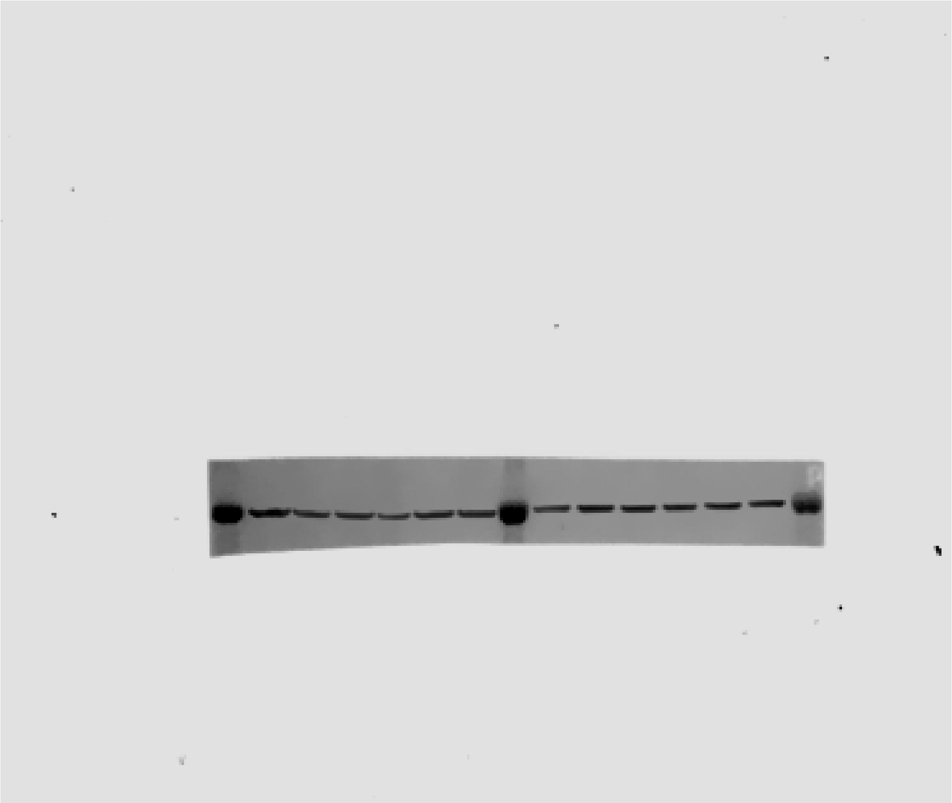

Fig. 5K anti-Ubiquitin (Right)

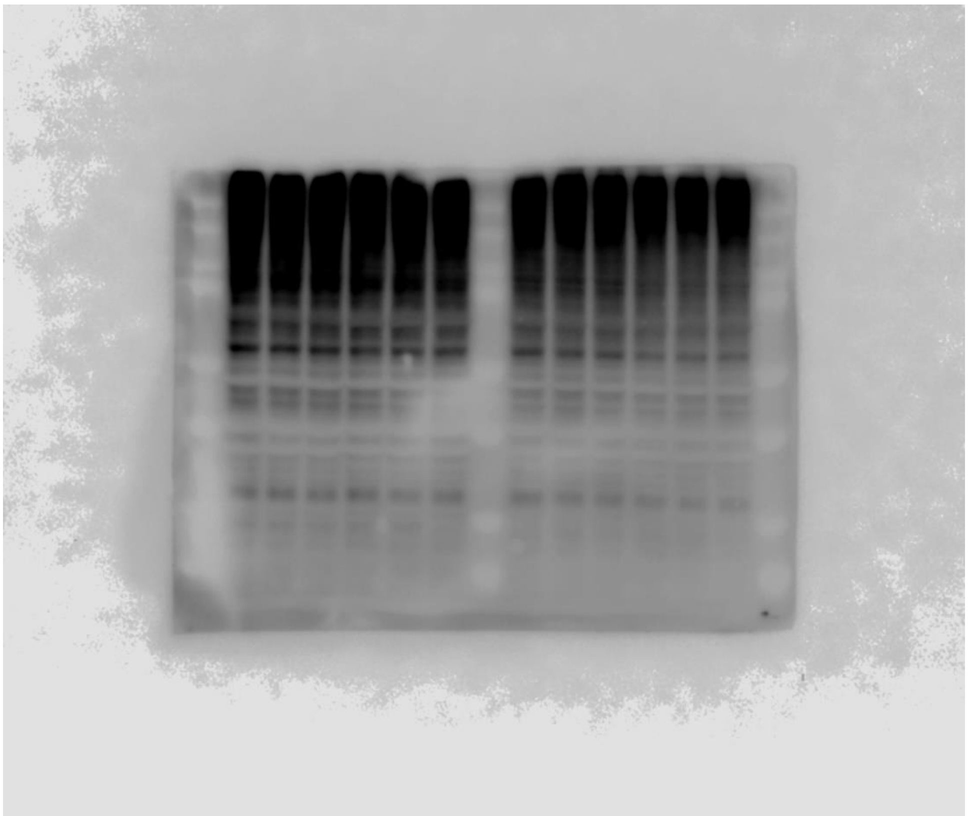

Fig. 5K anti-ULK1 (Left)

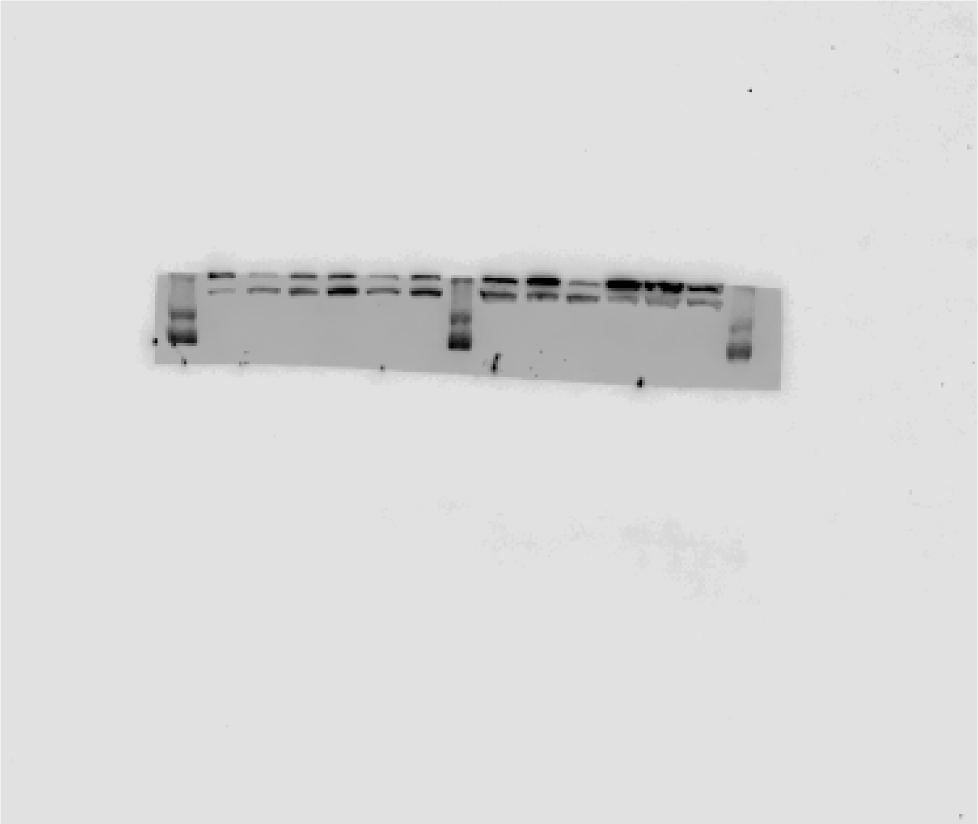

Fig. 6A anti-ACACA

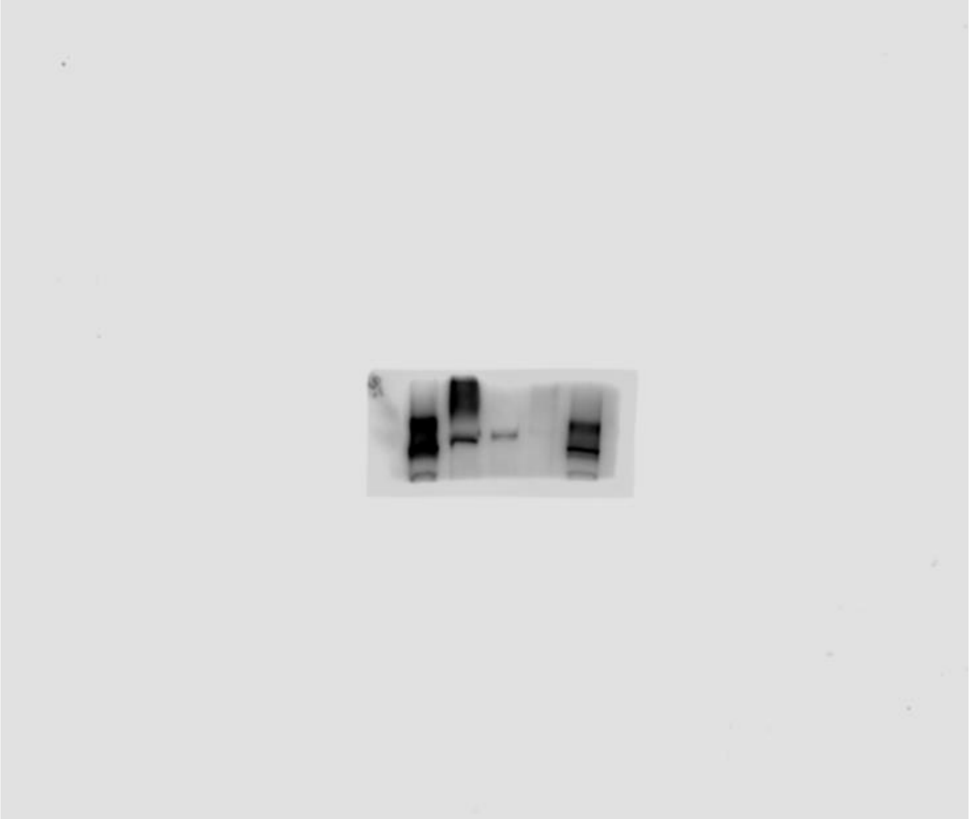

Fig. 6A anti-PC

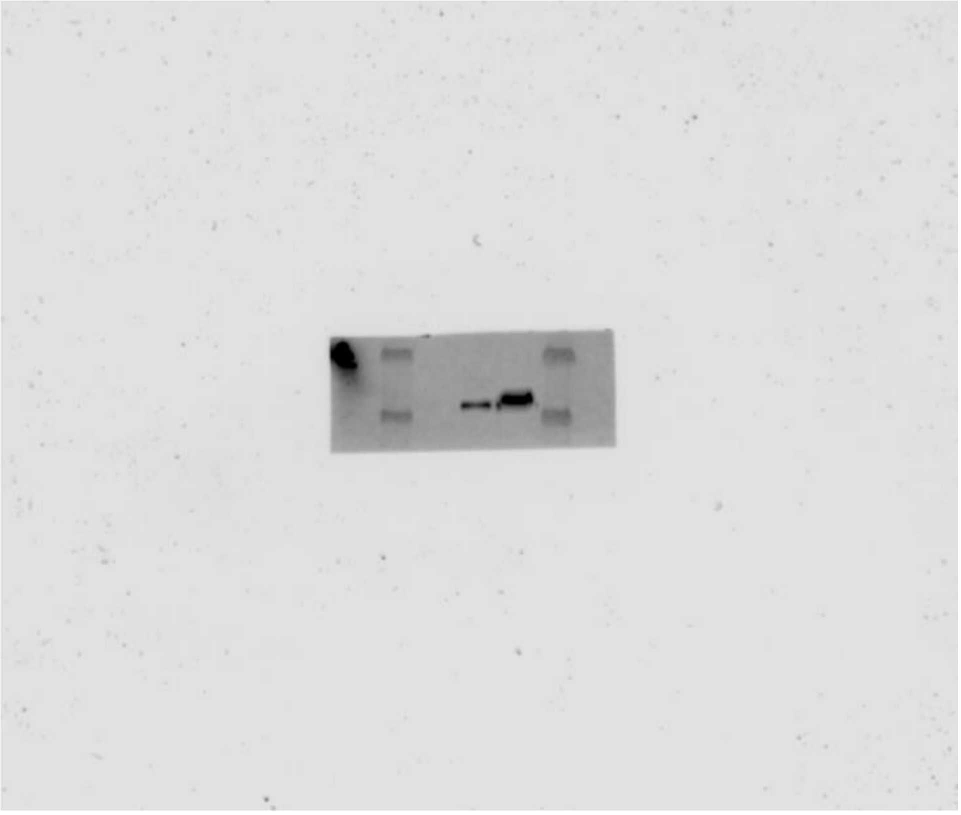

Fig. 6A anti-Tublin

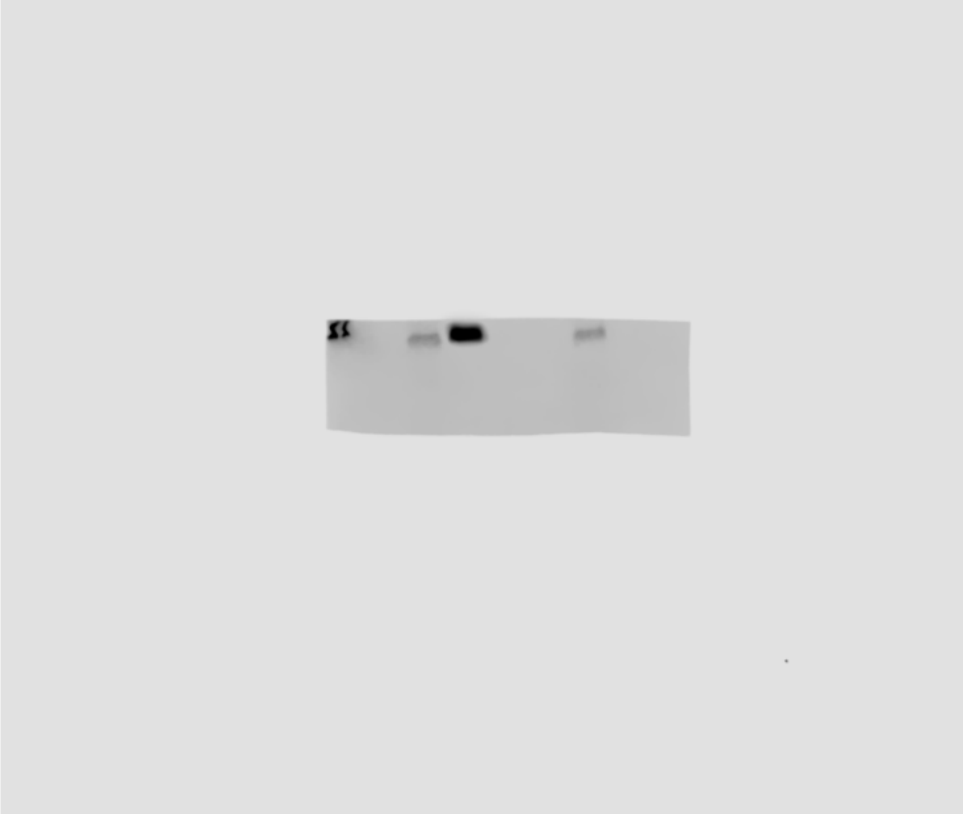

Fig. 6C anti-ACACA

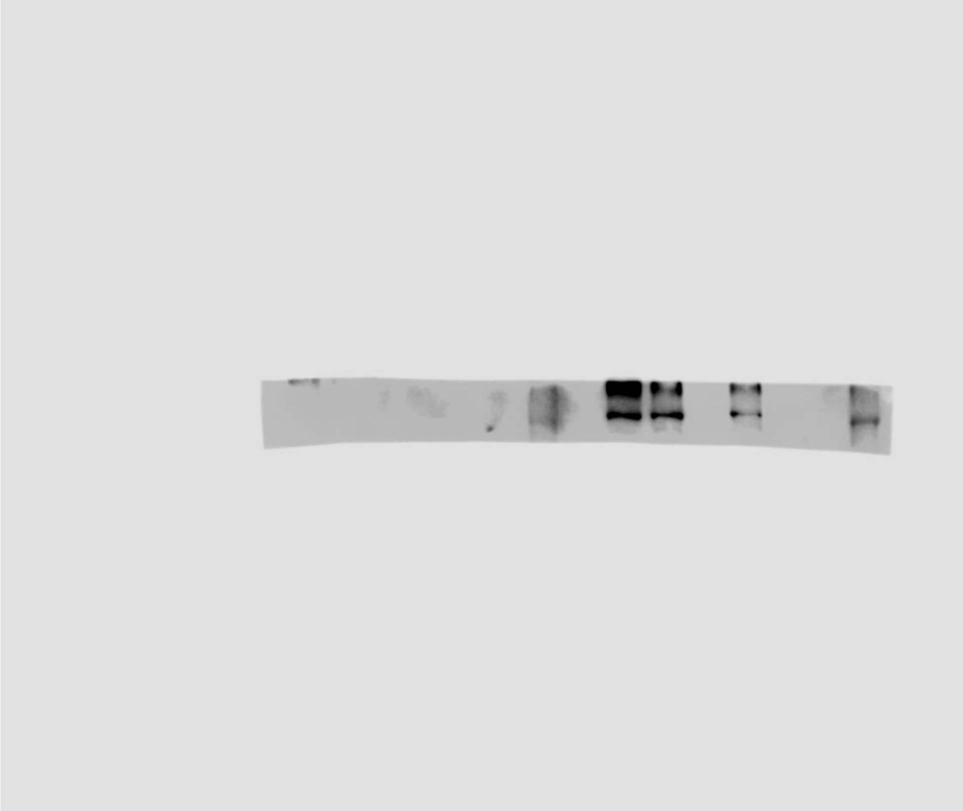

Fig. 6C anti-PC

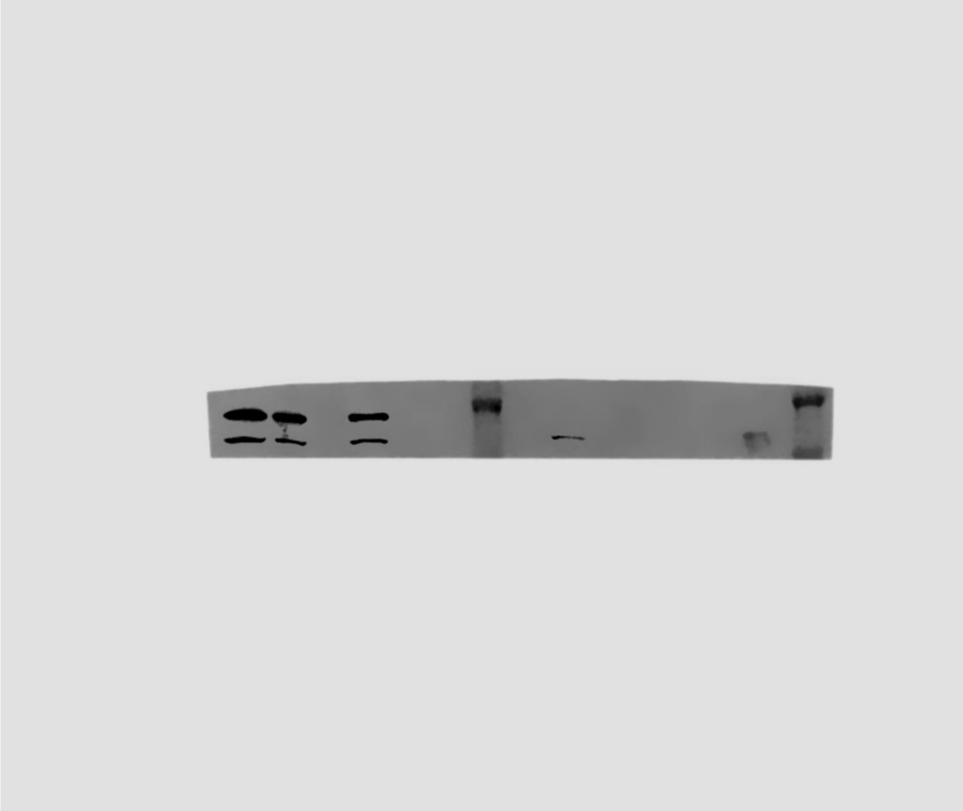

Fig. 6C anti-Tubulin

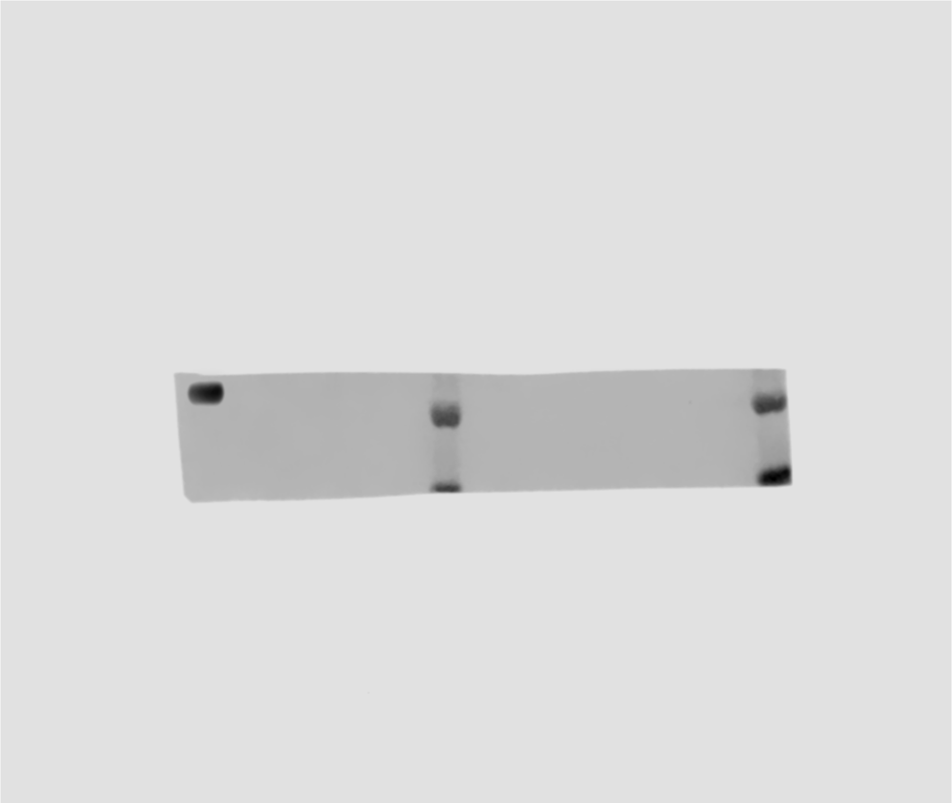

Fig. 6D (Left) anti-ACACA

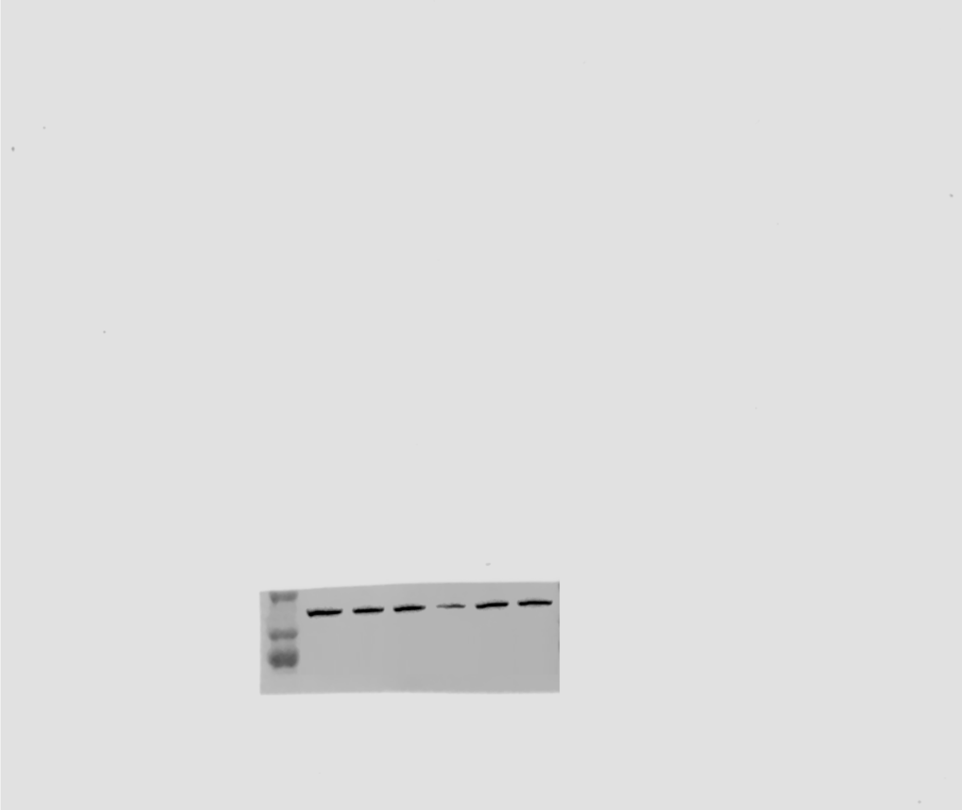

Fig. 6D (Left) anti-p-ACACA Ser80

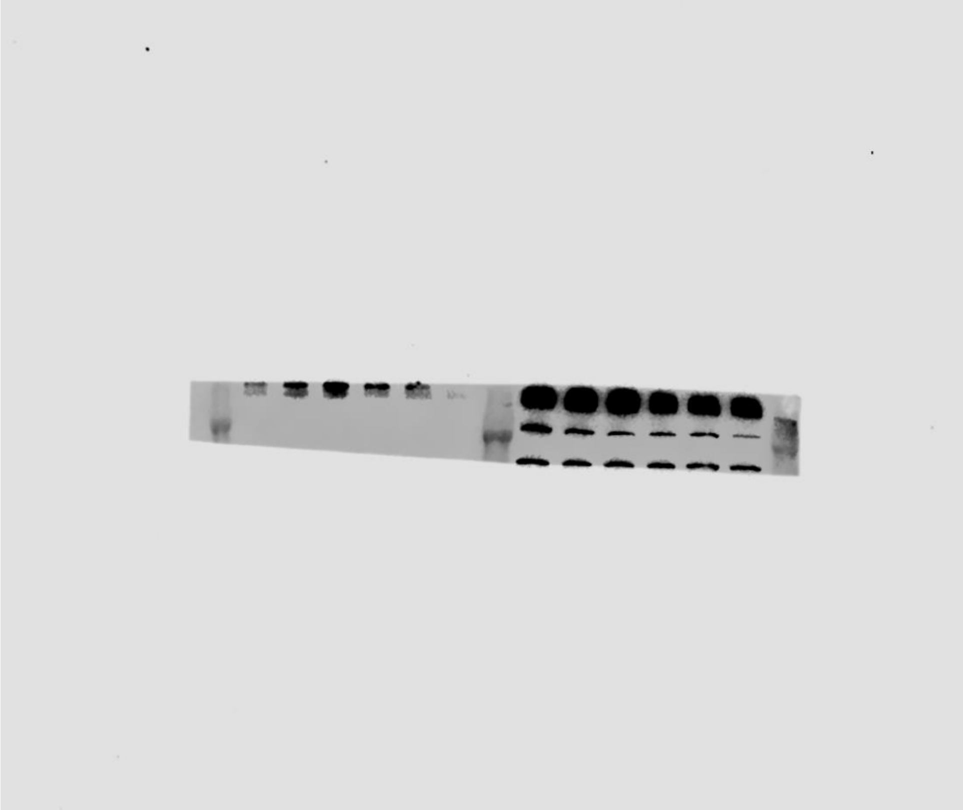

Fig. 6D (Right) anti-ACACA

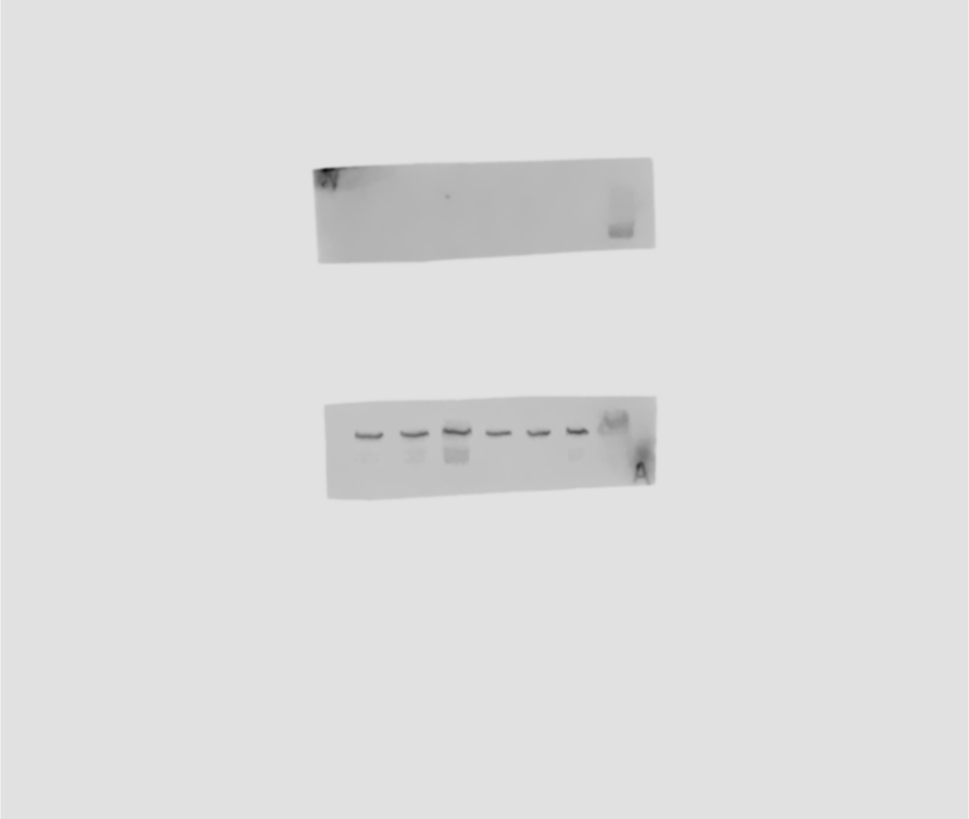

Fig. 6D (Right) anti-p-ACACA Ser80

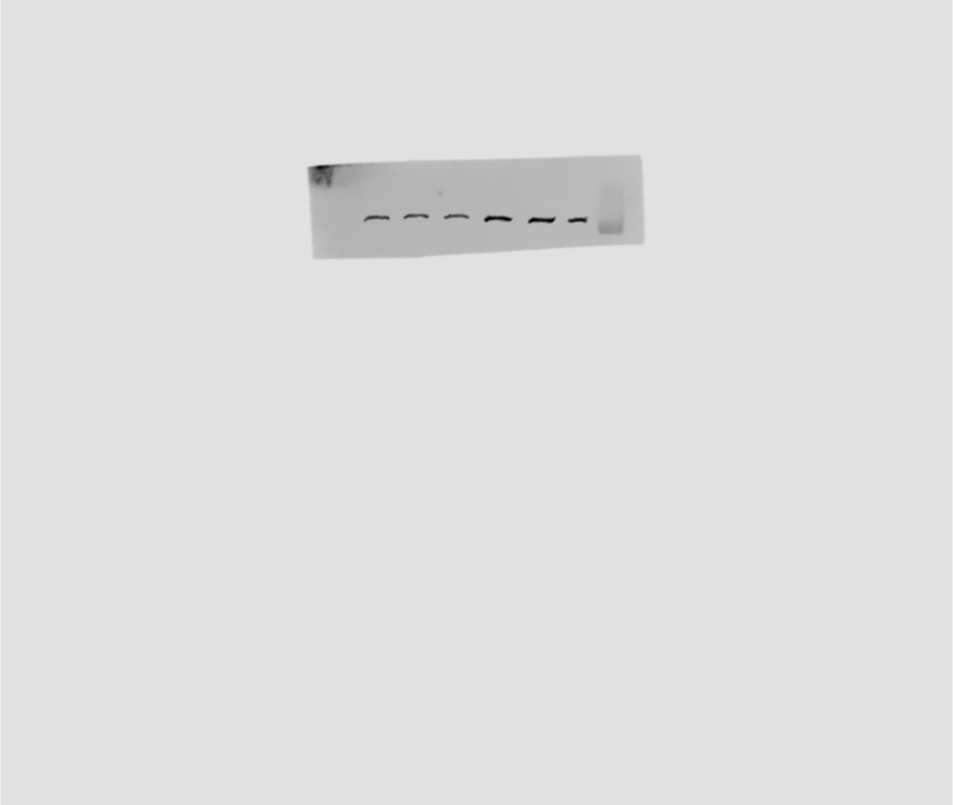

Fig. 6D anti-Tubulin

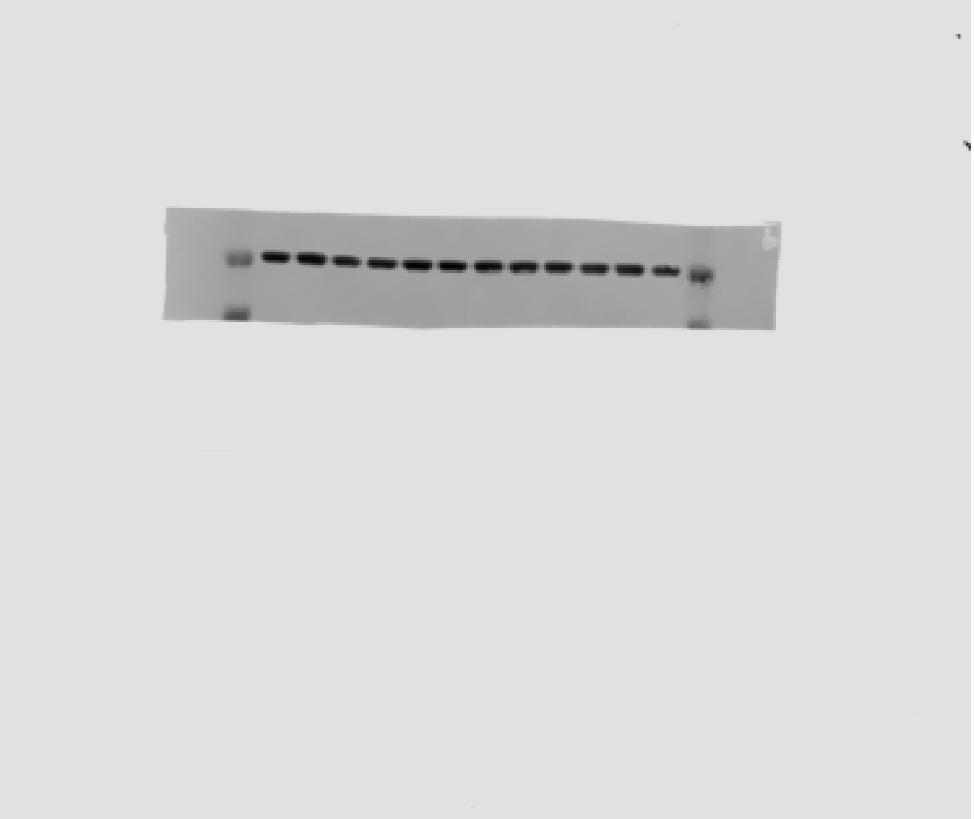

Fig. 6F (Left) anti-p-ACACA Ser80 (Right)

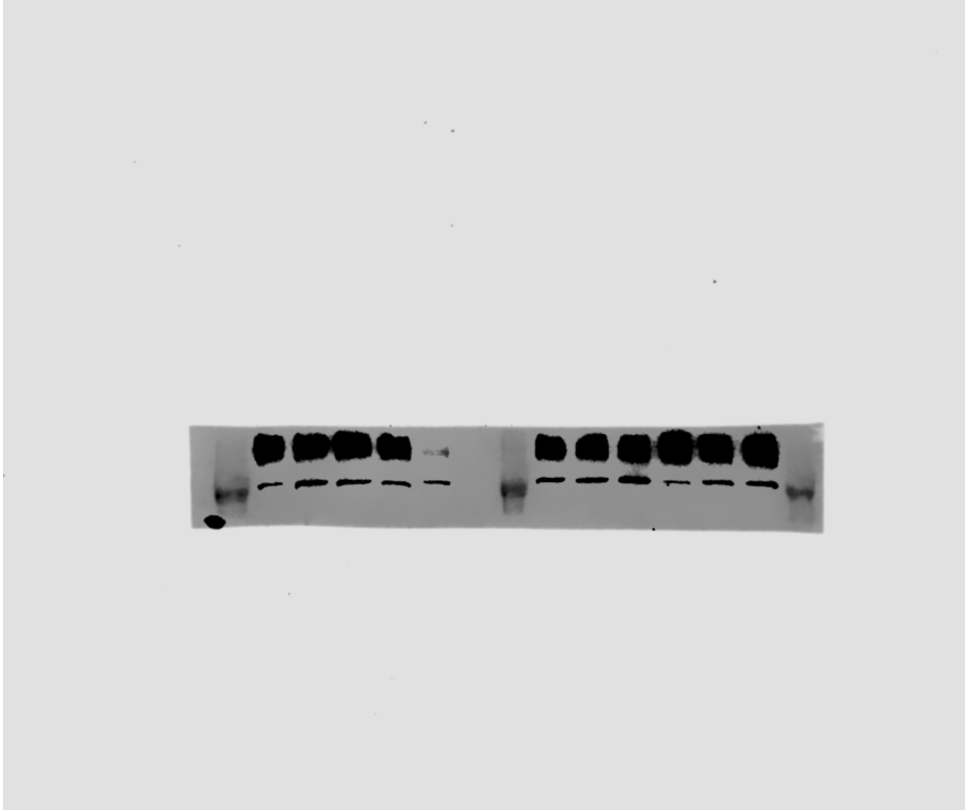

Fig. 6F (Right) anti-p-ACACA Ser80

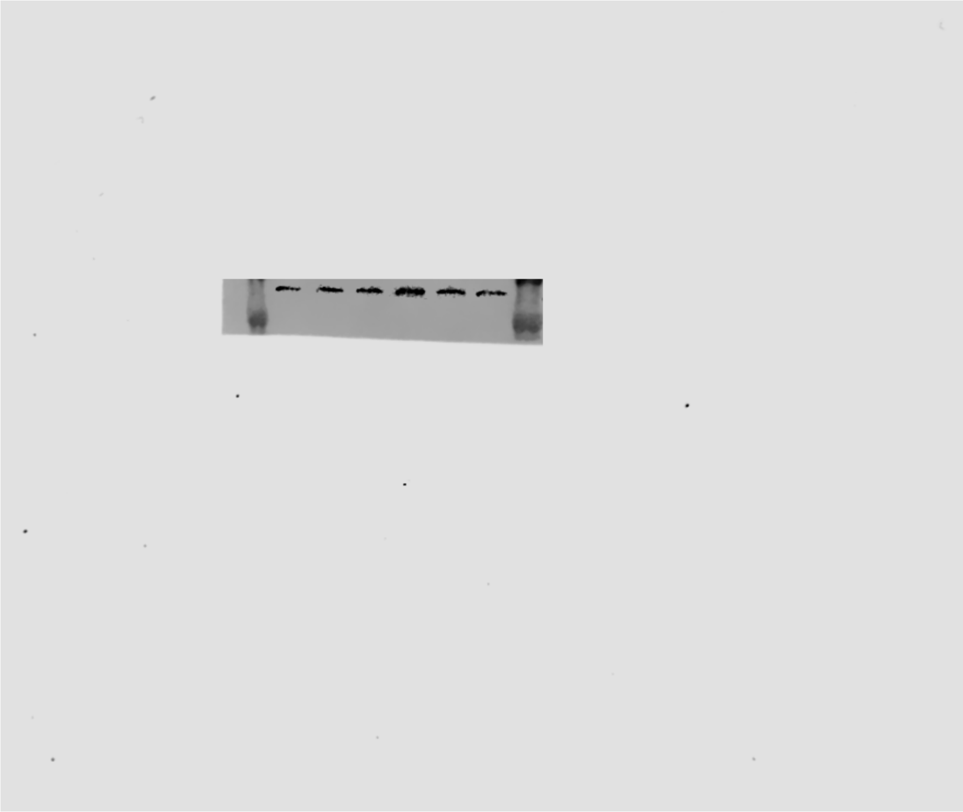

Fig. 6F anti-ACACA

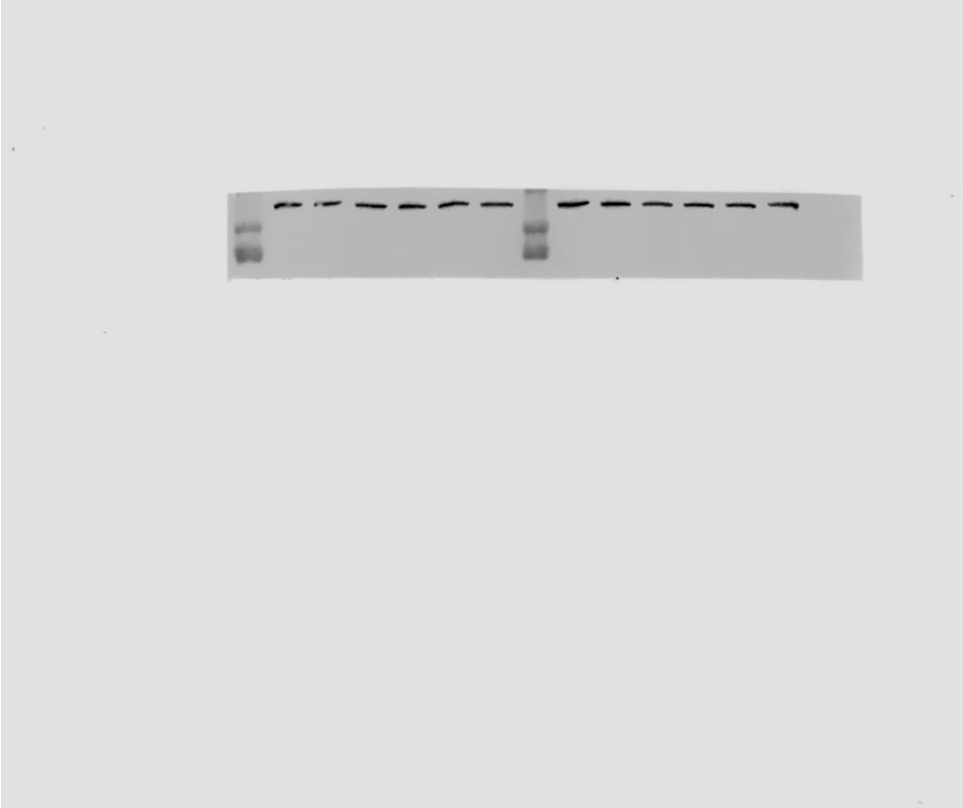

Fig. 6F anti-Tubulin

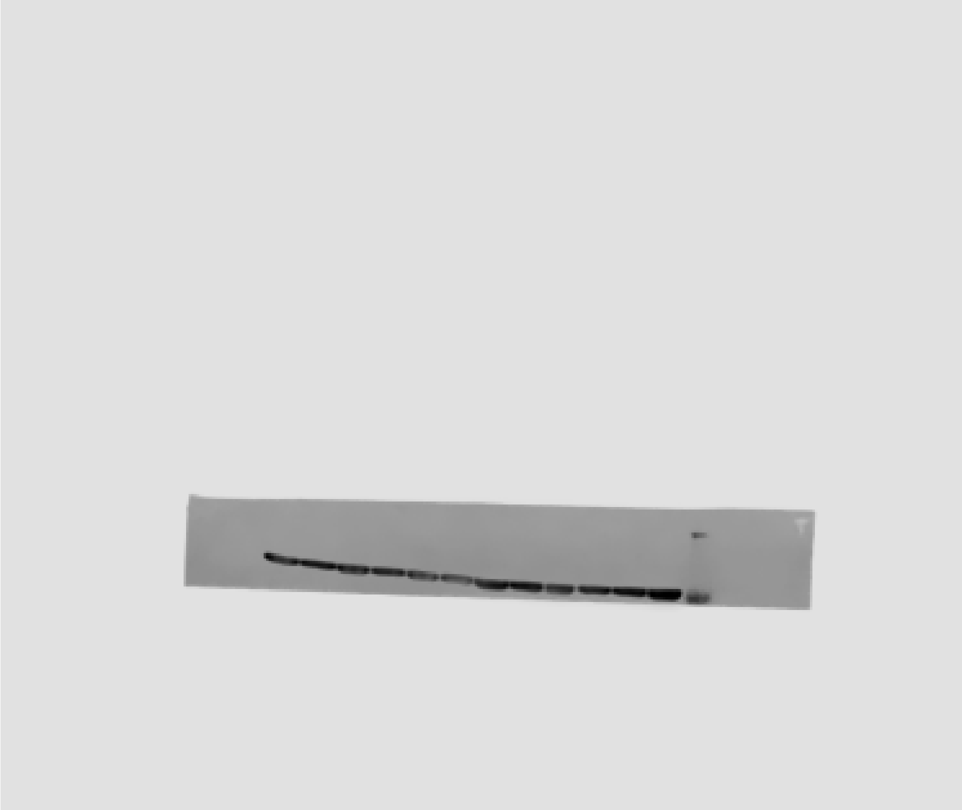

Fig. 6H (Left) anti-PC

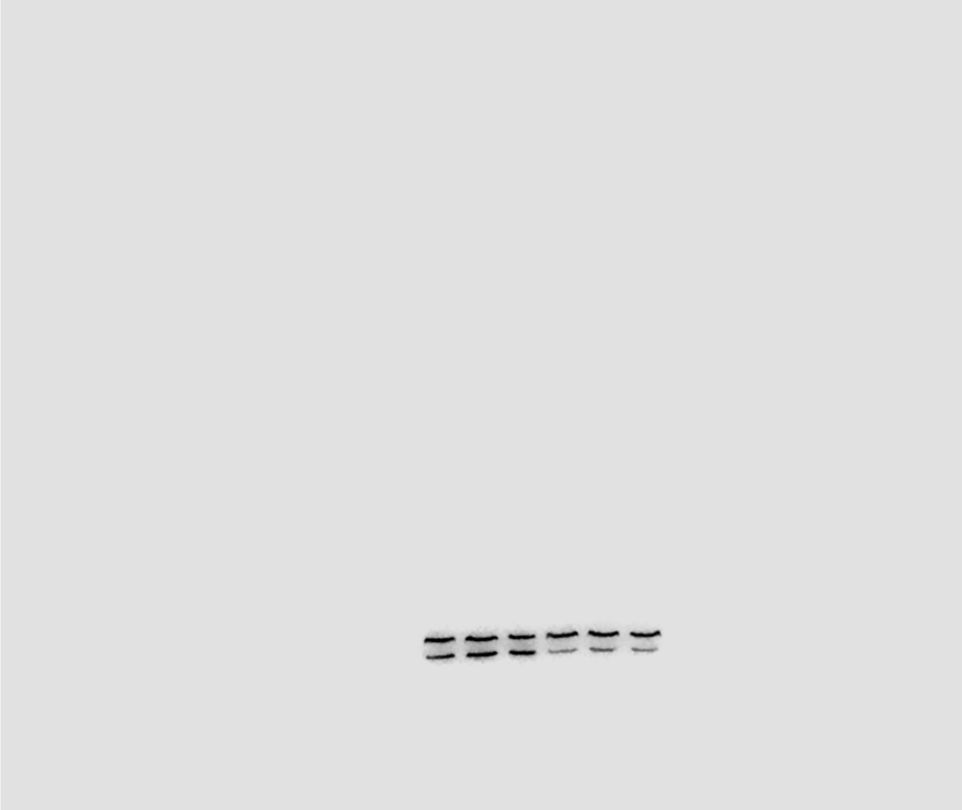

Fig. 6H (Left) anti-Tubulin (Right)

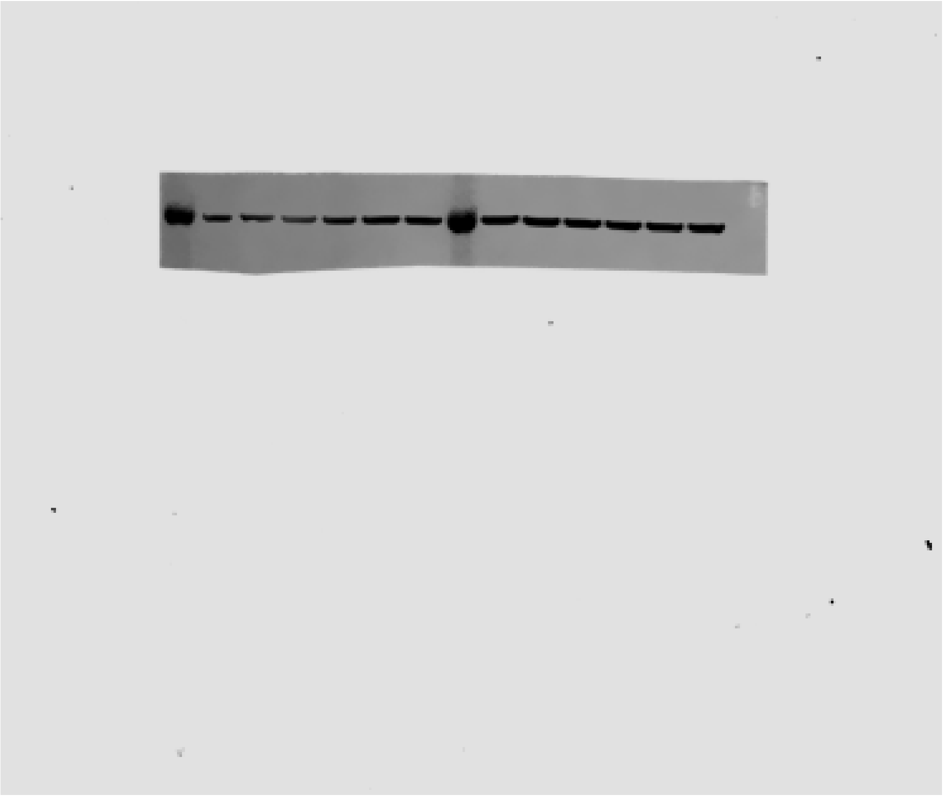

Fig. 6H (Right) anti-PC

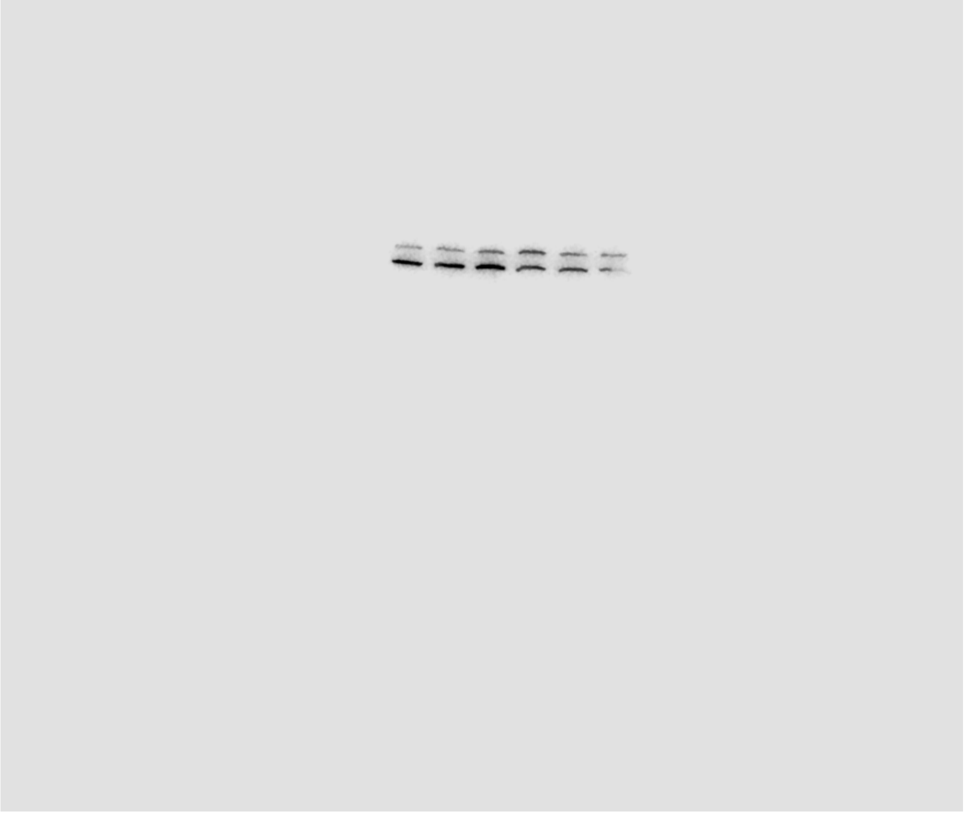

Fig. 6H (Right) anti-Tubulin (Right)

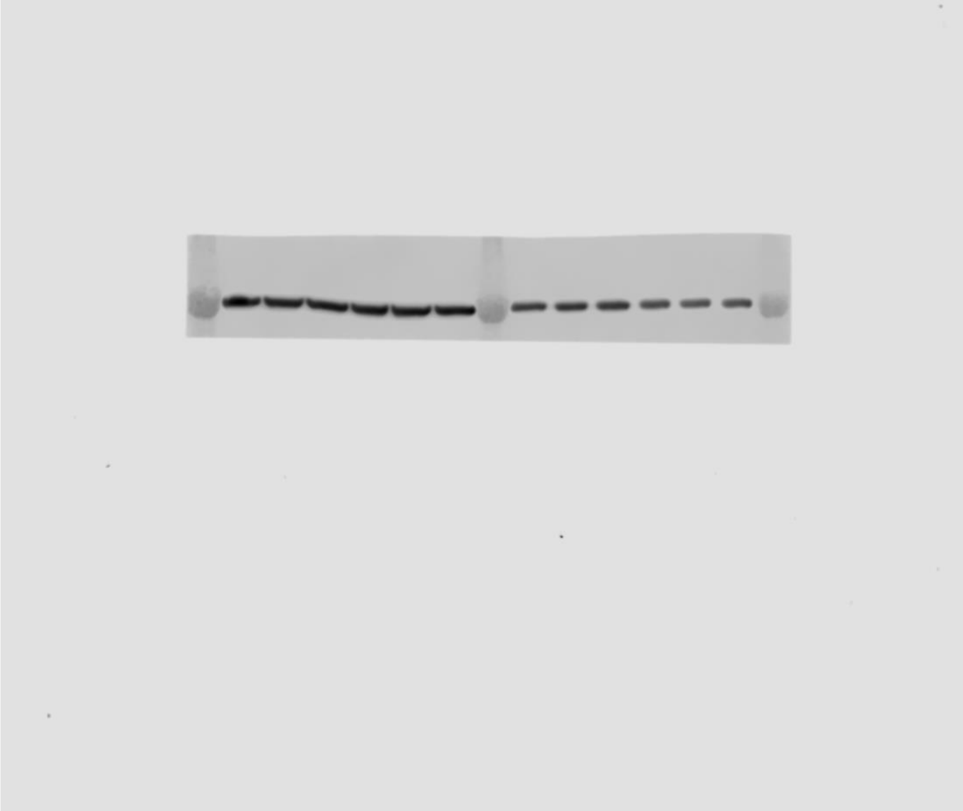

Fig. 6J (Left) anti-PC

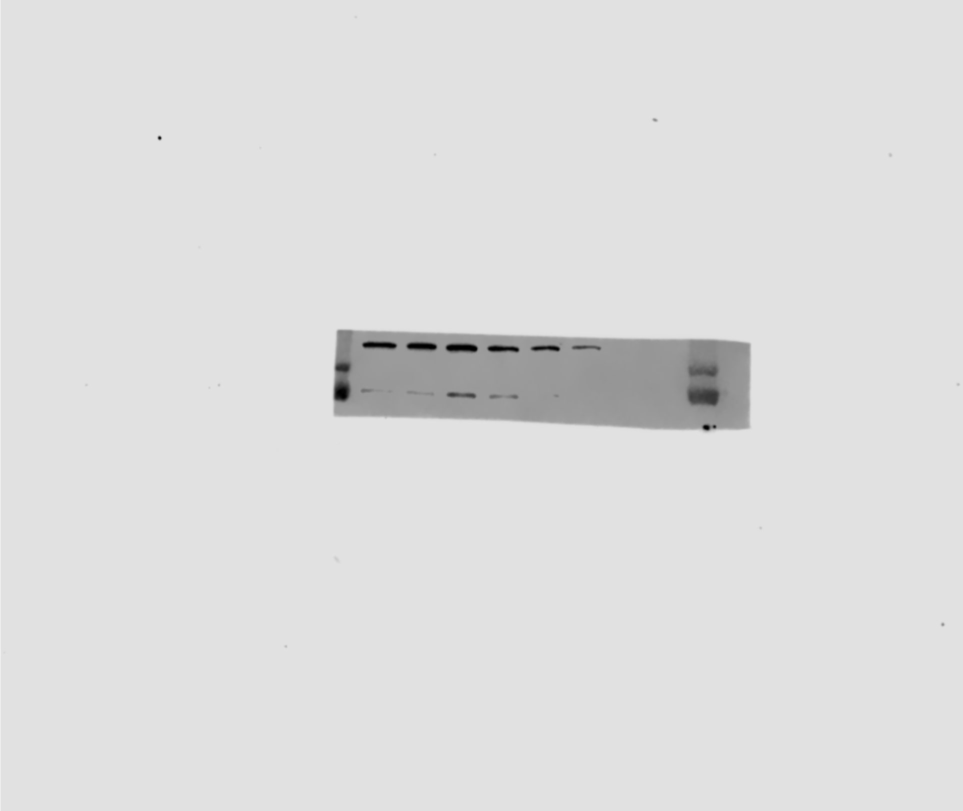

Fig. 6J (Right) anti-PC (Left)

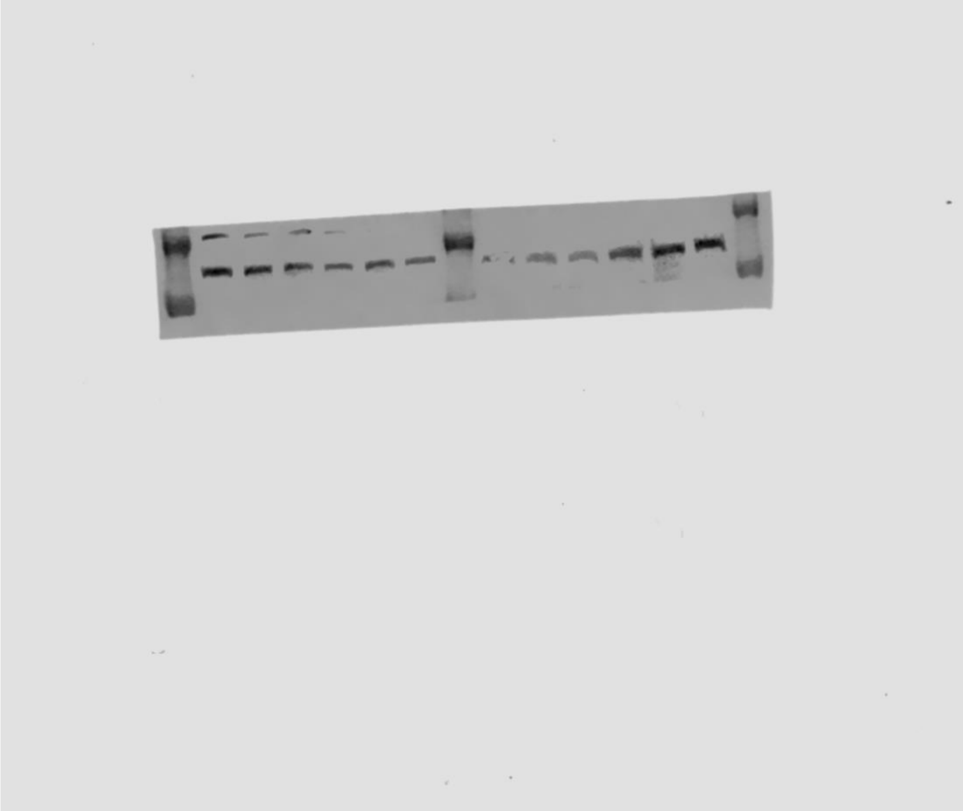

Fig. 6J anti-Tubulin

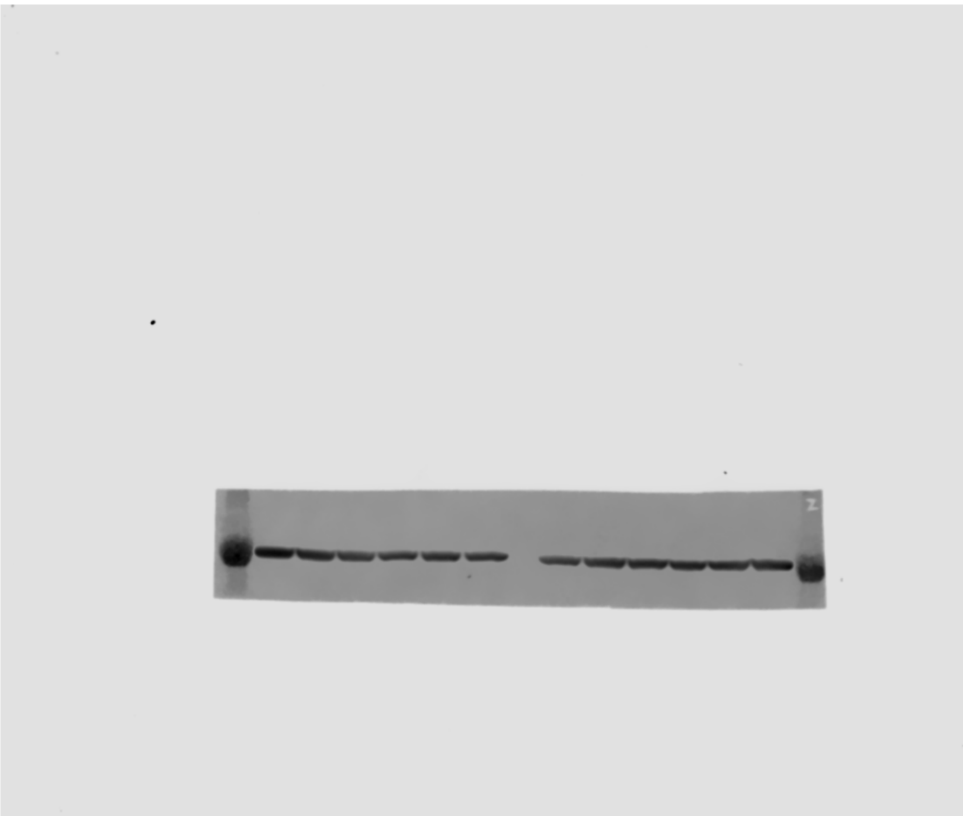

Fig. 6L anti-PC

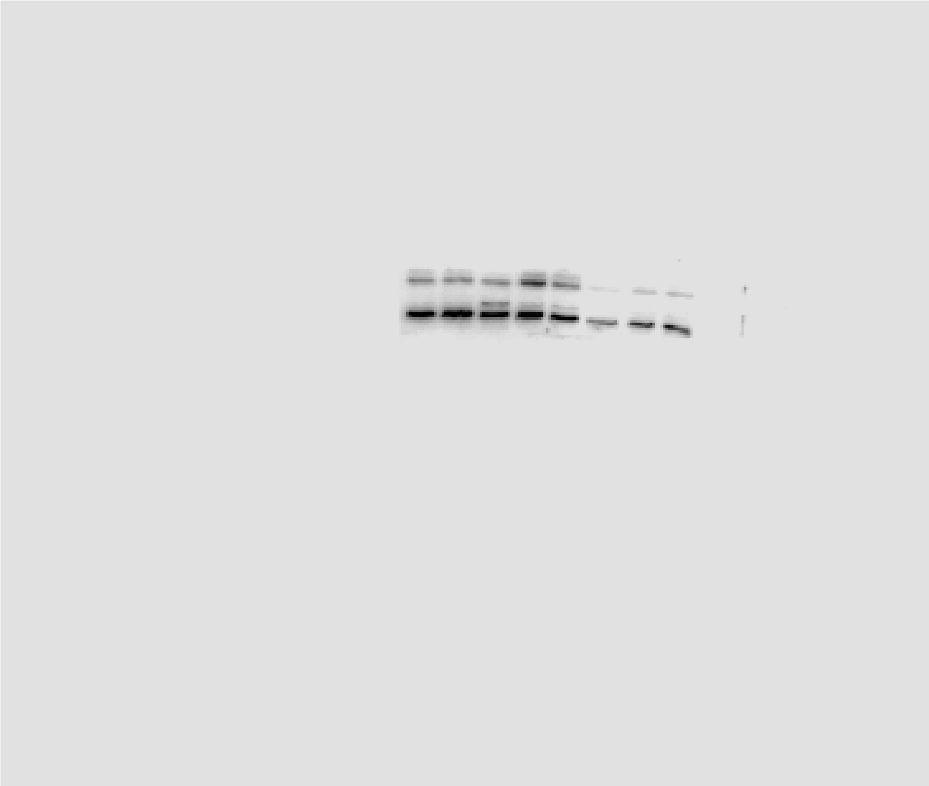

Fig. 6L anti-Tubulin (Right)

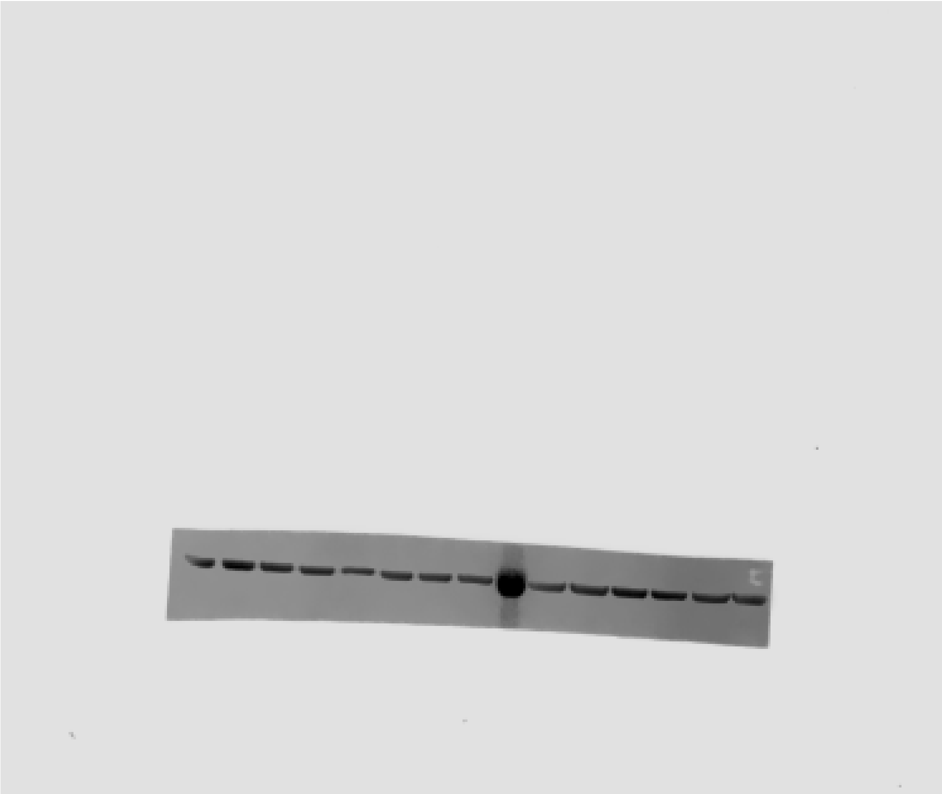

Fig. 6M anti-PC

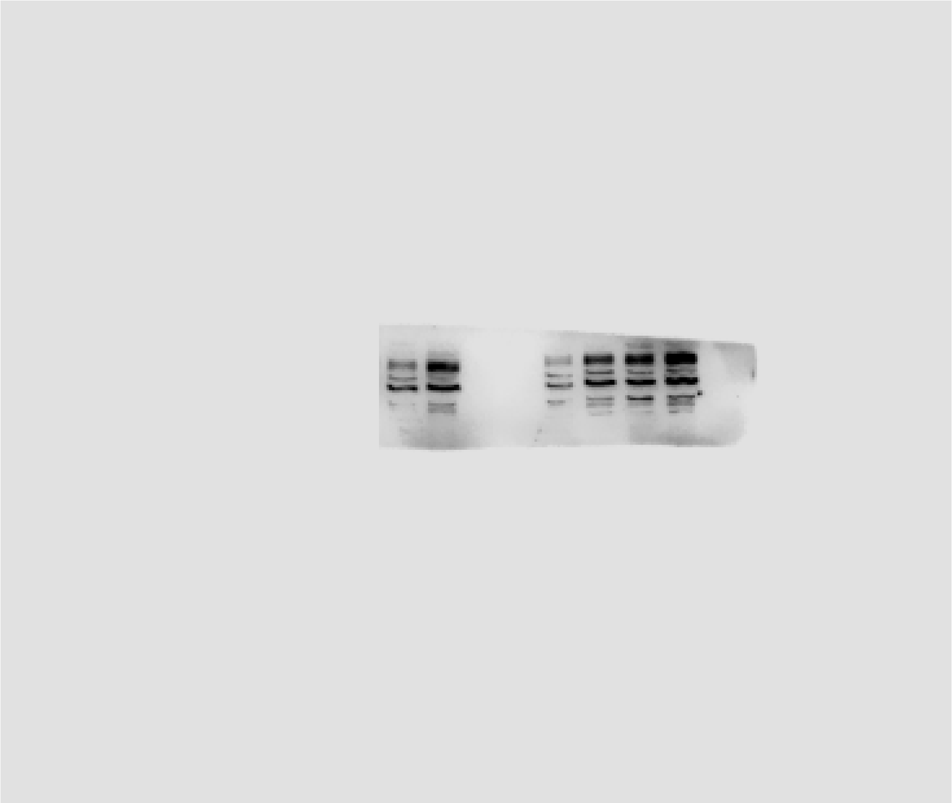

Fig. 6M anti-Tubulin (Left)

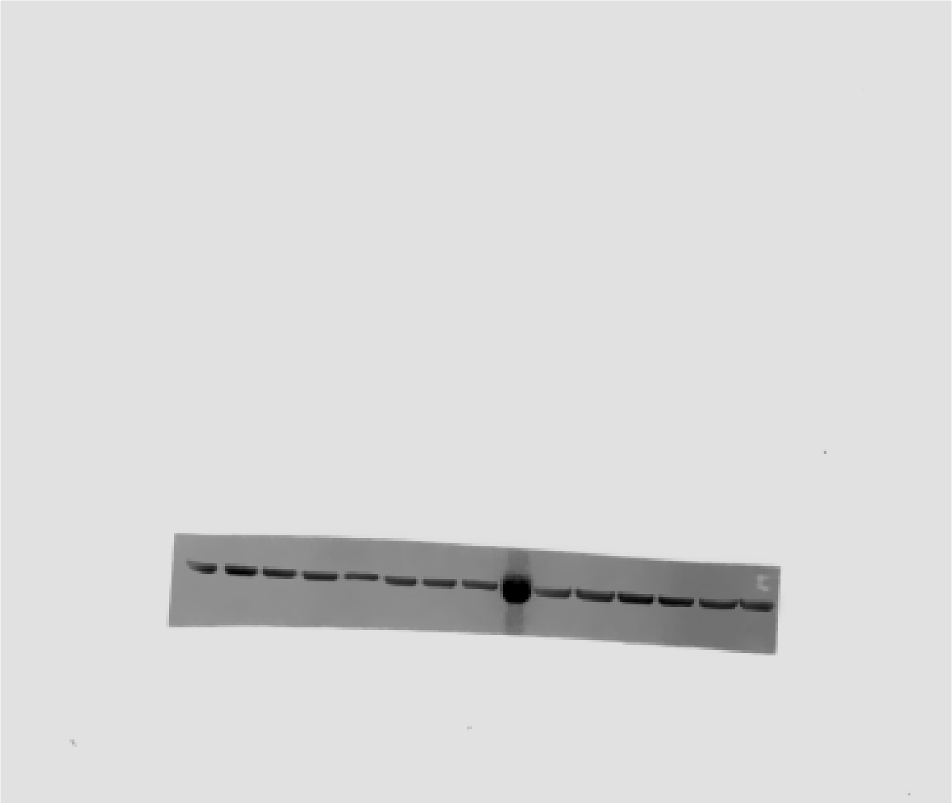

Supplementary Fig. 2K anti-MyHC

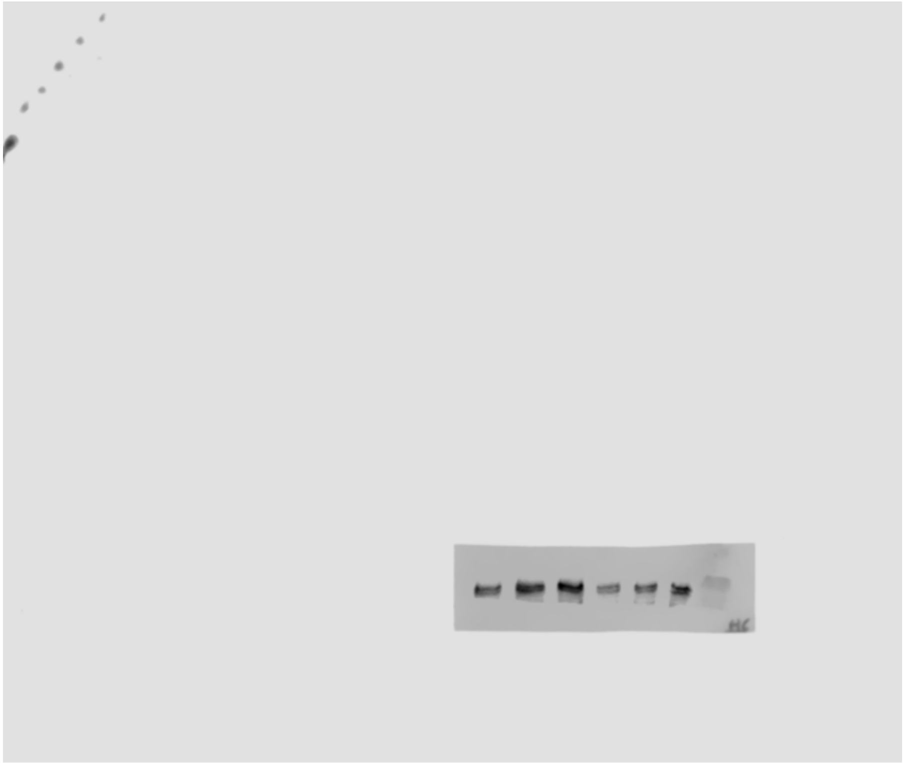

Supplementary Fig. 2K anti-MYOD (Right)

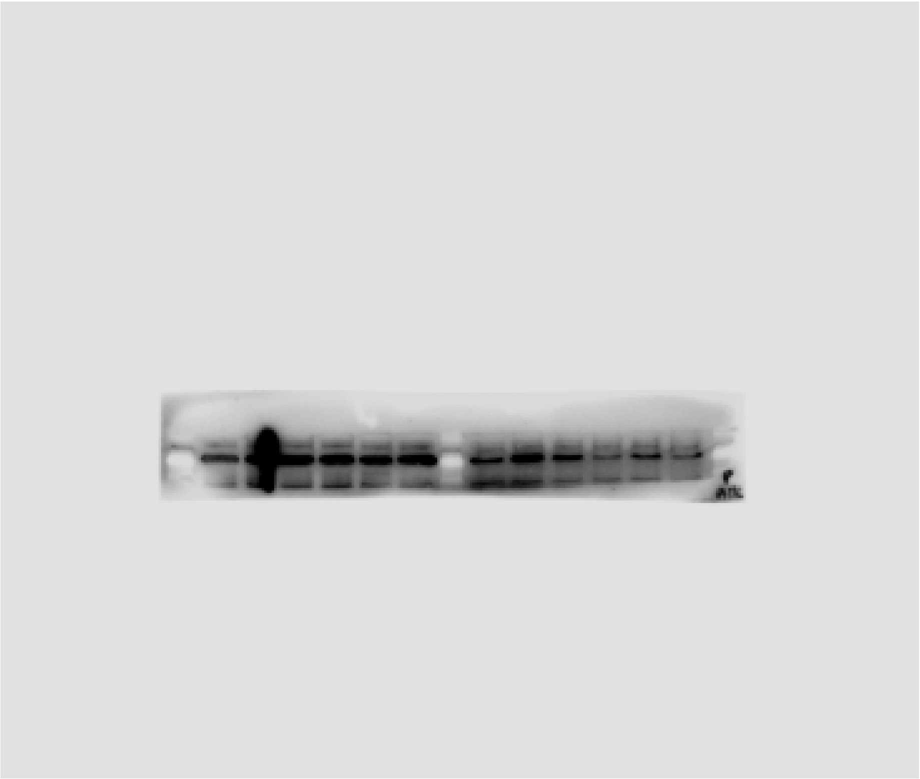

Supplementary Fig. 2K anti-Tubulin (Right)

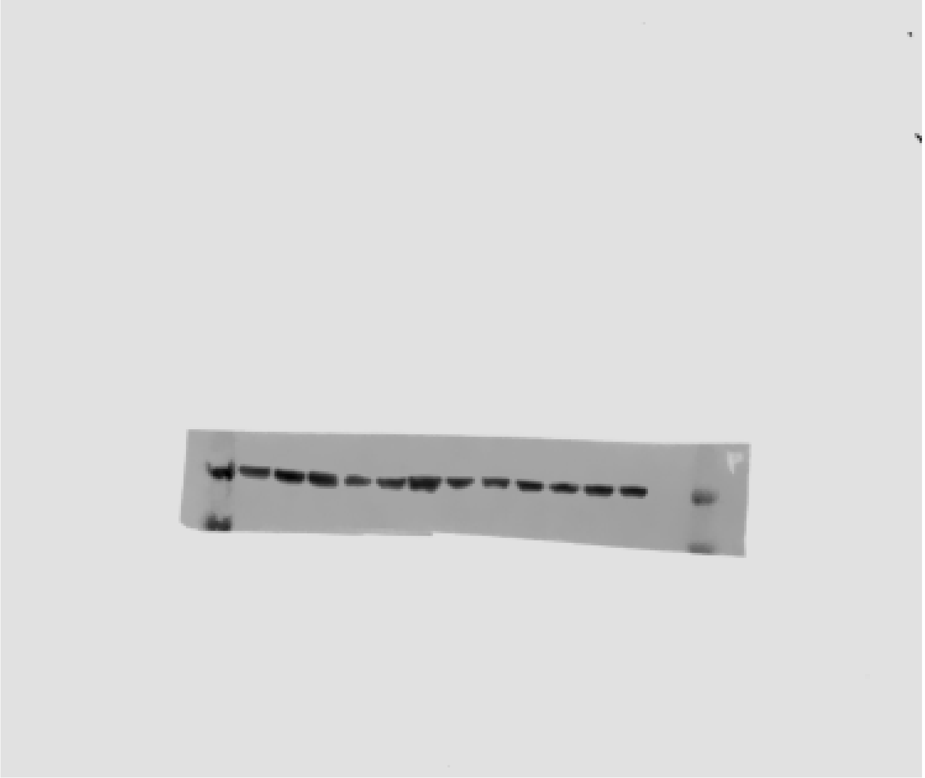

Supplementary Fig. 3J anti-FASN (Left)

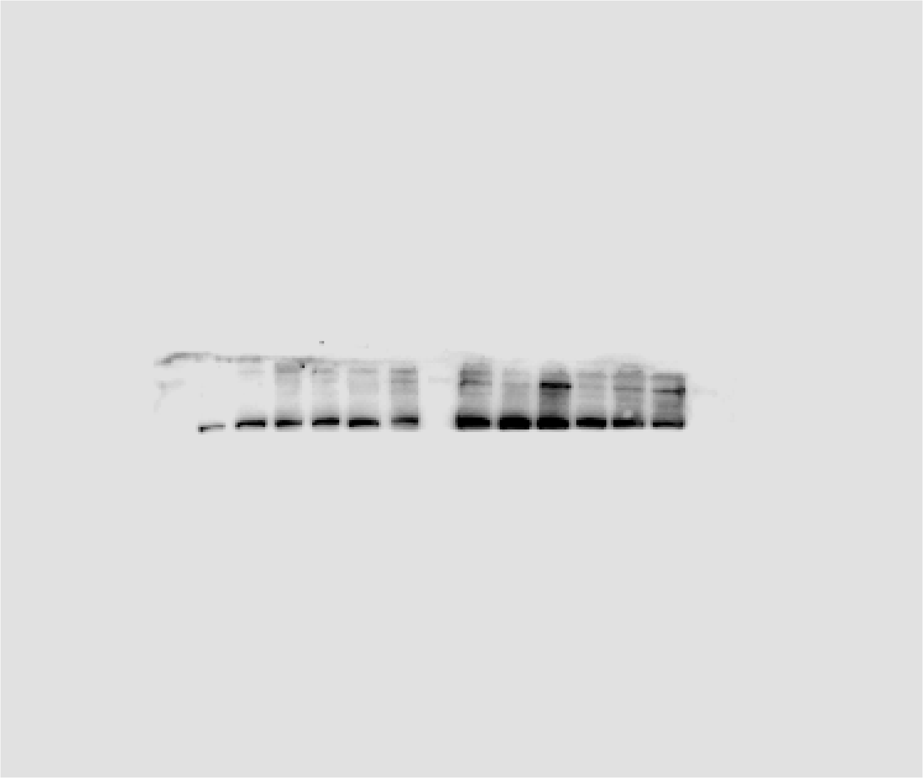

Supplementary Fig. 3J anti-CPT1 (Right)

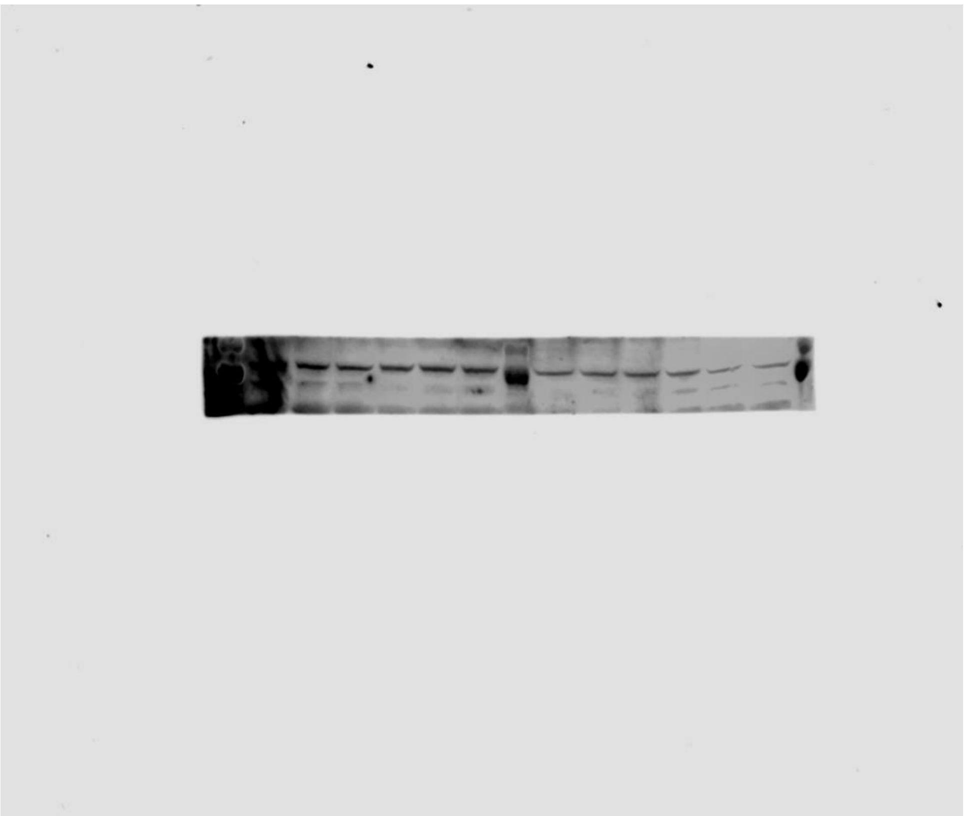

Supplementary Fig. 3J anti-Tubulin (Right)

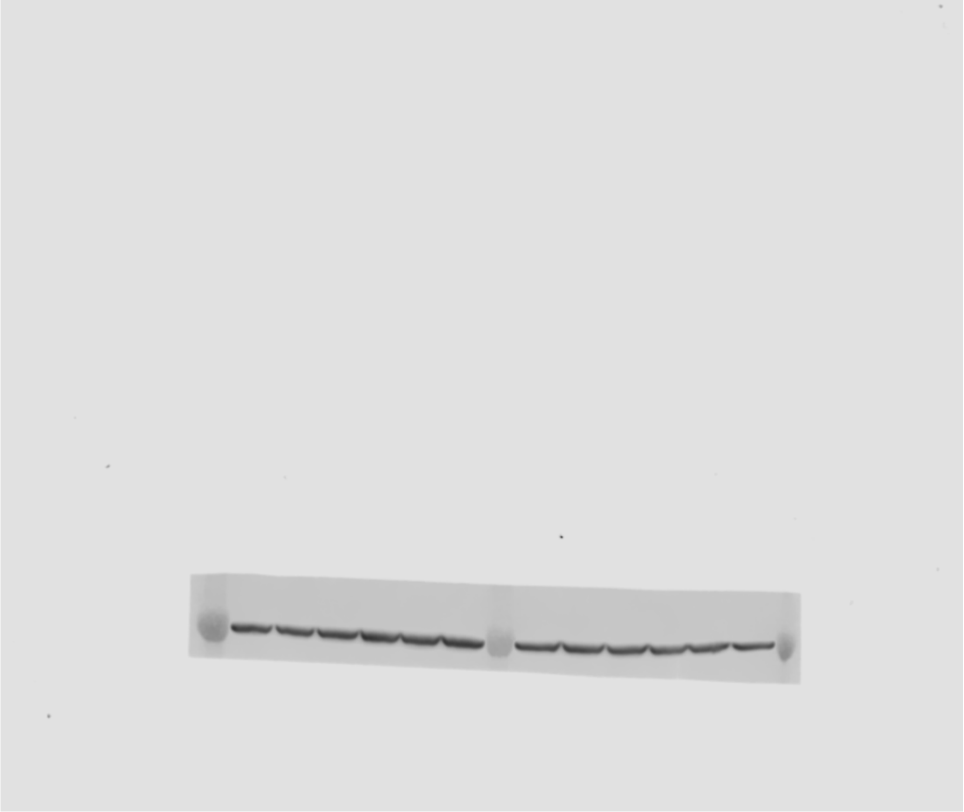

Supplementary Fig. 4K anti-p62 (Left)

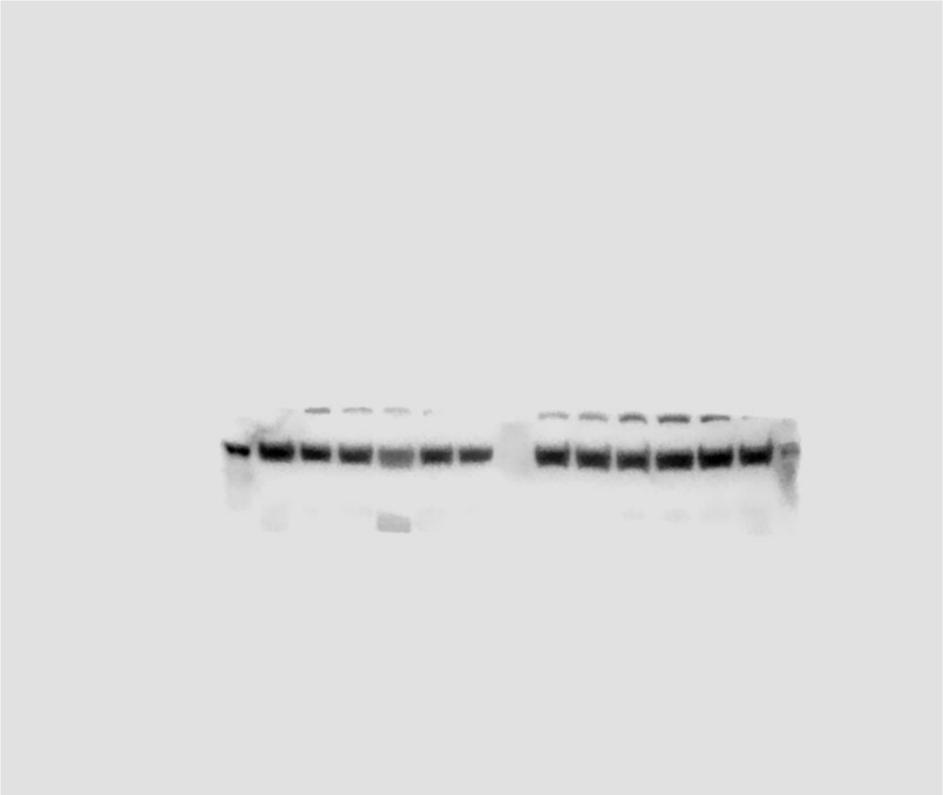

Supplementary Fig. 4K anti-ULK1 (Right)

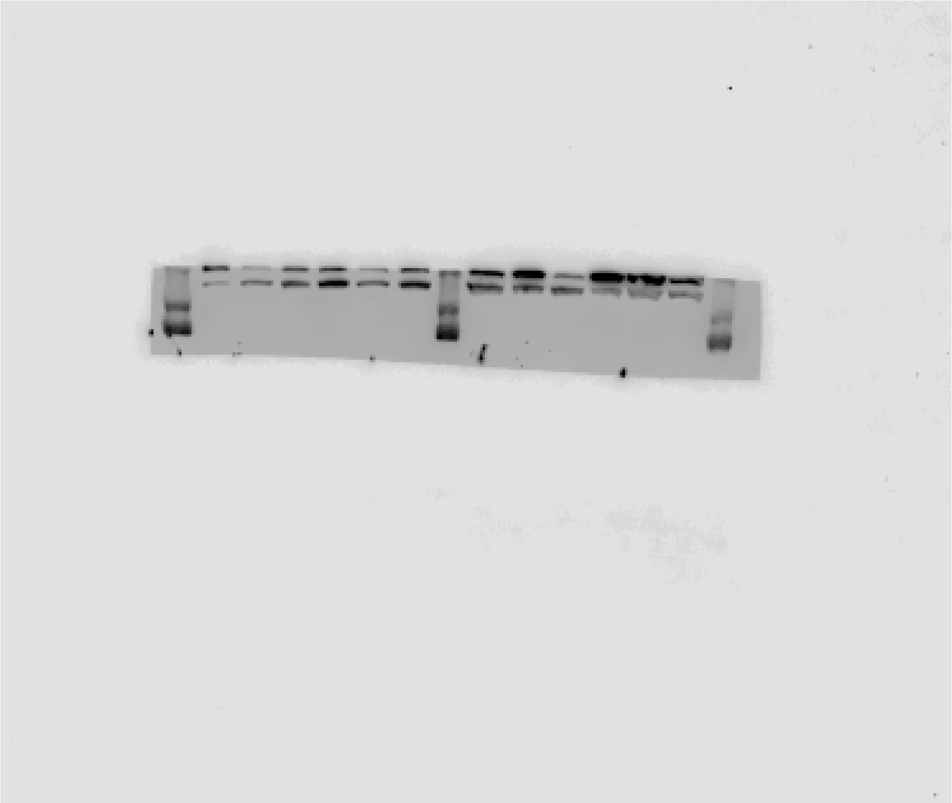

Supplementary Fig. 4K anti-LC3B (Left)

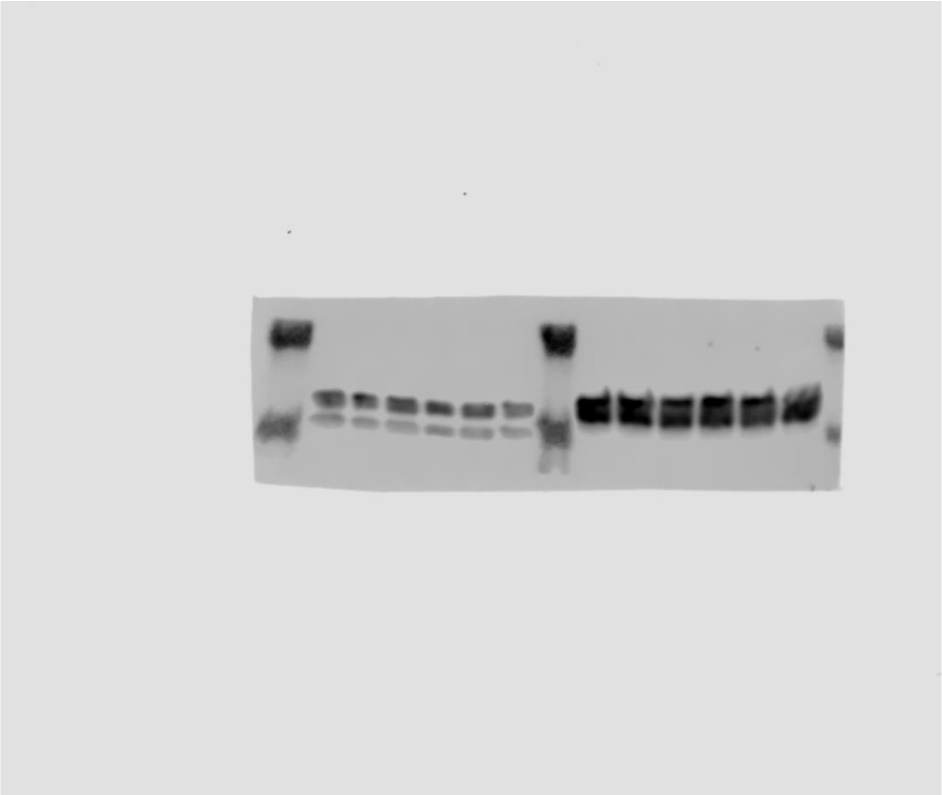

Supplementary Fig. 4K anti-mTOR

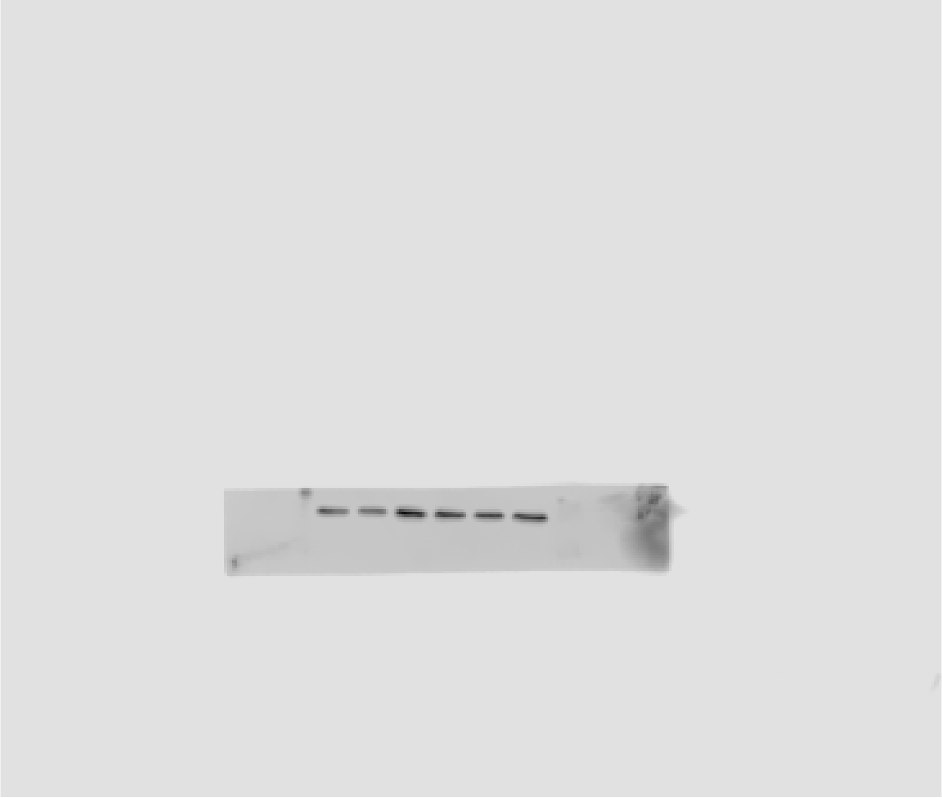

Supplementary Fig. 4K anti-p-mTOR (Left)

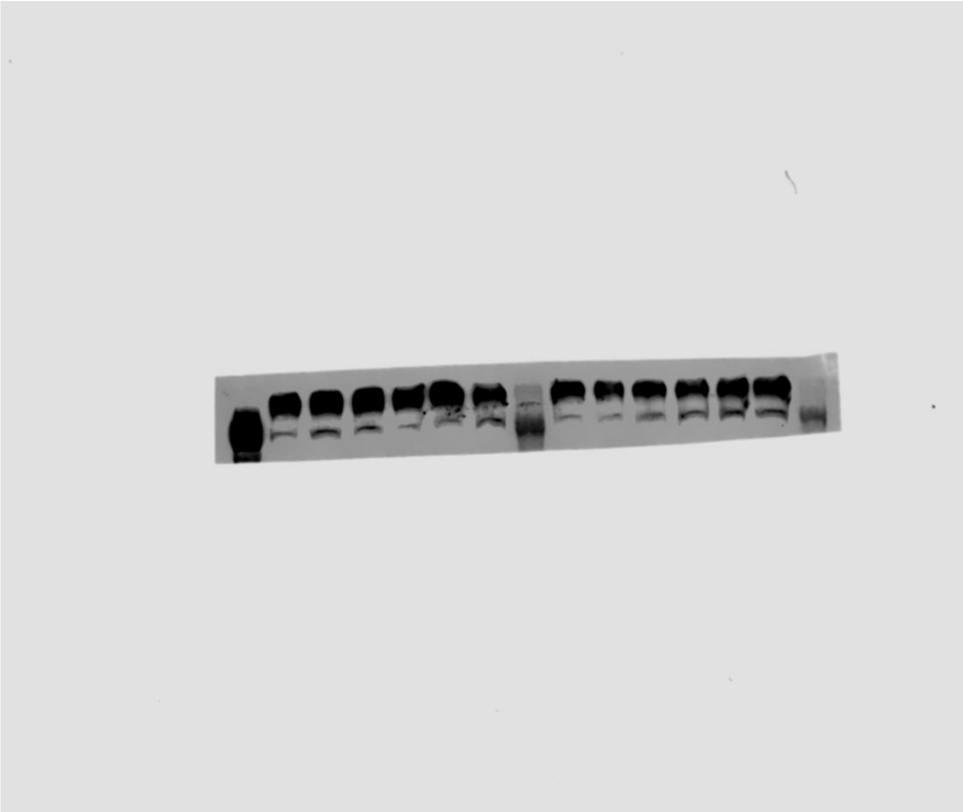

Supplementary Fig. 4K anti-Tubulin (Right)

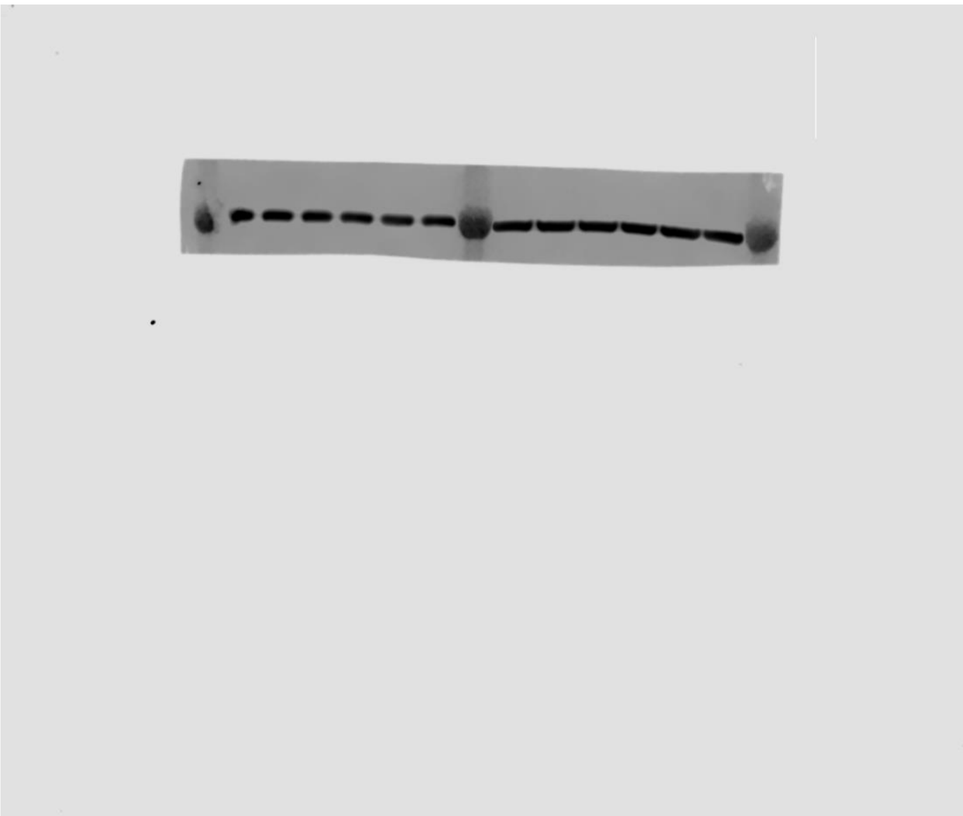

Supplementary Fig. 4K anti-Ubiquitin (Left)

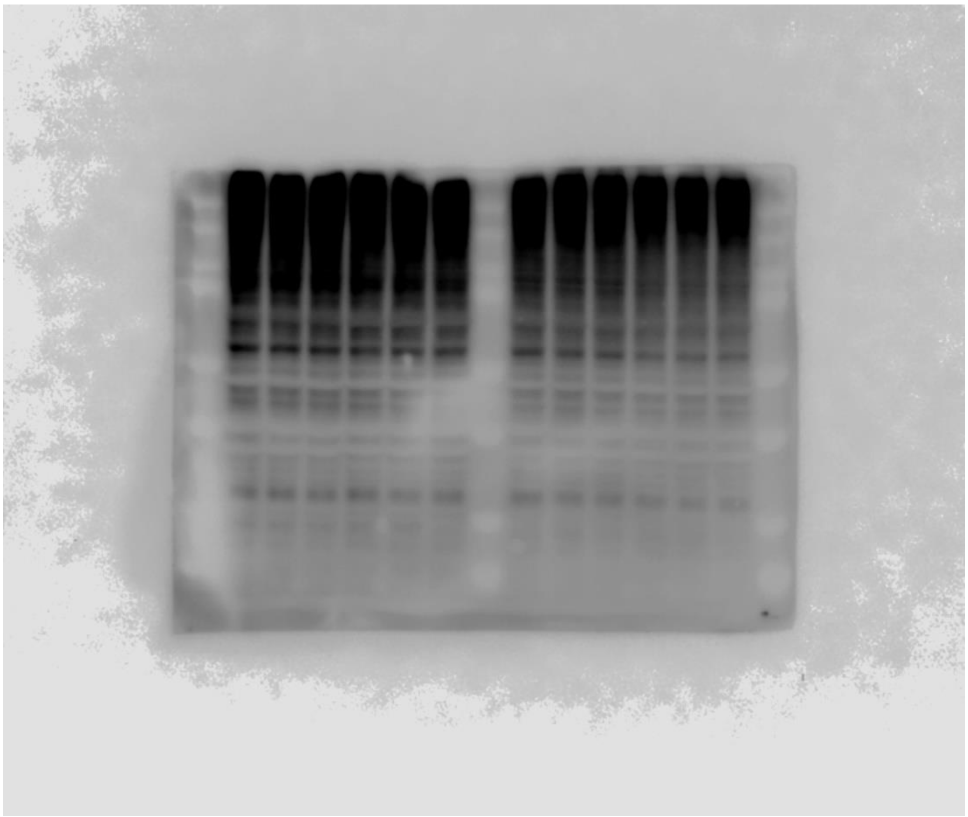

Supplementary Fig. 6G anti-ACACA

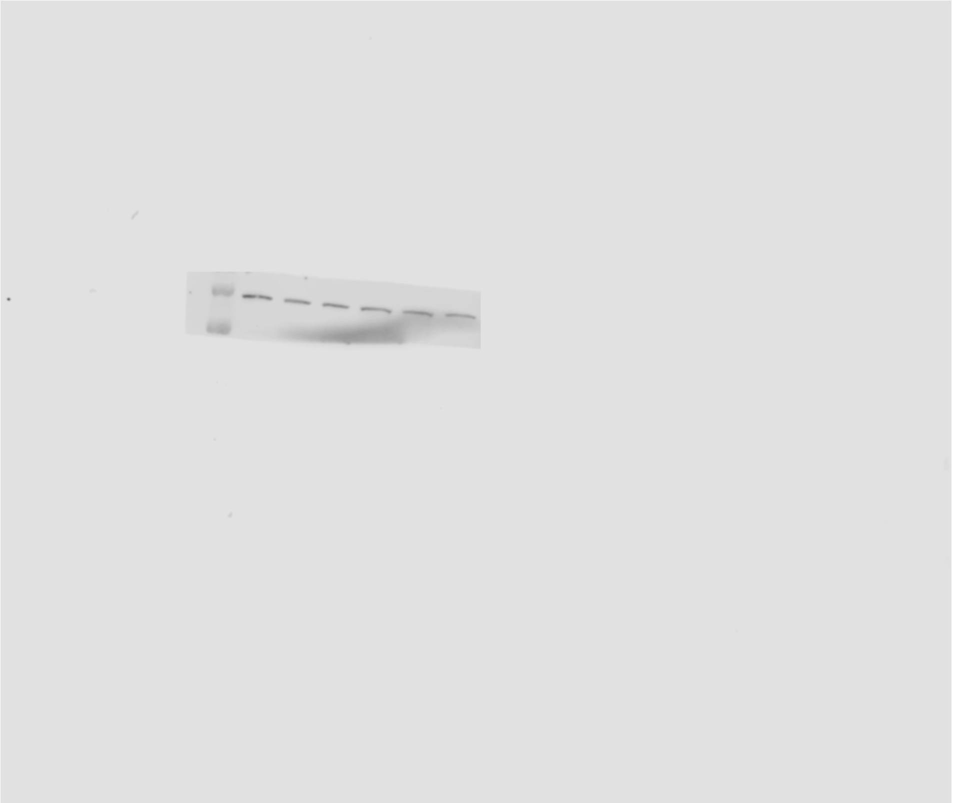

Supplementary Fig. 6G anti-p-ACACA Ser80

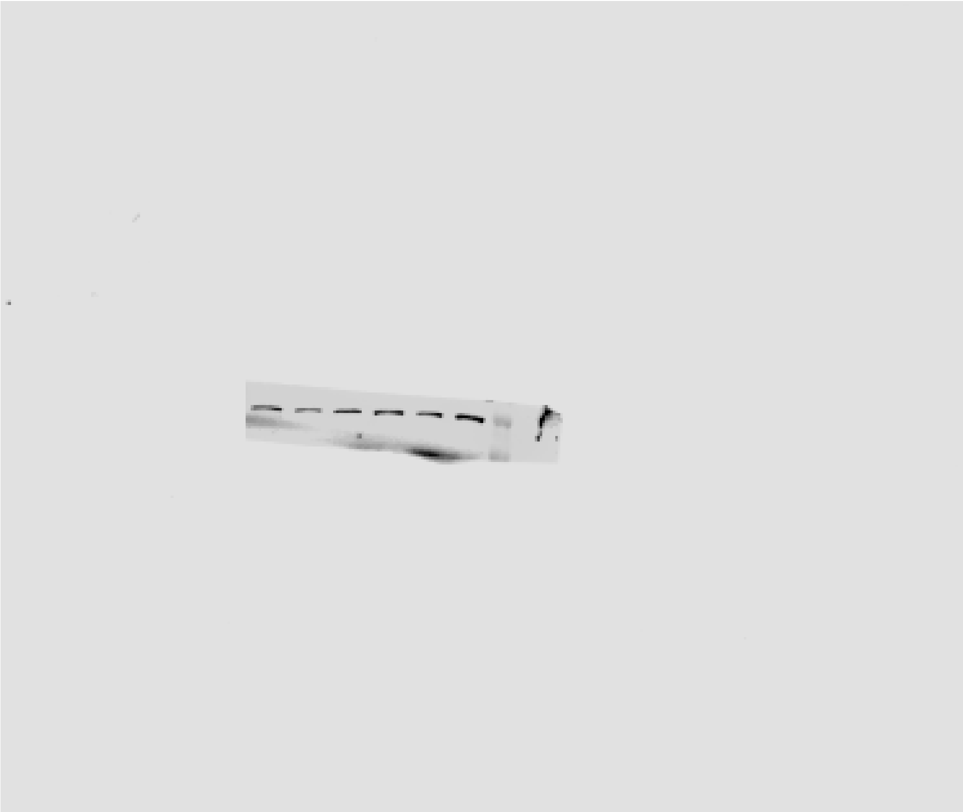

Supplementary Fig. 6G anti-Tubulin (Left)

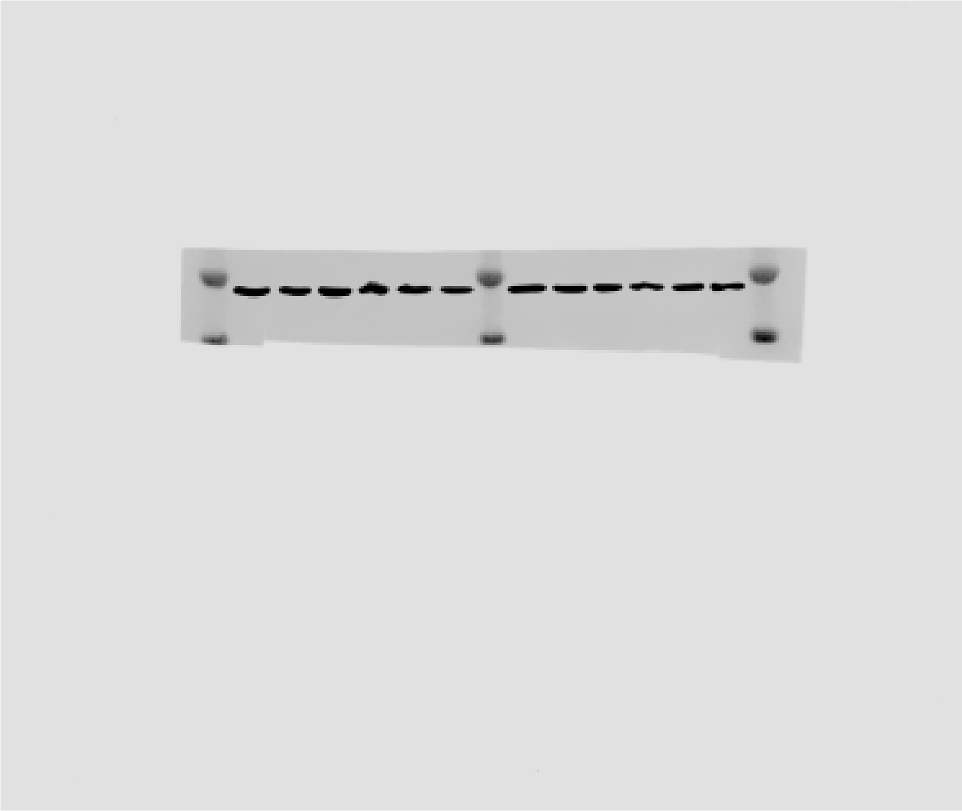

Supplementary Fig. 6I anti-PC (Right)

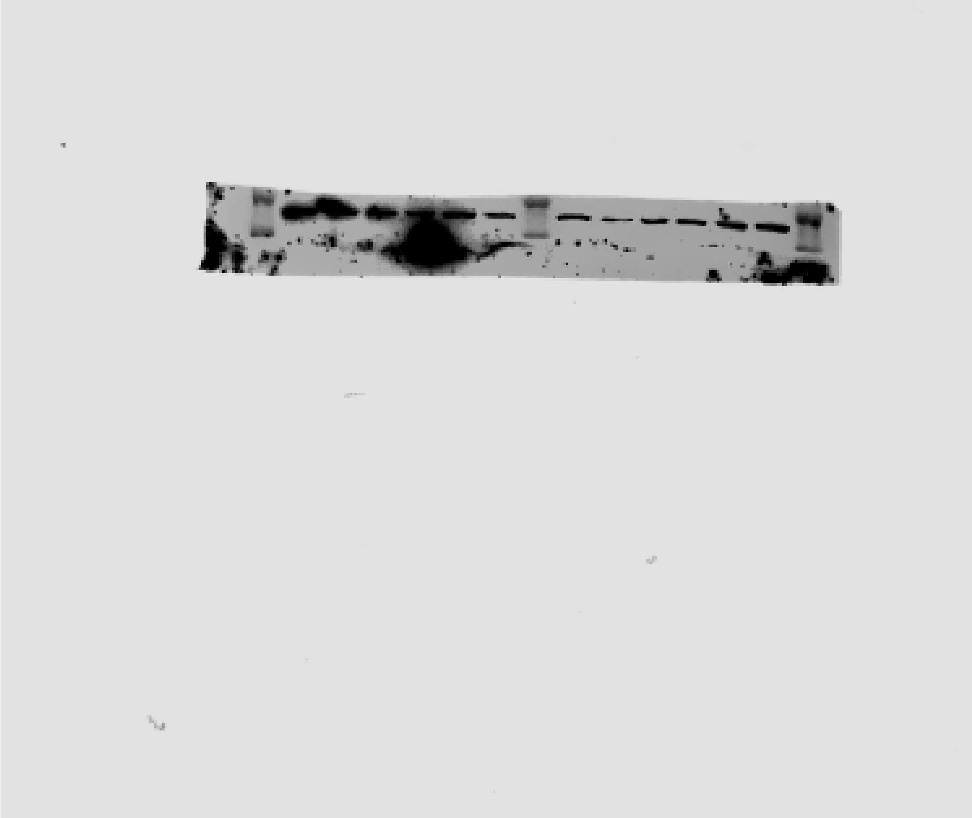

Supplementary Fig. 6I anti-Tubulin (Right)

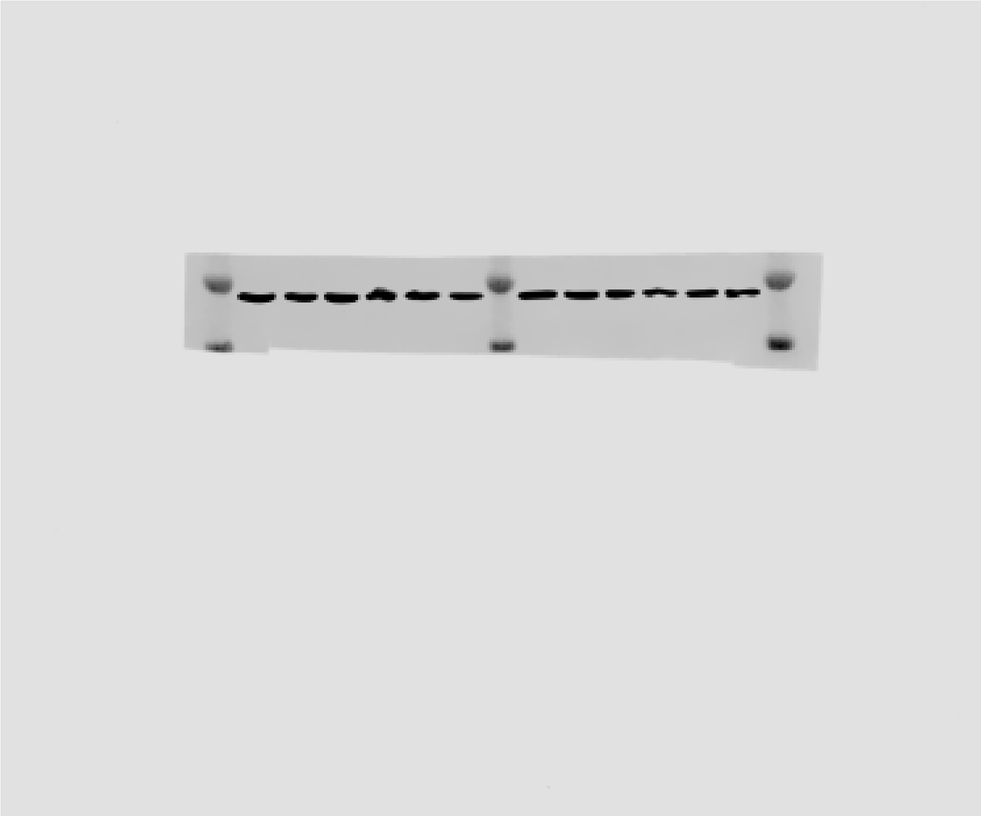

Supplementary Fig. 8B anti-ACACA

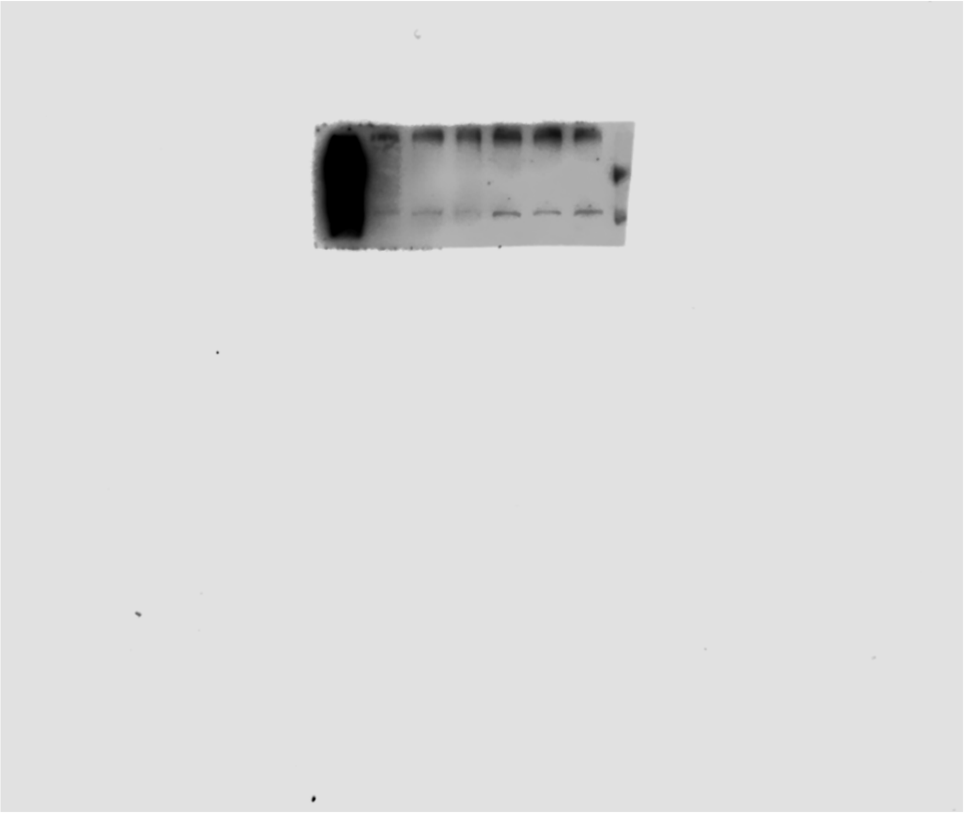

Supplementary Fig. 8B anti-Tubulin (Left)

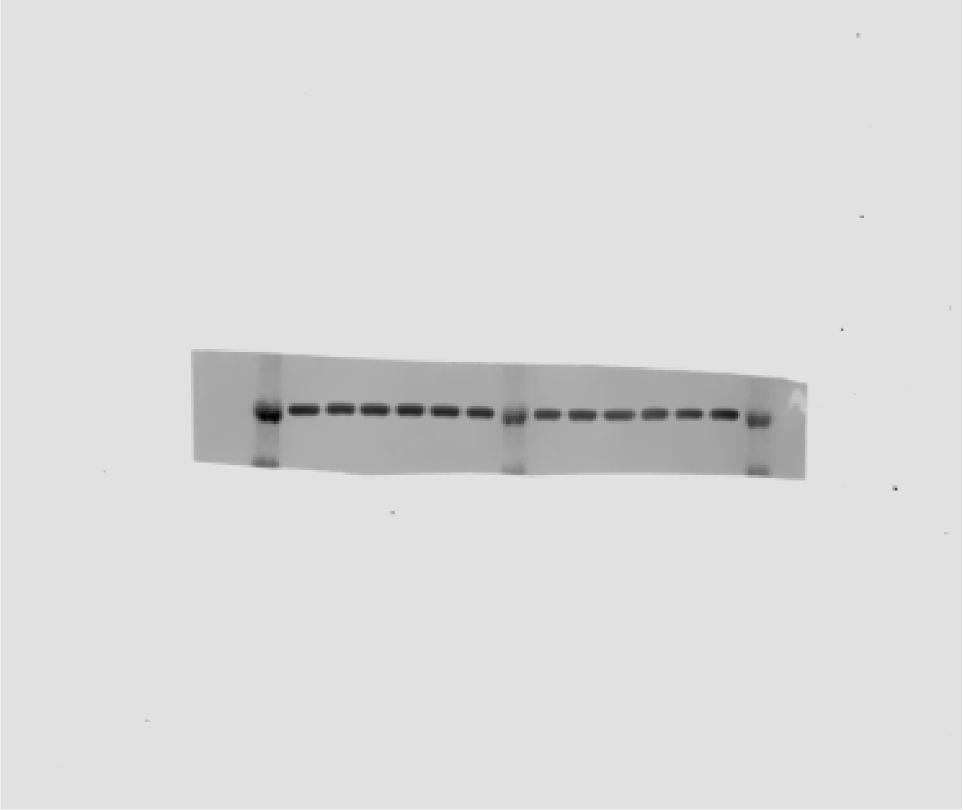

Supplementary Fig. 8L anti-MyHC

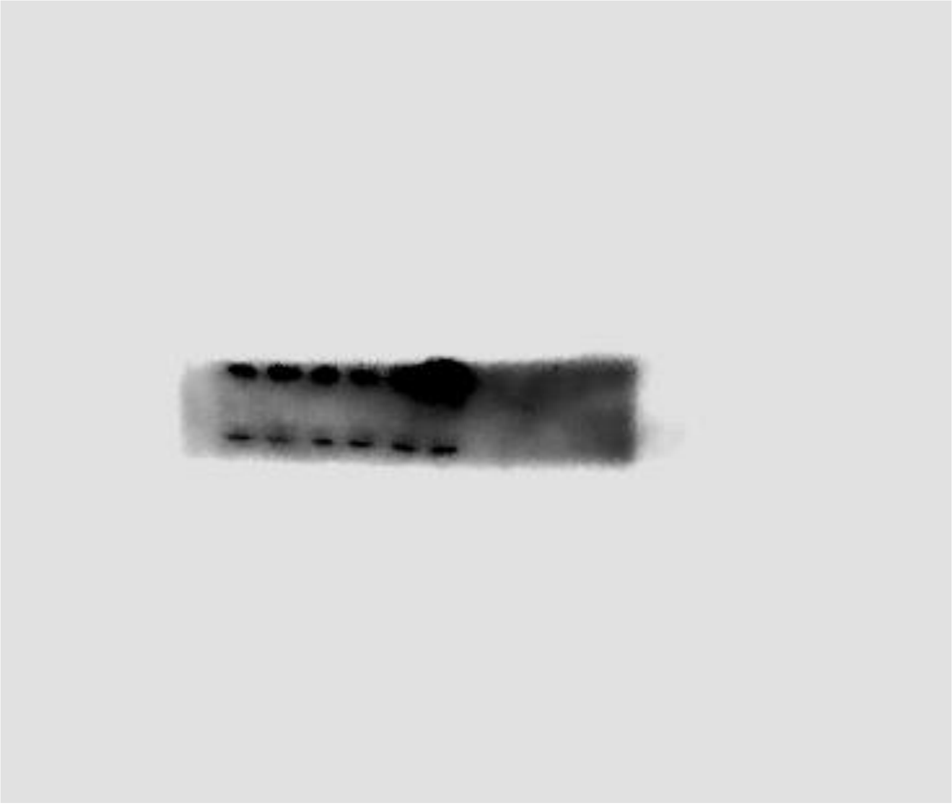

Supplementary Fig. 8L anti-MYOD (Left)

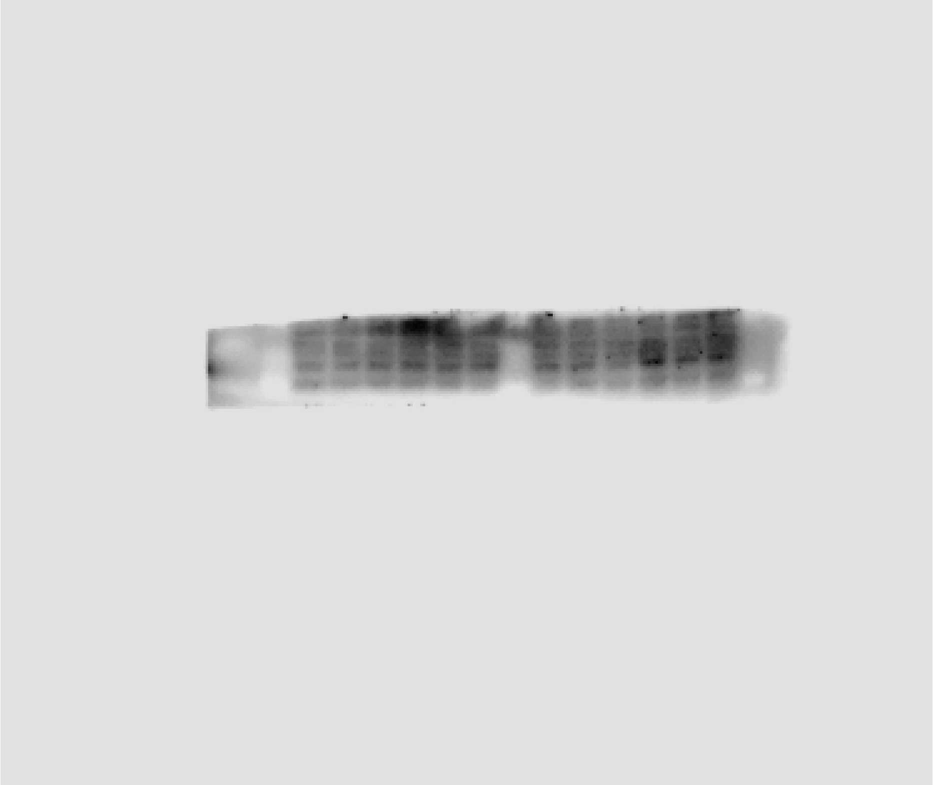

Supplementary Fig. 8L anti-Tubulin (Right)

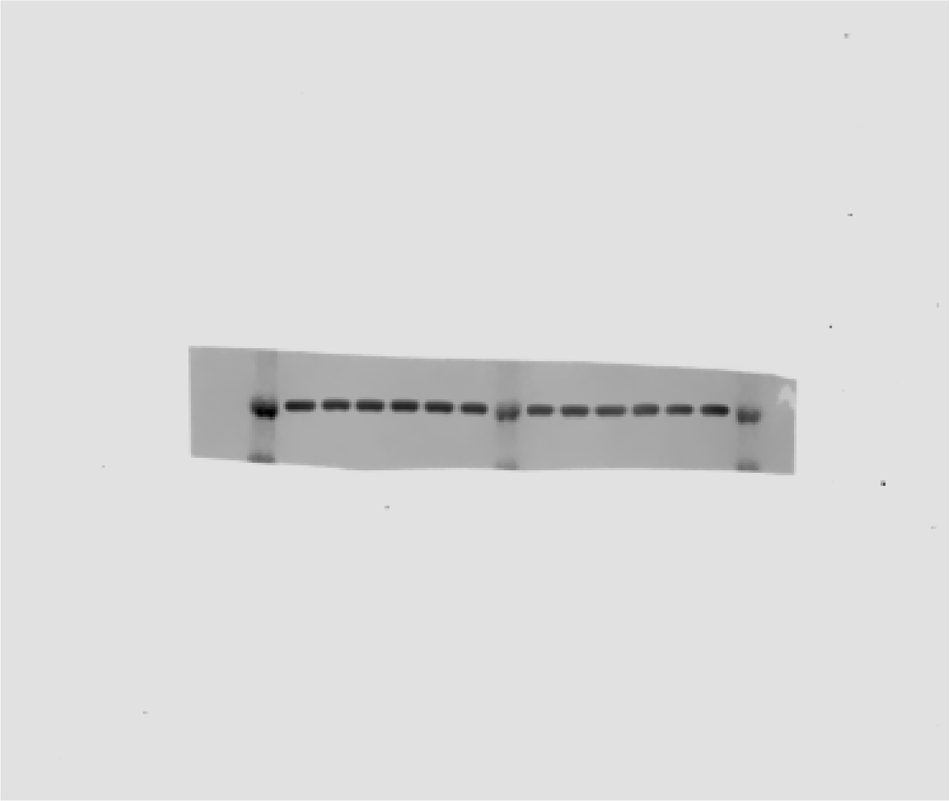

Supplementary Fig. 10B anti-PC (Right)

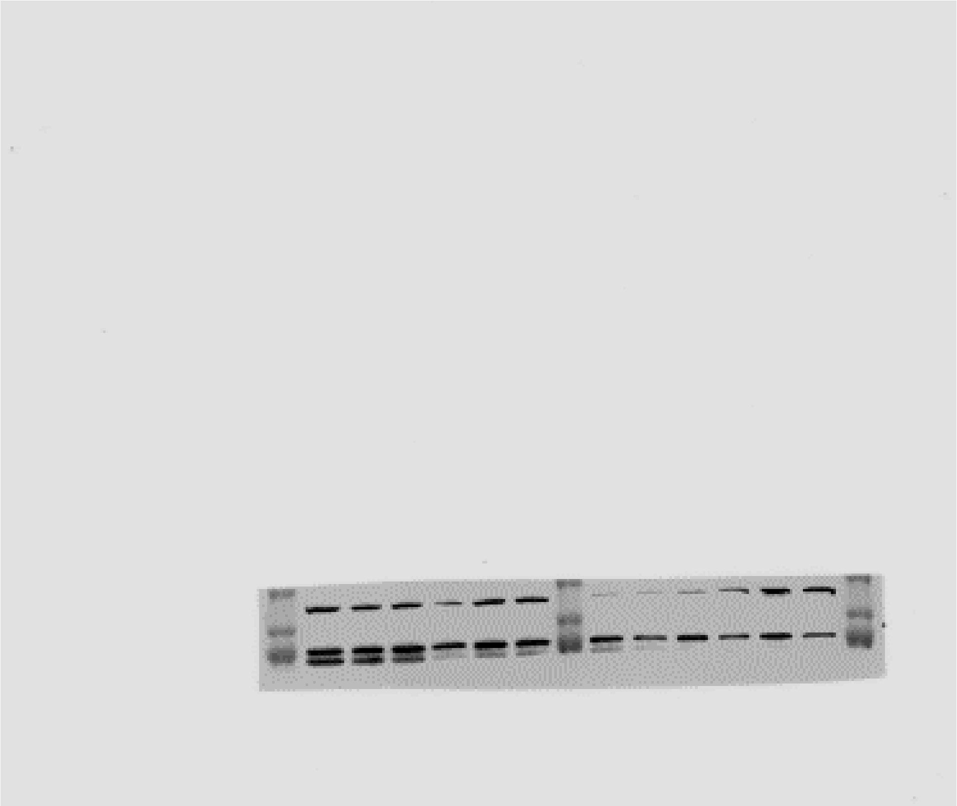

Supplementary Fig. 10B anti-Tubulin (Left)

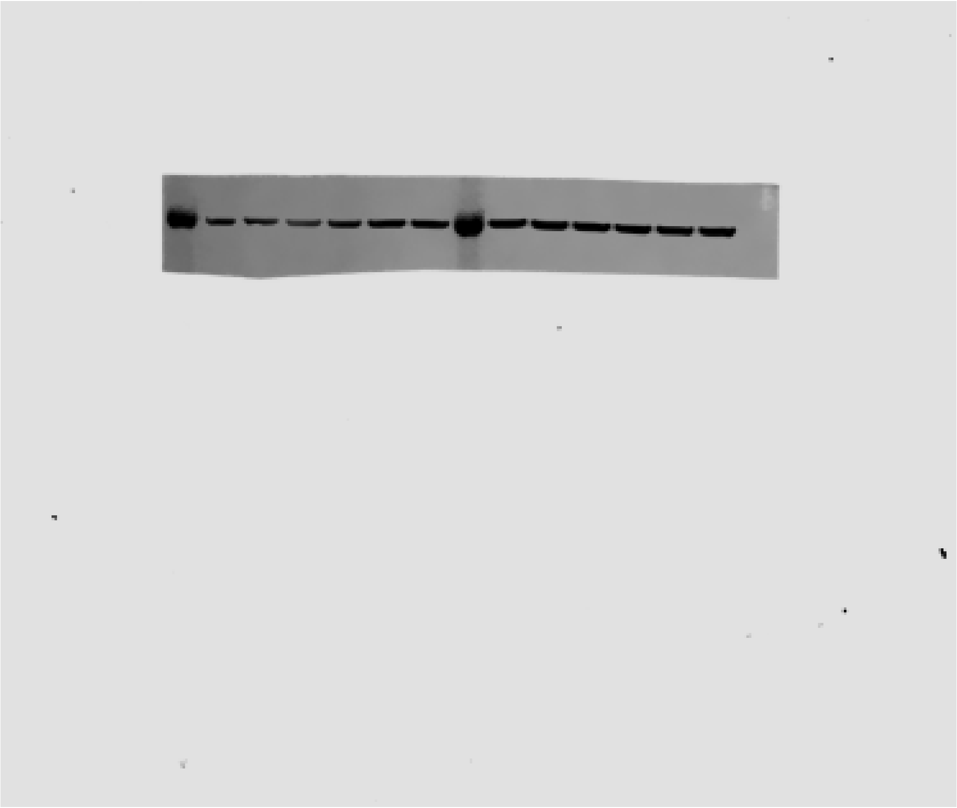

Supplementary Fig. 10L anti-MyHC (Left)

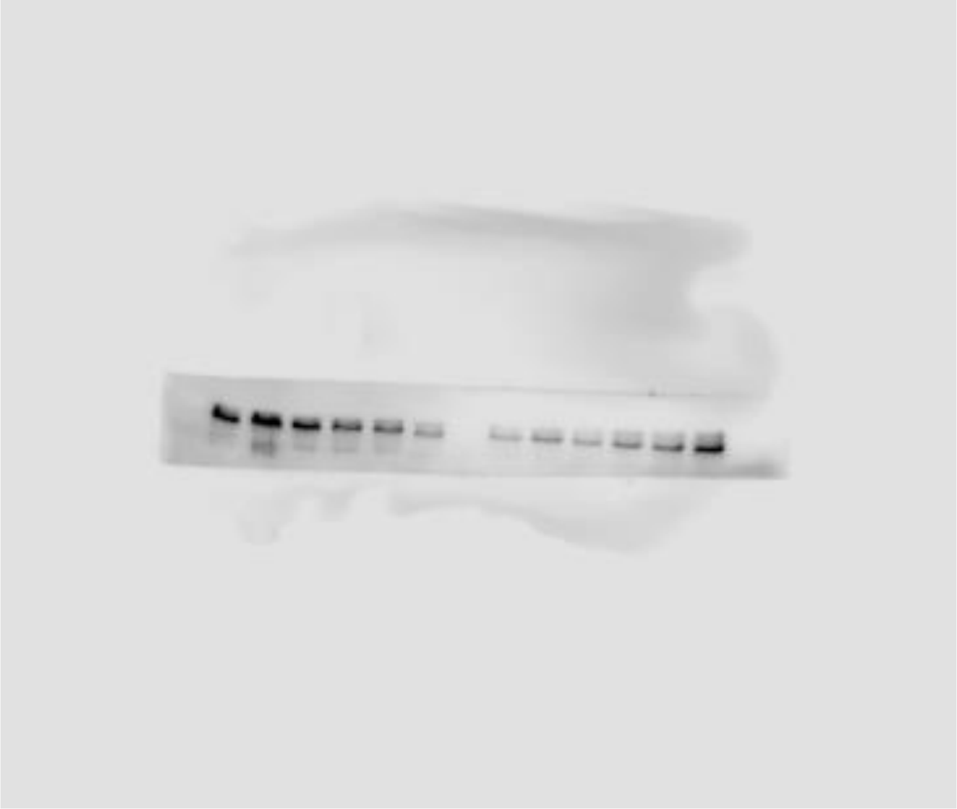

Supplementary Fig. 10L anti-MYOD (Left)

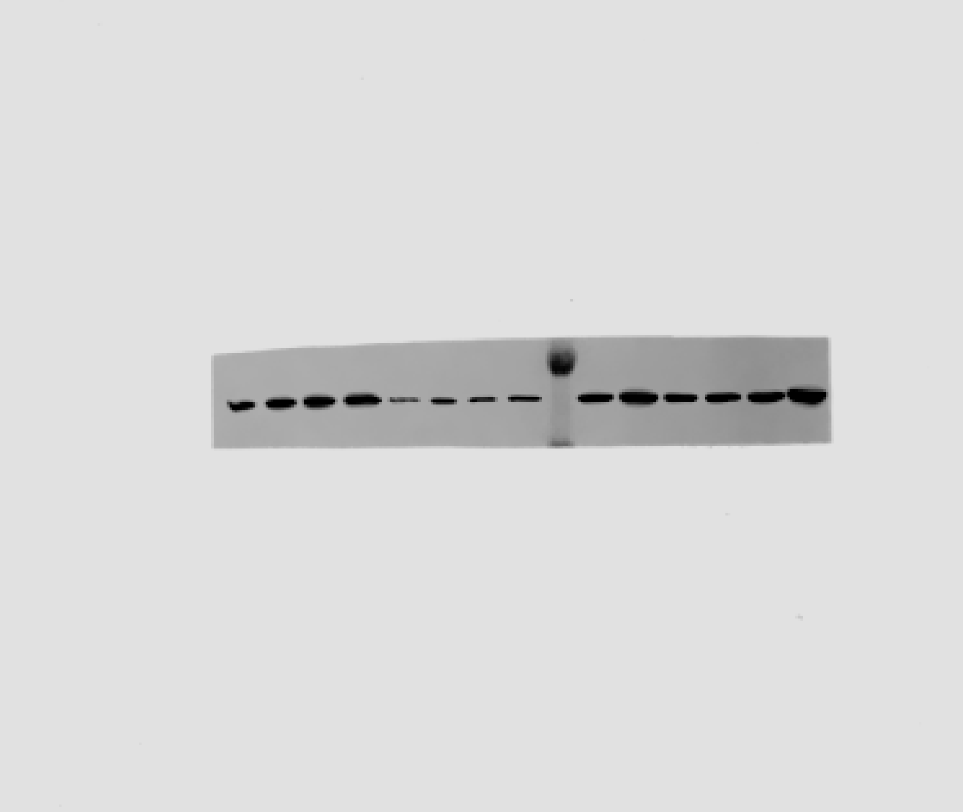

Supplementary Fig. 10L anti-Tubulin (Right)

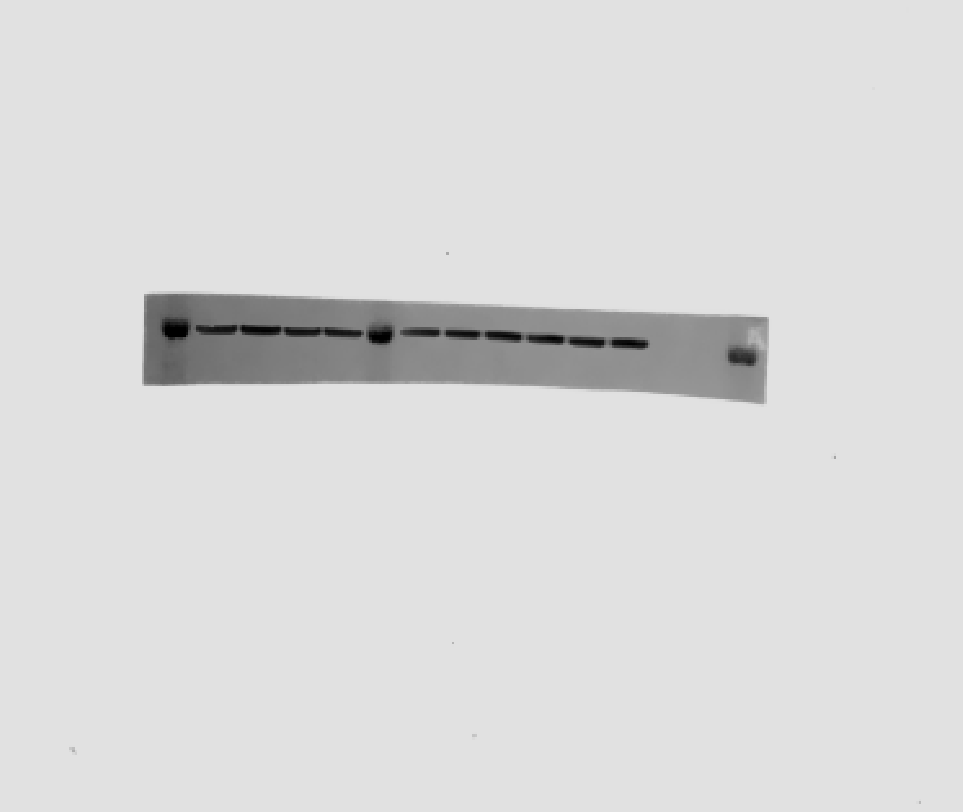

Supplementary Fig. 10N anti-PC (Right)

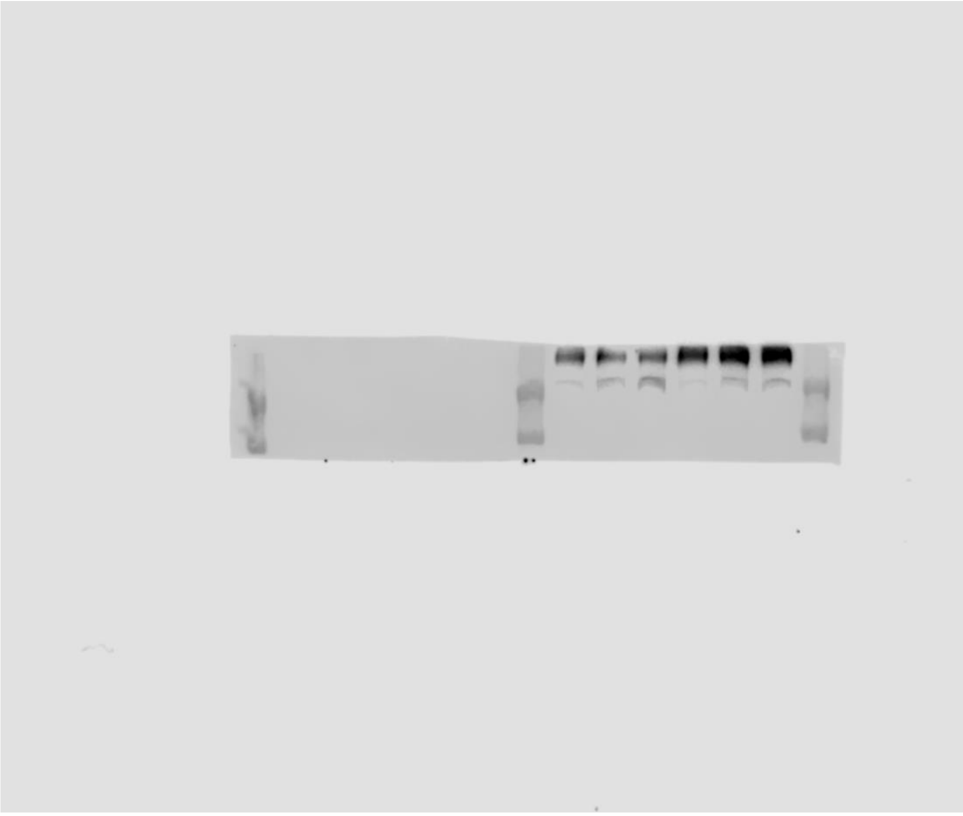

Supplementary Fig. 10N anti-Tubulin (Right)

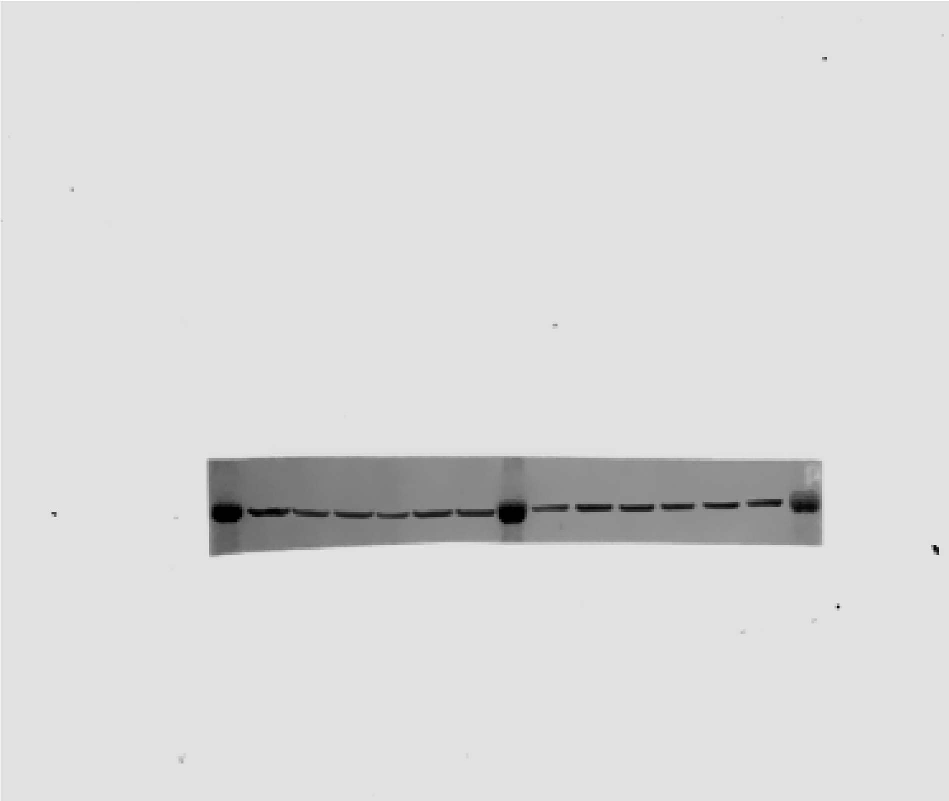

Supplementary Fig. 10X anti-MyHC (Right)

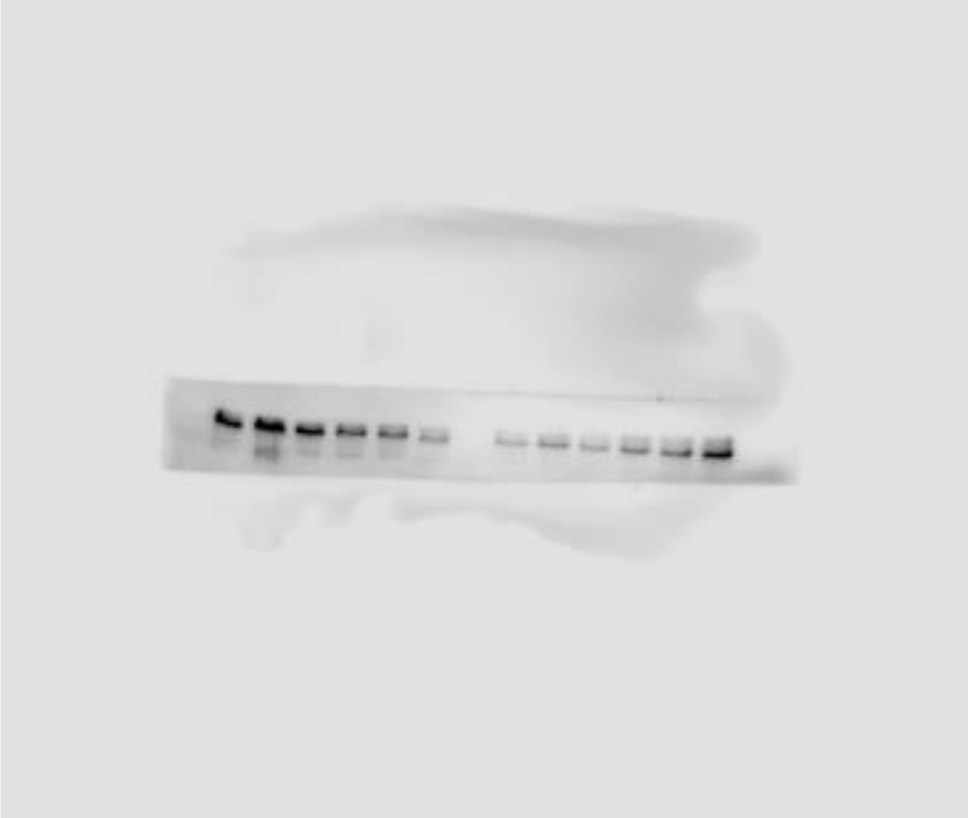

Supplementary Fig. 10X anti-MYOD

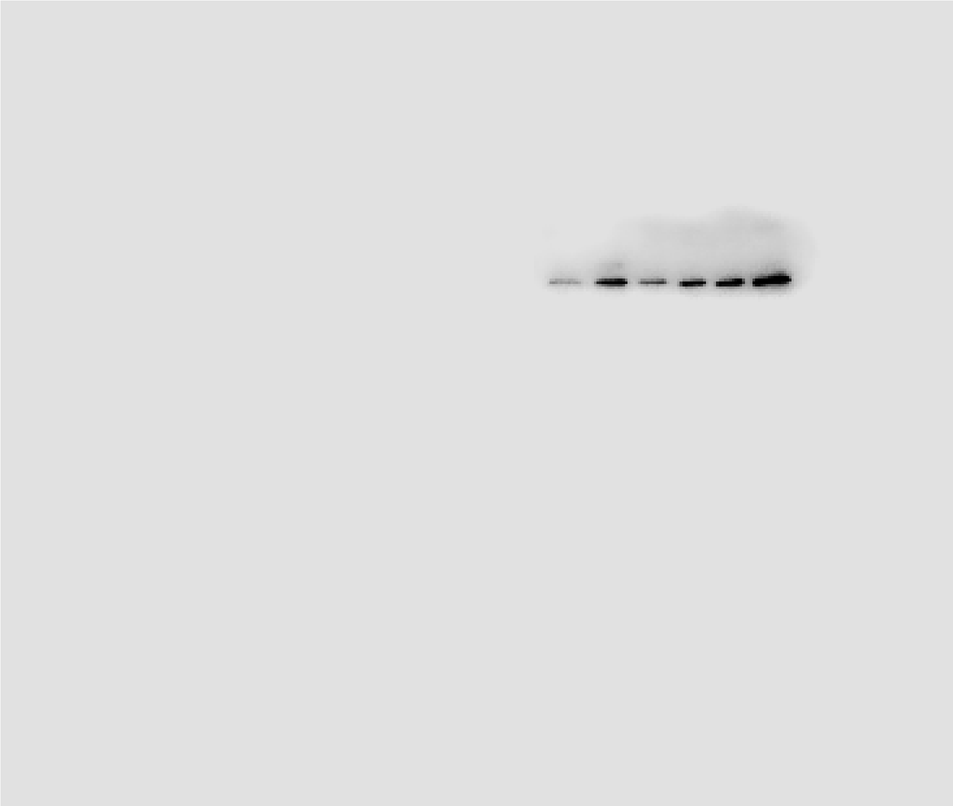

Supplementary Fig. 10X anti-Tubulin (Left)

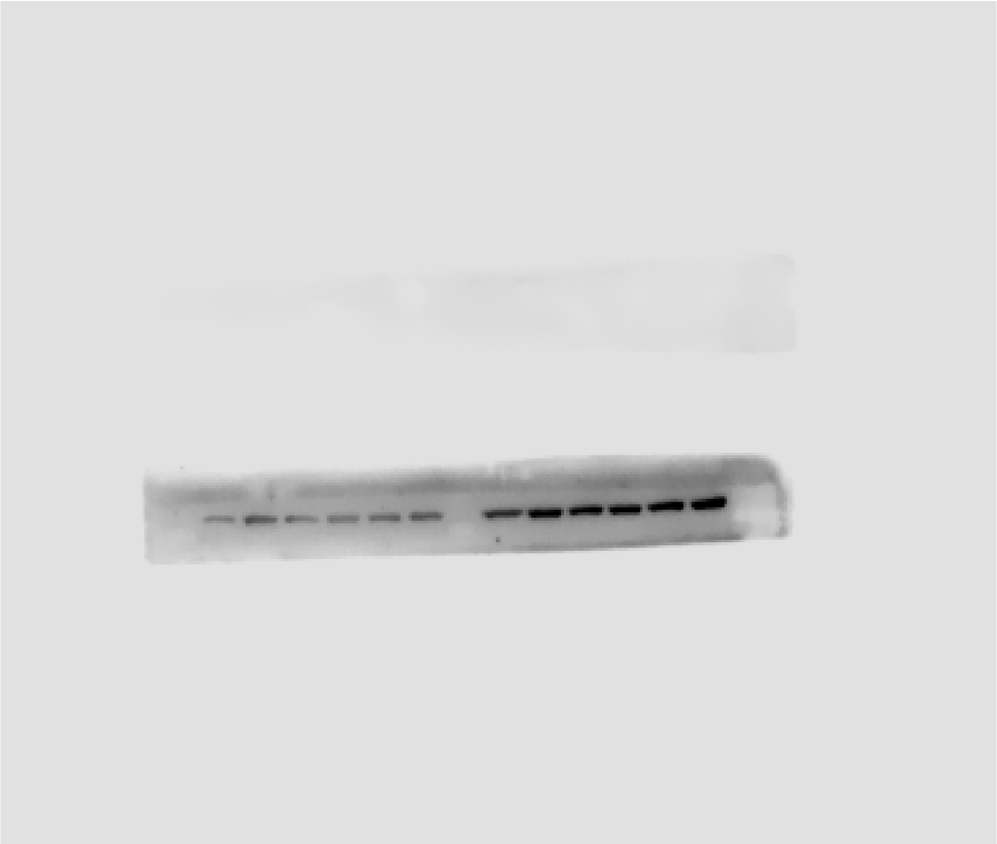

Supplement: Supplementary file 19 — Original Data of western blots [file 41419_2022_4772_MOESM19_ESM.pdf]
